# Dirozalkib versus Crizotinib in Advanced Anaplastic Lymphoma Kinase Inhibitor-Naïve *ALK*-Positive Non-Small-Cell Lung Cancer: A Multicenter, Randomized Phase III Trial

**DOI:** 10.34133/cancomm.0068

**Published:** 2026-11-10

**Authors:** Ying Liu, Xiangjiao Meng, Yan Yu, Jianhua Chen, Runxiang Yang, Laiyu Liu, Yanqiu Zhao, Ruifen Tian, Dianming Li, Ruiling Ning, Qingshan Li, Guowu Wu, Li Wang, Xianghui Duan, Xuejie Guo, Yan Hu, Weili Liu, Haifeng Liu

**Affiliations:** ^1^ Department of Oncology, Jilin Provincial Cancer Hospital, Changchun, Jilin, P. R. China.; ^2^ Department of Radiation Oncology, Shandong Cancer Hospital and Institute, Shandong First Medical University and Shandong Academy of Medical Sciences, Jinan, Shandong, P. R. China.; ^3^ Department of Thoracic Medical Oncology, The Affiliated Cancer Hospital of Harbin Medical University, Harbin, Heilongjiang, P. R. China.; ^4^ Department of Thoracic Medical Oncology, Hunan Provincial Cancer Hospital, Changsha, Hunan, P. R. China.; ^5^ Department of Medical Oncology, Yunnan Provincial Cancer Hospital, Kunming, Yunnan, P. R. China.; ^6^ Department of Respiratory and Critical Care Medicine, Nanfang Hospital Affiliated to Southern Medical University, Guangzhou, Guangdong, P. R. China.; ^7^ Department of Thoracic Medical Oncology, The Affiliated Cancer Hospital of Zhengzhou University & Henan Cancer Hospital, Zhengzhou, Henan, P. R. China.; ^8^ Department of Respiratory Oncology, Shanxi Provincial Cancer Hospital, Taiyuan, Shanxi, P. R. China.; ^9^ Department of Respiratory and Critical Care Medicine, The First Affiliated Hospital of Bengbu Medical College, Bengbu, Anhui, P. R. China.; ^10^ Department of Respiratory Medical Oncology, The Affiliated Cancer Hospital of Guangxi Medical University, Nanning, Guangxi Zhuang Autonomous Region, P. R. China.; ^11^ Department of Oncology, The Affiliated Hospital of Chengde Medical University, Chengde, Hebei, P. R. China.; ^12^ Department of Medical Oncology, Meizhou People’s Hospital, Meizhou, Guangdong, P. R. China.; ^13^ Department of Clinical Science, Xuanzhu Biopharmaceutical Co. Ltd., Beijing, P. R. China.

## Abstract

**Background:** Dirozalkib is a highly selective anaplastic lymphoma kinase (*ALK*) inhibitor with intracranial antitumor activity against crizotinib-resistant mutations. This phase III trial evaluated dirozalkib versus crizotinib in patients with ALK inhibitor-naïve, *ALK*-positive, advanced non-small-cell lung cancer (NSCLC). **Methods:** In this multicenter, randomized, open-label DIAMOND-2 trial, adult patients with ALK inhibitor-naïve, *ALK*-positive, advanced NSCLC who either had received no prior systemic therapy or had experienced disease progression or intolerance after one prior chemotherapy regimen were randomly assigned 1:1 to dirozalkib (500 mg once daily) or crizotinib (250 mg twice daily). The primary end point was investigator-assessed progression-free survival (PFS). **Results:** Between 2022 January 24 and 2023 April 30, 275 patients were randomly assigned to receive dirozalkib (*n* = 137) or crizotinib (*n* = 138). As of 2025 May 31, the median follow-up durations were 32.8 and 31.6 months, respectively. Median investigator-assessed PFS was 29.5 (95% confidence interval [CI] = 20.3 to not reached) months with dirozalkib versus 12.9 (95% CI = 11.1 to 17.6) months with crizotinib (hazard ratio [HR] = 0.47, 95% CI = 0.34 to 0.66; *P* < 0.001). Blinded-independent-central-review-assessed PFS was also significantly prolonged with dirozalkib (median PFS, 27.5 versus 12.9 months; HR = 0.54, 95% CI = 0.39 to 0.74; *P* < 0.001). Grade ≥3 treatment-emergent adverse events (TEAEs) were less frequent with dirozalkib than with crizotinib (60.6% versus 72.5%), and TEAEs led to treatment discontinuation in 2.2% and 7.2% of patients, respectively. **Conclusions:** Dirozalkib significantly improved PFS versus crizotinib, with a manageable safety profile, supporting its use as a first-line therapy for *ALK*-positive advanced NSCLC. **Trial registration:** This trial was registered at ClinicalTrials.gov on 2022 January 24 (NCT05204628).

## Background

Lung cancer remains the leading cause of cancer-related death worldwide, with non-small-cell lung cancer (NSCLC) accounting for approximately 85% of cases [1]. Targeted therapy plays a pivotal role in the management of NSCLC by targeting actionable genomic alterations that drive tumor growth and proliferation [2,3]. Among these alterations, anaplastic lymphoma kinase (*ALK*) gene rearrangements represent an important oncogenic driver and actionable therapeutic target in NSCLC [4,5]. Patients with *ALK*-rearranged NSCLC generally derive clinical benefit from treatment with ALK tyrosine kinase inhibitors [6,7]. First-generation ALK inhibitors, such as crizotinib, represented a milestone in the treatment of *ALK*-rearranged NSCLC but provided relatively modest disease control, with median progression-free survival (PFS) ranging from 7 to 11 months in patients with previously untreated *ALK*-positive NSCLC despite relatively high objective response rates (ORRs) of approximately 60% to 74% [8,9]. This limited clinical benefit largely reflects the emergence of acquired resistance, with a limited duration of response (DoR) and disease progression commonly occurring within the first year of therapy [8,9]. These limitations highlight the need for novel ALK inhibitors that provide more durable responses and improve clinical outcomes in patients with *ALK*-positive NSCLC.

*ALK*-positive advanced NSCLC carries a substantial lifetime risk of central nervous system (CNS) involvement, with 20% to 30% of patients presenting with brain metastases at diagnosis [10,11] and cumulative incidences of 45.5% and 58.4% at 2 and 3 years after diagnosis, respectively [12]. The presence of CNS metastases in *ALK*-positive NSCLC is associated with poor overall survival (OS) and diminished quality of life [10]. Brain metastases frequently contribute to treatment failure and disease progression because many systemic therapies have limited penetration across the blood–brain barrier (BBB) [13,14]. First-generation ALK inhibitors have poor CNS penetration, primarily because they are substrates for P-glycoprotein-mediated efflux across the BBB [15,16]. Notably, following crizotinib treatment, progression of brain metastases occurs in up to 50% to 60% of patients [17]. Therefore, novel ALK inhibitors that effectively control brain metastases while maintaining a favorable CNS safety profile are warranted.

Dirozalkib is a novel, next-generation, highly selective ALK inhibitor that has demonstrated potent inhibitory activity against a broad spectrum of resistance-associated *ALK* mutations, including mutations associated with resistance to first- and second-generation ALK inhibitors, such as C1156Y, L1196M, F1174L, G1202R, and I1171N/T/S. Notably, dirozalkib retains potent activity in cell lines harboring alectinib-resistant mutations and has demonstrated greater in vivo efficacy than ceritinib in crizotinib-resistant patient-derived xenograft models, as well as potent intracranial antitumor activity. A phase I clinical trial (NCT05055232) demonstrated promising antitumor activity and a favorable safety and tolerability profile with dirozalkib in patients with *ALK*-rearranged advanced NSCLC, particularly among ALK inhibitor-naïve patients [18]. Thus, the present phase III, multicenter, randomized, open-label clinical trial (DIAMOND-2) aimed to evaluate the efficacy and safety of dirozalkib versus crizotinib in patients with ALK inhibitor-naïve, *ALK*-positive advanced NSCLC.

## Materials and Methods

### Study design and patients

The DIAMOND-2 trial (NCT05204628) was a phase III, multicenter, randomized, open-label study comparing dirozalkib with crizotinib in ALK inhibitor-naïve patients with *ALK*-positive advanced NSCLC. The trial was initiated at 64 hospitals in China, of which 52 ultimately enrolled participants. The study was conducted in accordance with the principles of the Declaration of Helsinki and Good Clinical Practice guidelines. The protocol (Data S1) was approved by the ethics committee of the lead center, Jilin Province Cancer Hospital (202111-106-07), as well as by the ethics committees at each participating site. All patients provided written informed consent before enrollment. An independent data monitoring committee periodically reviewed safety data and study conduct to ensure participant safety and the integrity of the trial.

Eligible participants were adults aged 18 to 75 years with pathologically confirmed advanced NSCLC and *ALK*-positive status confirmed by immunohistochemistry, fluorescence in situ hybridization, polymerase chain reaction, or next-generation sequencing (NGS). Eligible participants had not previously received an ALK inhibitor and either had not received systemic anticancer therapy or had received one prior chemotherapy regimen but subsequently experienced disease progression or intolerance. Participants were required to have an Eastern Cooperative Oncology Group (ECOG) performance status of 0 to 2 and at least one measurable extracranial lesion according to the Response Evaluation Criteria in Solid Tumors (RECIST) version 1.1 that had not been previously irradiated. Patients without brain metastases or with asymptomatic, untreated brain metastases were eligible. Key exclusion criteria included small cell lung cancer, uncontrolled malignant pleural, peritoneal, or pericardial effusion that reaccumulated markedly within 1 week after drainage, or active infections deemed to pose safety concerns. Detailed inclusion and exclusion criteria are provided in the study protocol (Data S1).

### Treatment and procedures

Participants were randomly assigned in a 1:1 ratio to receive either dirozalkib (500 mg orally, once daily) or crizotinib (250 mg orally, twice daily) using stratified block randomization through an interactive web response system. The stratification factors were the presence of brain metastases (yes versus no) and prior chemotherapy (yes versus no). Each treatment cycle lasted 28 d, and treatment continued until disease progression, unacceptable toxicity, withdrawal of consent, death, or discontinuation at the discretion of the investigator or patient, whichever occurred first. Dose adjustments, treatment interruptions, or permanent discontinuation of dirozalkib or crizotinib was permitted in the event of adverse events (AEs).

Radiologic assessments using computed tomography and/or MRI were performed at baseline, every 8 weeks during the first 64 weeks of treatment, and every 12 weeks thereafter. Tumor responses were evaluated according to RECIST version 1.1 and the Response Assessment in Neuro-Oncology Brain Metastases criteria. AEs were monitored from the time of informed consent until at least 30 d after the final dose of study treatment and were graded according to the Common Terminology Criteria for Adverse Events version 5.0. AE monitoring included assessments of vital signs, physical examinations, echocardiography, electrocardiography, and laboratory parameters.

### Outcomes

The primary end point was investigator-assessed PFS, defined as the time from randomization to documented disease progression or death from any cause, whichever occurred first. Secondary end points included PFS assessed by blinded independent central review (BICR), ORR, disease control rate (DCR), DoR, OS, intracranial ORR, intracranial DoR, and safety. The cumulative incidence of intracranial progression (ICP) and investigator-assessed intracranial PFS were evaluated in post hoc analyses.

### Statistical analysis

A parallel-group superiority design was used, assuming a median PFS of 11.0 months with crizotinib and 20.0 months with dirozalkib. With a one-sided type I error rate of 0.025 and 95% power, 147 PFS events were required to detect a hazard ratio (HR) of 0.550. Assuming an accrual period of approximately 18.0 months and a follow-up duration of 20.0 months after the last patient was randomized, an interim analysis was planned when 70% of the required PFS events had occurred. The α-spending function for the interim analysis followed the O’Brien–Fleming approach. Based on these assumptions, a total of 201 participants were required. To account for a potential dropout rate of 15%, the target enrollment was increased to at least 238 patients (119 per group). Because the study was conducted during the COVID-19 pandemic, the planned enrollment was increased to approximately 268 participants to accommodate an expected dropout rate of 25%, while the required number of PFS events remained unchanged. We report the final analysis of PFS, which was conducted after the prespecified number of PFS events had occurred.

Efficacy analyses were conducted in the intention-to-treat (ITT) population, which included all randomized participants. Additional post hoc analyses were also performed in the ITT population. The modified ITT (mITT) population, used for a post hoc efficacy analysis, comprised participants in the ITT population for whom information about the regulatory approval status of the NGS kits used for baseline *ALK* rearrangement testing was available. Safety analyses were performed in the as-treated population, which included all participants who received at least one dose of study treatment.

Time-to-event end points were estimated using the Kaplan–Meier method, and between-group comparisons were performed using one-sided stratified log-rank tests. HRs and corresponding 95% confidence intervals (CIs) were estimated using Cox proportional hazards regression models. The Cochran–Mantel–Haenszel test was used to compare ORR and DCR between treatment groups, and corresponding 95% CIs were calculated for each treatment group. All statistical analyses were performed using SAS, version 9.4 or later (SAS Institute, Cary, NC, USA). Two-sided *P* < 0.05 was considered statistically significant.

## Results

### Patients and disposition

Between 2022 January 24 and 2023 April 30, a total of 275 patients at 52 hospitals were enrolled and randomly assigned to receive either dirozalkib (*n* = 137) or crizotinib (*n* = 138) (Fig. 1). Baseline demographic and clinical characteristics were generally comparable between the 2 groups, including brain metastases (21.2% versus 21.0%), prior brain radiotherapy (1.5% versus 1.4%), and prior chemotherapy for advanced disease (8.8% versus 10.9%; Table 1). At the data cutoff (2025 May 31), 76 patients (55.5%) in the dirozalkib group and 110 patients (79.7%) in the crizotinib group had discontinued treatment, whereas 61 and 28 patients, respectively, remained on treatment.

**Fig. 1.**
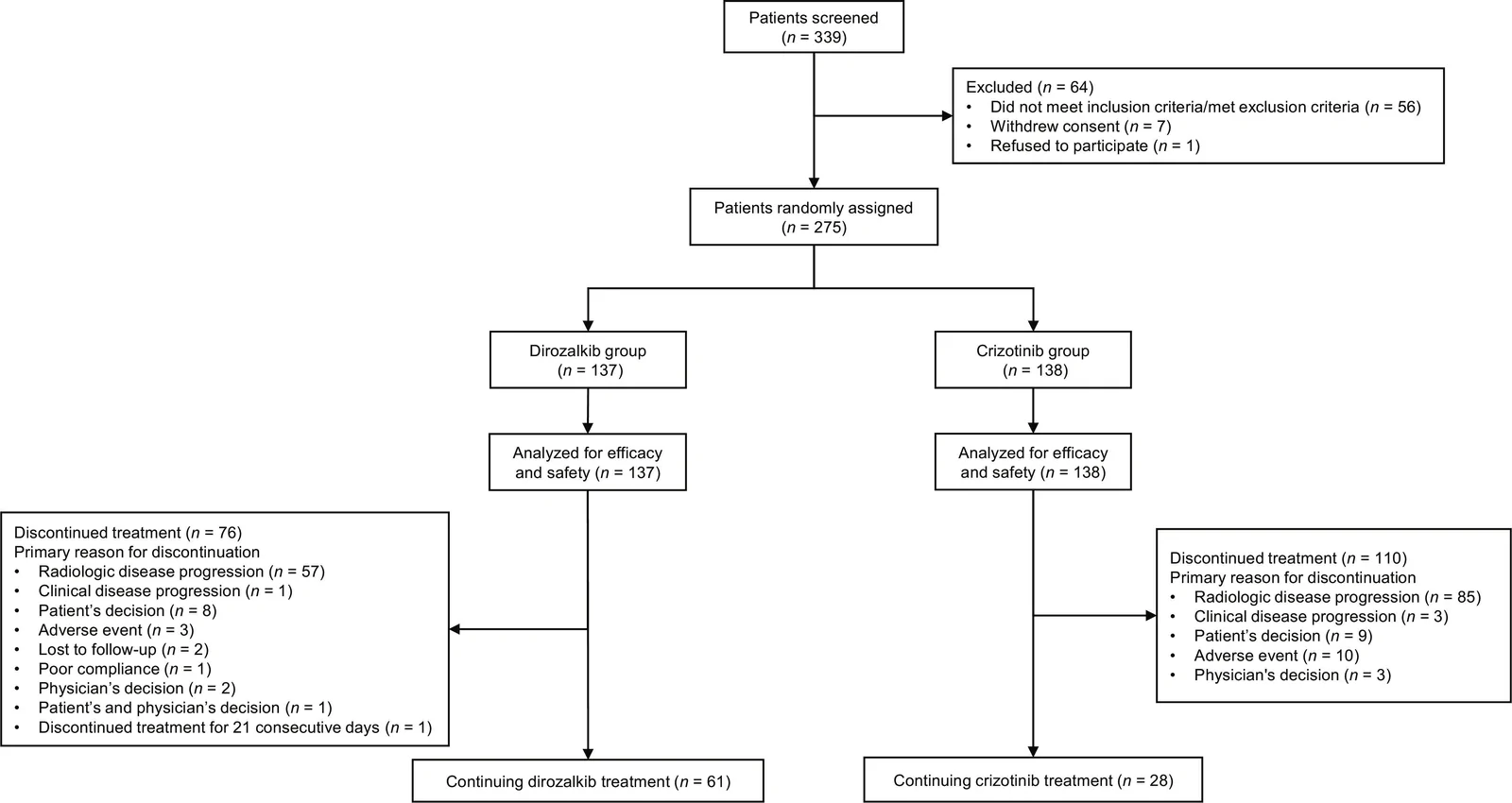
CONSORT flow diagram. Of 339 patients screened, 275 were randomly assigned to receive dirozalkib (*n* = 137) or crizotinib (*n* = 138). Reasons for treatment discontinuation are shown for each treatment group. At the time of analysis, 61 patients remained on dirozalkib treatment and 28 remained on crizotinib treatment. All randomized patients were included in the efficacy and safety analyses.

**Table 1.** Baseline patient characteristics

Characteristics	Dirozalkib (*n* = 137)	Crizotinib (*n* = 138)
Age, median (range), y	53 (22–75)	54 (25–74)
<65 y	111 (81.0)	117 (84.8)
≥65 y	26 (19.0)	21 (15.2)
Sex, *n* (%)		
Male	74 (54.0)	66 (47.8)
Female	63 (46.0)	72 (52.2)
ECOG performance status, *n* (%)		
0	34 (24.8)	30 (21.7)
1	100 (73.0)	106 (76.8)
2	3 (2.2)	2 (1.4)
Smoking status, *n* (%)		
Yes	39 (28.5)	35 (25.4)
No	98 (71.5)	103 (74.6)
Disease stage, *n* (%)		
IIIB	5 (3.6)	5 (3.6)
IIIC	5 (3.6)	3 (2.2)
IV	127 (92.7)	130 (94.2)
Histological type, *n* (%)		
Adenocarcinoma	134 (97.8)	132 (95.7)
Squamous cell carcinoma	3 (2.2)	3 (2.2)
Adenosquamous carcinoma	0 (0.0)	1 (0.7)
Other	0 (0.0)	2 (1.4)
Previous chemotherapy, *n* (%)	12 (8.8)	15 (10.9)
Brain metastases at baseline, *n* (%)	29 (21.2)	29 (21.0)
Previous brain radiotherapy, *n* (%)	2 (1.5)	2 (1.4)

ECOG, Eastern Cooperative Oncology Group.

### Efficacy among the ITT population

As of 2025 May 31, the median follow-up durations in the ITT population were 32.8 (range, 2.2 to 39.0) months in the dirozalkib group and 31.6 (range, 0.3 to 39.5) months in the crizotinib group. A total of 158 PFS events had occurred, including 63 events (46.0%) in the dirozalkib group and 95 events (68.8%) in the crizotinib group. As shown in Fig. 2A, the median investigator-assessed PFS was 29.5 (95% CI = 20.3 to not reached [NR]) months in the dirozalkib group and 12.9 (95% CI = 11.1 to 17.6) months in the crizotinib group (HR = 0.47, 95% CI = 0.34 to 0.66; *P* < 0.001). The PFS benefit with dirozalkib was observed in patients both with and without baseline brain metastases. Among patients with baseline brain metastases, median PFS was significantly longer with dirozalkib than with crizotinib (18.5 versus 9.2 months; HR = 0.38, 95% CI = 0.20 to 0.72; *P* = 0.001). Among patients without baseline brain metastases, median PFS was not reached (95% CI = 25.8 to NR) with dirozalkib versus 17.6 (95% CI = 12.9 to 22.9) months with crizotinib (HR = 0.50, 95% CI = 0.34 to 0.73; *P* < 0.001). PFS benefits favoring dirozalkib over crizotinib were generally observed across prespecified subgroups (Fig. 2B). BICR-assessed PFS in the ITT population was also significantly longer with dirozalkib than with crizotinib (median PFS, 27.5 versus 12.9 months; HR = 0.54, 95% CI = 0.39 to 0.74; *P* < 0.001) (Fig. 2C).

**Fig. 2.**
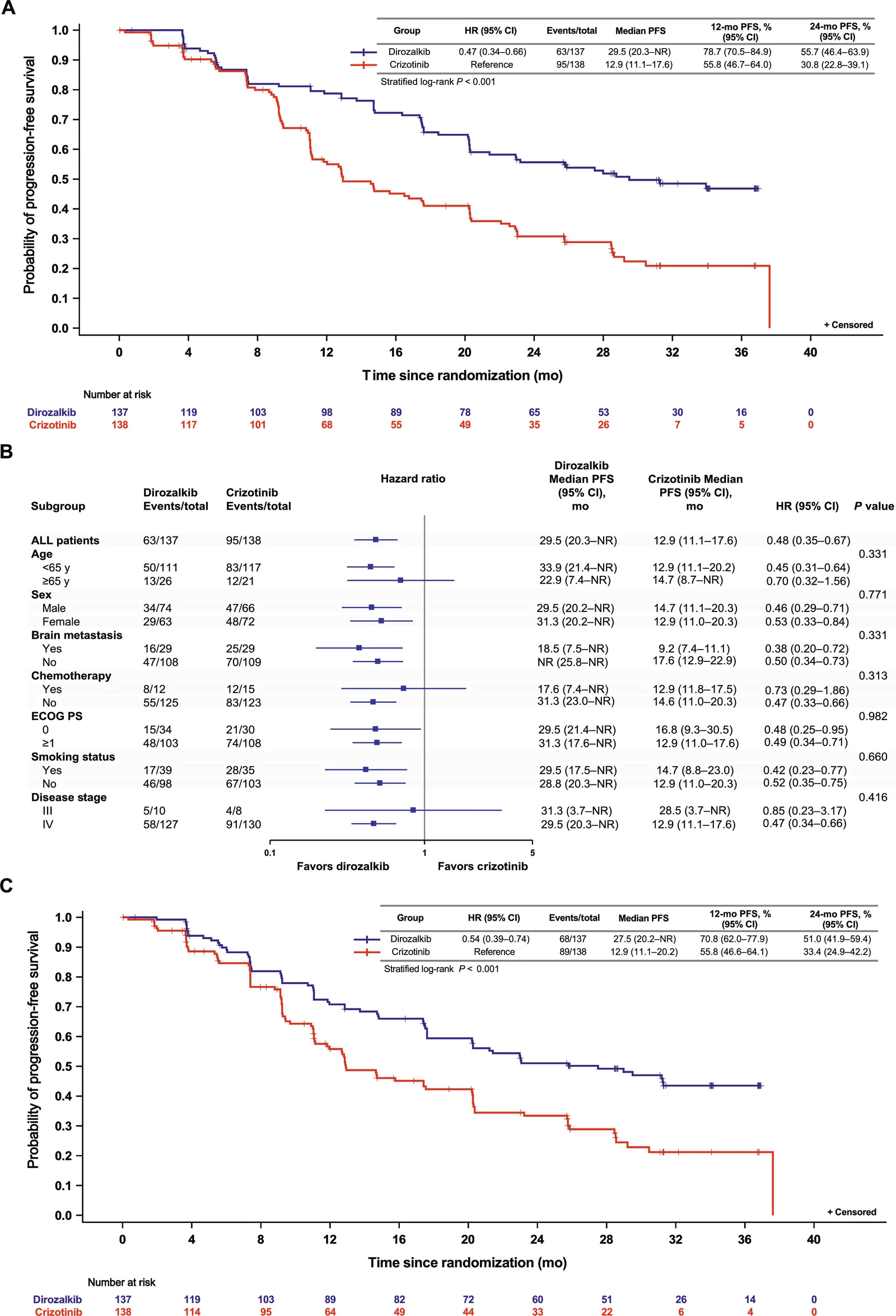
Progression-free survival with dirozalkib versus crizotinib. (A) Kaplan–Meier curves of progression-free survival assessed by the investigators in the intent-to-treat population. (B) Forest plot of investigator-assessed progression-free survival in subgroup analyses of the intent-to-treat population. (C) Kaplan–Meier curves of progression-free survival assessed by the blinded independent central review in the intent-to-treat population. ECOG PS, Eastern Cooperative Oncology Group performance status; HR, hazard ratio; CI, confidence interval.

As shown in Table 2, the investigator-assessed ORR was 89.1% (95% CI = 82.6% to 93.7%) in the dirozalkib group and 81.9% (95% CI = 74.4% to 87.9%) in the crizotinib group. The median DoR was 29.5 (95% CI = 19.4 to NR) months with dirozalkib and 11.1 (95% CI = 9.3 to 18.5) months with crizotinib (HR = 0.45, 95% CI = 0.32 to 0.64; *P* < 0.001). The BICR-assessed ORR was 89.8% (95% CI = 83.5% to 94.3%) in the dirozalkib group and 89.1% (95% CI = 82.7% to 93.8%) in the crizotinib group. The BICR-assessed median DoR was 25.7 months with dirozalkib and 12.9 months with crizotinib (HR = 0.52, 95% CI = 0.37 to 0.74; *P* < 0.001).

**Table 2.** Tumor response per investigator and blinded independent central review in the intention-to-treat population

Tumor response	Dirozalkib (*n* = 137)	Crizotinib (*n* = 138)
Best overall response per investigator		
CR, *n* (%)	0 (0.0)	0 (0.0)
PR, *n* (%)	122 (89.1)	113 (81.9)
SD, *n* (%)	9 (6.6)	16 (11.6)
PD, *n* (%)	1 (0.7)	6 (4.3)
Not evaluable, *n* (%)	5 (3.6)	3 (2.2)
ORR per investigator (95% CI), %	89.1 (82.6–93.7)	81.9 (74.4–87.9)
DCR per investigator (95% CI), %	95.6 (90.7–98.4)	93.5 (88.0–97.0)
DoR per investigator (95% CI), months	29.5 (19.4–NR)	11.1 (9.3–18.5)
ORR per BIRC (95% CI), %	89.8 (83.5–94.3)	89.1 (82.7–93.8)
DCR per BIRC (95% CI), %	95.6 (90.7–98.4)	94.2 (88.9–97.5)
DoR per BIRC (95% CI), mo	25.7 (16.6–NR)	12.9 (10.2–18.5)

CR, complete response; PR, partial response; SD, stable disease; PD, disease progression; CI, confidence interval; ORR, objective response rate; DCR, disease control rate; DoR, duration of response; NR, not reached; BIRC, blinded independent central review.

Among patients with measurable brain metastases at baseline, the investigator-assessed intracranial ORR was significantly higher in the dirozalkib group than in the crizotinib group (92.3% versus 11.1%; *P* < 0.001). The median intracranial DoR was also markedly longer with dirozalkib than with crizotinib (18.4 versus 3.6 months; *P* < 0.001) (Table S1). In a post hoc analysis of investigator-assessed intracranial PFS in the ITT population regardless of baseline brain metastasis status, intracranial PFS was significantly longer with dirozalkib than with crizotinib (median, NR versus 29.2 months; HR = 0.47, 95% CI = 0.30 to 0.74; *P* < 0.001), and the 2-year cumulative incidence of ICP was also significantly lower with dirozalkib than with crizotinib (26.1% [95% CI = 18.8% to 35.7%] versus 42.5% [95% CI = 33.3% to 53.2%]; *P* = 0.001). The investigator-assessed median intracranial PFS for patients with any brain metastases was 20.3 months (95% CI = 12.9 to NR) in the dirozalkib group and 10.8 months (95% CI = 7.9 to 12.8) in the crizotinib group (HR = 0.42, 95% CI = 0.21 to 0.84; *P* = 0.006).

At the data cutoff, OS data remained immature, with 66 deaths reported: 32 (23.4%) in the dirozalkib group and 34 (24.6%) in the crizotinib group. Information about subsequent anticancer therapies administered after disease progression is summarized in Table S2. Among patients who discontinued study treatment after disease progression, 54 of 63 patients (85.7%) in the dirozalkib group and 83 of 92 patients (90.2%) in the crizotinib group received subsequent systemic therapy. In the crizotinib group, 82.6% (76 of 92) of patients received further ALK-targeted therapy, whereas the corresponding proportion was 77.8% (49/63) in the dirozalkib group.

### Efficacy among the mITT population

In the post hoc analysis of the mITT population, 131 patients in the dirozalkib group and 136 patients in the crizotinib group were included. As of 2025 May 31, 152 PFS events had occurred in the mITT population. The median PFS was 31.3 (95% CI = 22.9 to NR) months with dirozalkib and 12.9 (95% CI = 11.1 to 17.6) months with crizotinib (HR = 0.47, 95% CI = 0.33 to 0.65; *P* < 0.001). The investigator-assessed ORR was 88.5% in the dirozalkib group and 81.6% in the crizotinib group. The median DoR was 32.1 months with dirozalkib and 11.1 months with crizotinib (HR = 0.44, 95% CI = 0.31 to 0.63; *P* < 0.001).

Among patients with measurable brain metastases at baseline, the intracranial ORR was 91.7% in the dirozalkib group and 11.1% in the crizotinib group. The median intracranial DoR was NR with dirozalkib versus 3.6 months with crizotinib (HR = 0.00, 95% CI = 0.00 to NR; *P* = 0.001). In the mITT population, the median intracranial PFS was NR with dirozalkib versus 29.2 months with crizotinib (HR = 0.45, 95% CI = 0.28 to 0.71; *P* < 0.001).

### Safety

During the treatment period, the median durations of exposure to dirozalkib and crizotinib were 715 (range, 4 to 1,187) d and 391 (range, 3 to 1,200) d, respectively, with corresponding median relative dose intensities of 99.6% and 98.3%. Treatment-emergent AEs (TEAEs) were reported in all patients in both groups. The most frequently observed TEAEs (incidence of ≥10% in either group and an absolute difference of ≥5% between the 2 groups) are summarized in Table 3. The most common TEAEs among patients receiving dirozalkib were diarrhea (97.8%), vomiting (88.3%), nausea (67.2%), increased alanine aminotransferase (62.0%), increased aspartate aminotransferase (59.1%), increased blood creatine kinase-MB (46.7%; the MB isoform, composed of M-type and B-type subunits), and weight loss (46.0%), most of which were grade 1 or 2. Grade ≥3 TEAEs occurred in 60.6% of patients in the dirozalkib group and 72.5% of those in the crizotinib group. The most common grade ≥3 TEAEs in the dirozalkib group were diarrhea (19.0%), increased γ-glutamyl transferase (9.5%), hypertriglyceridemia (7.3%), hyperglycemia (6.6%), increased alanine aminotransferase (5.8%), QT interval prolongation (5.8%), and hypokalemia (5.8%) (Table 3). Treatment-emergent serious AEs were reported in 21.2% of patients treated with dirozalkib and 26.8% of those treated with crizotinib. The incidence of treatment-related serious AEs was similar between the 2 groups (13.9% versus 12.3%) (Table S3).

**Table 3.** Treatment-emergent adverse events reported in ≥10% of patients in either treatment group, or with an absolute difference of ≥5 percentage points between the 2 groups

Adverse events, *n* (%)	Dirozalkib (*n* = 137)		Crizotinib (*n* = 138)	
	Any grade	Grade ≥3	Any grade	Grade ≥3
**Any TEAE**	137 (100)	83 (60.6)	138 (100)	100 (72.5)
Diarrhea	134 (97.8)	26 (19.0)	75 (54.3)	1 (0.7)
Vomiting	121 (88.3)	4 (2.9)	65 (47.1)	0 (0.0)
Nausea	92 (67.2)	6 (4.4)	50 (36.2)	1 (0.7)
ALT increase	85 (62.0)	8 (5.8)	105 (76.1)	18 (13.0)
AST increase	81 (59.1)	7 (5.1)	102 (73.9)	14 (10.1)
Blood CK-MB increase	64 (46.7)	0 (0.0)	88 (63.8)	16 (11.6)
Weight loss	63 (46.0)	3 (2.2)	19 (13.8)	0 (0.0)
Hyperuricemia	58 (42.3)	0 (0.0)	35 (25.4)	0 (0.0)
Hypertriglyceridemia	52 (38.0)	10 (7.3)	35 (25.4)	4 (2.9)
Anemia	52 (38.0)	6 (4.4)	35 (25.4)	2 (1.4)
Decreased appetite	47 (34.3)	3 (2.2)	27 (19.6)	0 (0.0)
Hyperglycemia	46 (33.6)	9 (6.6)	22 (15.9)	0 (0.0)
Blood creatine kinase increase	41 (29.9)	3 (2.2)	86 (62.3)	7 (5.1)
γ-Glutamyltransferase increase	40 (29.2)	13 (9.5)	50 (36.2)	6 (4.3)
Hypercholesterolemia	40 (29.2)	0 (0.0)	29 (21.0)	1 (0.7)
Hypoproteinemia	38 (27.7)	0 (0.0)	65 (47.1)	0 (0.0)
Hypokalemia	30 (21.9)	8 (5.8)	16 (11.6)	5 (3.6)
Sinus bradycardia	30 (21.9)	0 (0.0)	86 (62.3)	5 (3.6)
Blood alkaline phosphatase increase	28 (20.4)	1 (0.7)	45 (32.6)	1 (0.7)
Blood lactate dehydrogenase increase	26 (19.0)	0 (0.0)	58 (42.0)	0 (0.0)
White blood cells decrease	23 (16.8)	1 (0.7)	71 (51.4)	6 (4.3)
Hyponatremia	23 (16.8)	0 (0.0)	14 (10.1)	1 (0.7)
Neutrophil count decrease	23 (16.8)	2 (1.5)	77 (55.8)	38 (27.5)
Weight increase	22 (16.1)	3 (2.2)	46 (33.3)	2 (1.4)
QT interval prolongation	18 (13.1)	8 (5.8)	32 (23.2)	16 (11.6)
Blood bilirubin increase	18 (13.1)	0 (0.0)	11 (8.0)	0 (0.0)
Lipase increase	17 (12.4)	3 (2.2)	27 (19.6)	1 (0.7)
Glycated hemoglobin increase	16 (11.7)	0 (0.0)	7 (5.1)	0 (0.0)
Cough	14 (10.2)	0 (0.0)	25 (18.1)	0 (0.0)
Dizziness	13 (9.5)	0 (0.0)	23 (16.7)	1 (0.7)
Constipation	12 (8.8)	0 (0.0)	51 (37.0)	1 (0.7)
α-Hydroxybutyrate dehydrogenase increase	8 (5.8)	0 (0.0)	33 (23.9)	0 (0.0)
Supraventricular extrasystoles	8 (5.8)	0 (0.0)	18 (13.0)	0 (0.0)
Hypocalcemia	5 (3.6)	0 (0.0)	32 (23.2)	1 (0.7)
Hypoesthesia	5 (3.6)	0 (0.0)	14 (10.1)	0 (0.0)
Visual impairment	5 (3.6)	0 (0.0)	23 (16.7)	0 (0.0)
Chest pain	5 (3.6)	0 (0.0)	16 (11.6)	0 (0.0)
Edema	2 (1.5)	0 (0.0)	44 (31.9)	1 (0.7)
Dysgeusia	1 (0.7)	0 (0.0)	15 (10.9)	0 (0.0)

TEAE, treatment-emergent adverse event; ALT, alanine aminotransferase; AST, aspartate aminotransferase; CK-MB, creatine kinase-MB.

TEAEs led to dose interruptions in 44.5% of patients in the dirozalkib group and 56.5% of those in the crizotinib group (Table S4). COVID-19 infection was the most common TEAE leading to treatment interruption, affecting 16 patients (11.7%) in the dirozalkib group and 17 patients (12.3%) in the crizotinib group. Dose reductions due to TEAEs occurred in 19.7% of dirozalkib-treated patients and 30.4% of crizotinib-treated patients. The most common TEAEs leading to dose reductions in the dirozalkib group were increased alanine aminotransferase (4.4%), diarrhea (3.6%), vomiting (3.6%), increased aspartate aminotransferase (2.9%), and hyperglycemia (2.9%). Discontinuations due to TEAEs occurred in 2.2% of patients in the dirozalkib group and 7.2% of those in the crizotinib group, with treatment-related discontinuations occurring in 1.5% and 5.1% of patients, respectively. Five patients died because of TEAEs: 1 patient (0.7%) in the dirozalkib group (cardiac arrest) and 4 patients (2.9%) in the crizotinib group (cardiac arrest and respiratory arrest [*n* = 1], pneumonia [*n* = 2], and respiratory failure [*n* = 1]). Of note, the cardiac arrest and respiratory arrest events in the crizotinib group occurred in the same patient. None of the fatal TEAEs were considered treatment-related.

## Discussion

In this randomized phase III study, the novel ALK inhibitor dirozalkib significantly prolonged PFS compared with crizotinib, corresponding to a 53% reduction in the risk of disease progression or death. The ORR was comparable between the dirozalkib and crizotinib groups, whereas the median DoR was significantly longer with dirozalkib than with crizotinib (29.5 versus 11.1 months). Dirozalkib demonstrated superior efficacy to crizotinib in controlling intracranial disease, with a significantly higher intracranial response rate and more durable intracranial responses. Grade ≥3 TEAEs, serious AEs, and TEAEs leading to treatment discontinuation were less frequent with dirozalkib than with crizotinib.

Previous studies have demonstrated that second-generation ALK inhibitors, including alectinib, brigatinib, and ensartinib, markedly improve PFS compared with the first-generation ALK inhibitor crizotinib in treatment-naïve patients with advanced *ALK*-positive NSCLC, with median PFS ranging from 24.0 to 34.8 months and HRs ranging from 0.47 to 0.51 [19–22]. In the present study, the magnitude of the PFS benefit with dirozalkib versus crizotinib (HR = 0.47) was numerically comparable to that reported for these next-generation ALK inhibitors. However, no head-to-head comparisons with second- or third-generation ALK inhibitors were conducted; therefore, the relative efficacy of dirozalkib within the current treatment landscape remains to be established. The strong agreement between investigator and BICR assessments further underscores the reliability of these findings. Beyond PFS, although the ORR was only modestly higher with dirozalkib than with crizotinib (89.1% versus 81.9%), dirozalkib induced more durable systemic responses, with a median DoR of 29.5 months versus 11.1 months. This finding may be consistent with the potent pharmacodynamic activity of dirozalkib against multiple resistance-associated *ALK* mutations following crizotinib therapy. Although the survival data remain immature, dirozalkib appears to be a valuable first-line treatment option for patients with advanced *ALK*-positive NSCLC.

Among patients with measurable brain metastases at baseline, dirozalkib achieved a markedly higher intracranial ORR than crizotinib (92.3% versus 11.1%), while the advantages in the cumulative incidence of ICP and intracranial PFS further underscored the superior CNS disease control with dirozalkib compared with crizotinib. This benefit likely reflects the optimized CNS pharmacologic profile of dirozalkib, including enhanced BBB penetration and sustained activity against intracranial tumor clones that exhibit resistance to first-generation ALK inhibition. Supporting these clinical findings, preclinical evaluation in the H2228-luc human lung cancer intracranial xenograft model demonstrated that dirozalkib markedly inhibited intracranial tumor growth and prolonged survival compared with vehicle controls [18]. Notably, brain metastases were identified in approximately 21.1% of patients at baseline, whereas prior brain radiotherapy was reported in approximately 1.5% of patients in the present study. These characteristics were similar to those reported in previous studies of second-generation ALK inhibitors, in which baseline brain metastases were documented in approximately 26% to 42% of patients, although the proportion of patients with prior radiotherapy in the present study was lower than the previously reported range of 4.9% to 40% [9,11,23]. Considering the association of prior brain radiotherapy with improved intracranial disease control and survival outcomes [24,25], potential confounding due to differences in patient characteristics should be acknowledged when comparing CNS outcomes across studies. Collectively, these results demonstrate the potent intracranial efficacy of dirozalkib and reinforce the relevance of CNS penetration as a distinguishing therapeutic advantage over crizotinib.

The safety profile of dirozalkib in this study was consistent with that reported in the prior phase I study [18], with no new safety concerns identified. Although patients receiving dirozalkib had a longer median treatment duration than those receiving crizotinib, grade ≥3 TEAEs, serious AEs, and TEAEs resulting in dose interruption or reduction were less frequent with dirozalkib than with crizotinib. Discontinuation due to treatment-related AEs was rare, occurring in only 1.5% of dirozalkib-treated participants, highlighting the favorable tolerability of dirozalkib during extended treatment. The predominant TEAEs in the dirozalkib group were gastrointestinal disorders (including diarrhea, nausea, and vomiting) and elevated aminotransferase levels, most of which were grade 1 or 2 and were readily managed with supportive care or temporary dose adjustments. Diarrhea, the most frequently reported AE, typically occurred early in treatment and was transient (median duration, approximately 2 d) and mostly mild. Grade 3 diarrhea occurred in 19.0% of patients, with no grade 4 events reported, and rarely led to treatment discontinuation. Hepatotoxicity, a common feature of ALK inhibitors [26,27], manifested as increased alanine and aspartate aminotransferase levels in 62.0% and 59.1% of dirozalkib-treated patients, respectively. Grade ≥3 transaminase elevations were infrequent in both groups (5.8% with dirozalkib versus 5.1% with crizotinib) and required fewer dose modifications with dirozalkib than with crizotinib. Hyperglycemia occurred more frequently with dirozalkib (33.6%), typically lasting approximately 30 d, whereas grade 3 to 4 treatment-related hyperglycemia was rare. These findings are consistent with the safety profiles reported for other second-generation ALK inhibitors, which have shown comparable or higher incidences of hepatic and metabolic adverse effects [28].

Several limitations should be acknowledged. First, OS data remain immature, and the availability of effective subsequent therapies, including next-generation ALK inhibitors, may attenuate differences in OS between treatment groups, precluding definitive conclusions regarding long-term survival benefit. Thus, longer follow-up and future OS analyses will be essential to fully characterize the clinical benefit of dirozalkib. However, the observed magnitude of the PFS advantage suggests that a future OS benefit is plausible. Notably, although the primary end point was investigator-assessed PFS, which may be subject to assessment bias, the concordant BICR-assessed PFS findings support the reliability of the PFS results. In addition, the exclusive enrollment of patients at Chinese centers limits the generalizability of the findings to other populations, underscoring the need for global validation in future studies. The mITT analysis was conducted post hoc based on the regulatory approval status of the NGS assays used for *ALK* testing. This nonprespecified population may introduce selection bias. The analysis of intracranial efficacy was based on a small subset of patients with measurable brain metastases at baseline, limiting the statistical robustness and generalizability of these findings. Finally, although the statistical analyses were not independently verified by an external party, they adhered to the prespecified statistical analysis plan and were conducted under rigorous internal quality-control procedures in accordance with regulatory requirements.

## Conclusions

In summary, this phase III study demonstrated the superiority of dirozalkib over crizotinib in terms of PFS, with a manageable safety profile, in patients with advanced *ALK*-positive NSCLC who had not previously received ALK inhibitor therapy. These findings support dirozalkib as a promising first-line treatment option for patients with advanced *ALK*-positive NSCLC.

## Ethical Approval

This protocol was approved by the ethics committee at the leading center of Jilin Province Cancer Hospital (202111-106-07) and each participating site, and all patients provided written informed consent before enrollment.

## Data Availability

We confirm that the data supporting the findings of this study are available within the article and its Supplementary Materials.

## References

[1] Leiter A, Veluswamy RR, Wisnivesky JP. The global burden of lung cancer: Current status and future trends. Nat Rev Clin Oncol. 2023;20(9):624–639. 10.1038/s41571-023-00798-3 37479810

[2] Bowen Jones S, Chan C, Filippi AR, Harada K, Louie AV, Lindsay CR, Nadal E, Munoz Schuffenegger P, Woolf D, Faivre-Finn C, et al. Emerging role of targeted therapies combined with radiotherapy in inoperable stages I to III NSCLC: A review from the IASLC ART Subcommittee. J Thorac Oncol. 2025;20(8):1018–1031. 10.1016/j.jtho.2025.05.004 40350081

[3] Araghi M, Mannani R, Heidarnejad Maleki A, Hamidi A, Rostami S, Safa SH, Faramarzi F, Khorasani S, Alimohammadi M, Tahmasebi S, et al. Recent advances in non-small cell lung cancer targeted therapy; an update review. Cancer Cell Int. 2023;23(1):162. 10.1186/s12935-023-02990-y PMC10416536 37568193

[4] Vijayvergia N, Mehra R. Clinical challenges in targeting anaplastic lymphoma kinase in advanced non-small cell lung cancer. Cancer Chemother Pharmacol. 2014;74(3):437–446. 10.1007/s00280-014-2517-6 PMC5627646 25135623

[5] Qin A, Gadgeel S. The current landscape of anaplastic lymphoma kinase (ALK) in non-small cell lung cancer: Emerging treatment paradigms and future directions. Target Oncol. 2017;12(6):709–718. 10.1007/s11523-017-0526-1 PMC6000827 28856564

[6] Long M, Peng S, Wen Q, Yin Z, Zhang X, Tan H, Xu Y, Wu Y. New advances in understanding the mechanisms and treatment challenges of ALK-targeted therapy resistance in lung cancer. Cancer Drug Resist. 2025;8:43. 10.20517/cdr.2025.122 PMC12378566 40873540

[7] Gibson AJW, Box A, Dean ML, Elegbede AA, Hao D, Sangha R, Bebb DG. Retrospective real-world outcomes for patients with ALK-rearranged lung cancer receiving ALK receptor tyrosine kinase inhibitors. JTO Clin Res Rep. 2021;2(4): Article 100157. 10.1016/j.jtocrr.2021.100157 PMC8474209 34590010

[8] Sullivan I, Planchard D. ALK inhibitors in non-small cell lung cancer: The latest evidence and developments. Ther Adv Med Oncol. 2016;8(1):32–47. 10.1177/1758834015617355 PMC4699265 26753004

[9] Solomon BJ, Liu G, Felip E, Mok TSK, Soo RA, Mazieres J, Shaw AT, de Marinis F, Goto Y, Wu Y-L, et al. Lorlatinib vs crizotinib in treatment-naïve patients with advanced ALK+ non-small cell lung cancer: 5-year progression-free survival and safety from the CROWN study. J Clin Oncol. 2024;42(17_suppl): Article LBA8503.

[10] Griesinger F, Roeper J, Pöttgen C, Willborn KC, Eberhardt WEE. Brain metastases in ALK-positive NSCLC - time to adjust current treatment algorithms. Oncotarget. 2018;9(80):35181–35194. 10.18632/oncotarget.26073 PMC6205553 30416687

[11] Nelson TA, Wang N. Targeting lung cancer brain metastases: A narrative review of emerging insights for anaplastic lymphoma kinase (ALK)-positive disease. Transl Lung Cancer Res. 2023;12(2):379–392. 10.21037/tlcr-22-638 PMC9989815 36895918

[12] Rangachari D, Yamaguchi N, VanderLaan PA, Folch E, Mahadevan A, Floyd SR, Uhlmann EJ, Wong ET, Dahlberg SE, Huberman MS, et al. Brain metastases in patients with EGFR-mutated or ALK-rearranged non-small-cell lung cancers. Lung Cancer. 2015;88(1):108–111. 10.1016/j.lungcan.2015.01.020 PMC4355240 25682925

[13] Izadi N, Solár P, Hašanová K, Zamani A, Akbar MS, Mrázová K, Bartošík M, Kazda T, Hrstka R, Joukal M. Breaking boundaries: Role of the brain barriers in metastatic process. Fluids Barriers CNS. 2025;22(1):3. 10.1186/s12987-025-00618-z PMC11708195 39780275

[14] Rijmers J, Lebre MC, Beijnen JH, Schinkel AH. Resistance mutations and the blood–brain barrier: Key challenges in targeted treatment of brain metastatic non-small cell lung cancer. Acta Pharm Sin B. 2025;15(8):3833–3851. 10.1016/j.apsb.2025.06.002 PMC12399206 40893689

[15] Yang Y-L, Xiang Z-J, Yang J-H, Wang W-J, Xiang R-L. Effect of alectinib versus crizotinib on progression-free survival, central nervous system efficacy and adverse events in ALK-positive non-small cell lung cancer: A systematic review and meta-analysis. Ann Palliat Med. 2020;9(4):1782–1796. 10.21037/apm-19-643 32527124

[16] Yoshida T, Oya Y, Tanaka K, Shimizu J, Horio Y, Kuroda H, Sakao Y, Hida T, Yatabe Y. Clinical impact of crizotinib on central nervous system progression in ALK-positive non-small lung cancer. Lung Cancer. 2016;97:43–47. 10.1016/j.lungcan.2016.04.006 27237026

[17] Ni J, Li G, Yang X, Chu L, Wang J, Li Y, Zou L, Li Y, Xie C, Zhu Z. Optimal timing and clinical value of radiotherapy in advanced ALK-rearranged non-small cell lung cancer with or without baseline brain metastases: Implications from pattern of failure analyses. Radiat Oncol. 2019;14(1):44. 10.1186/s13014-019-1240-1 PMC6417092 30866974

[18] Ai X, Guo R, Zhang L, Fang J, Zhang Y, Chen J, Zhao J, Ye F, Wang S, Shi M, et al. Preclinical characterization and first-in-human, phase I trial of the novel ALK inhibitor dirozalkib in advanced non-small cell lung cancer. Eur J Cancer. 2026;235: Article 116221. 10.1016/j.ejca.2026.116221 41539180

[19] Horn L, Wang Z, Wu G, Poddubskaya E, Mok T, Reck M, Wakelee H, Chiappori AA, Lee DH, Breder V, et al. Ensartinib vs crizotinib for patients with anaplastic lymphoma kinase-positive non-small cell lung cancer: A randomized clinical trial. JAMA Oncol. 2021;7(11):1617–1625. 10.1001/jamaoncol.2021.3523 PMC8414368 34473194

[20] Camidge DR, Kim HR, Ahn M-J, Yang JCH, Han J-Y, Hochmair MJ, Lee KH, Delmonte A, Garcia Campelo MR, Kim D-W, et al. Brigatinib versus crizotinib in advanced ALK inhibitor-naive ALK-positive non-small cell lung cancer: Second interim analysis of the phase III ALTA-1L trial. J Clin Oncol. 2020;38(31):3592–3603. 10.1200/JCO.20.00505 PMC7605398 32780660

[21] Mok T, Camidge DR, Gadgeel SM, Rosell R, Dziadziuszko R, Kim D-W, Perol M, Ou S-HI, Ahn JS, Shaw AT, et al. Updated overall survival and final progression-free survival data for patients with treatment-naive advanced ALK-positive non-small-cell lung cancer in the ALEX study. Ann Oncol. 2020;31(8):1056–1064. 10.1016/j.annonc.2020.04.478 32418886

[22] Peters S, Camidge DR, Shaw AT, Gadgeel S, Ahn JS, Kim D-W, Ou S-HI, Pérol M, Dziadziuszko R, Rosell R, et al. Alectinib versus crizotinib in untreated ALK-positive non–small-cell lung cancer. N Engl J Med. 2017;377(9):829–838. 10.1056/NEJMoa1704795 28586279

[23] Solomon BJ, Bauer TM, Ou S-HI, Liu G, Hayashi H, Bearz A, Penkov K, Wu Y-L, Arrieta O, Jassem J, et al. Post hoc analysis of lorlatinib intracranial efficacy and safety in patients with ALK-positive advanced non-small-cell lung cancer from the phase III CROWN study. J Clin Oncol. 2022;40(31):3593–3602. 10.1200/JCO.21.02278 PMC9622589 35605188

[24] Wang W, Sun X, Hui Z. Treatment optimization for brain metastasis from anaplastic lymphoma kinase rearrangement non-small-cell lung cancer. Oncol Res Treat. 2019;42(11):599–606. 10.1159/000502755 PMC6921697 31527380

[25] Bauman JR, Liu G, Preeshagul I, Liu SV, Melosky B, Abrahami D, Li B, Thomaidou D, Duncan K, Krulewicz S, et al. Real-world treatment sequencing and effectiveness of second- and third-generation ALK tyrosine kinase inhibitors for ALK-positive advanced non-small cell lung cancer. Lung Cancer. 2024;195: Article 107919. 10.1016/j.lungcan.2024.107919 PMC12754696 39197358

[26] Juncu SS, Trifan AV, Minea H, Avram R-I, Cojocariu C, Sîngeap A-M. From spotlight to shadow: ALK inhibitor-induced acute liver failure in a patient with non-small cell lung cancer. Arch Clin Cases. 2023;10(4):160–163. 10.22551/2023.41.1004.10266 PMC10660242 38026106

[27] Zhao W, Jin X, Li Y, Peng H, Jiang J. Hepatotoxicity of ALK/ROS1 tyrosine kinase inhibitors in non-small cell lung cancer patients: A pharmacovigilance study based on signal mining and analysis of the FDA adverse event reporting system database. Sci Prog. 2025;108(4): Article 368504251387821. 10.1177/00368504251387821 PMC12536124 41091852

[28] Luo Y, Zhang Z, Guo X, Tang X, Li S, Gong G, Gao S, Zhang Y, Lin S. Comparative safety of anaplastic lymphoma kinase tyrosine kinase inhibitors in advanced anaplastic lymphoma kinase-mutated non-small cell lung cancer: Systematic review and network meta-analysis. Lung Cancer. 2023;184: Article 107319. 10.1016/j.lungcan.2023.107319 37597303

