# Supplementary material for: Dirozalkib versus Crizotinib in Advanced Anaplastic Lymphoma Kinase Inhibitor-Naïve ALK-Positive Non-Small-Cell Lung Cancer: A Multicenter, Randomized Phase III Trial
Source: Cancer Commun (Lond). 2026 Nov 10;46:0068. doi: 10.34133/cancomm.0068 (PMC13748627; doi:10.34133/cancomm.0068)
Supplement: Supplementary file 1 [file cancomm.0068.f1.zip › CANCOMM-D-26-00211-Supplementary Data S1.pdf]

Clinical Study Protocol

**Study Title:**	A Phase 3, Multicenter, Randomized, Open-label Clinical Study Comparing the Efficacy and Safety of XZP-3621 Tablets versus Crizotinib Capsules in Patients with ALK+ Advanced Non-Small Cell Lung Cancer
**Protocol Number:**	XZP-3621-3001
**Version Number:**	V2.0
**Version Date:**	December 19, 2022
**Product Name:**	XZP-3621 Tablets
**Study Phase:**	Phase 3
**Sponsor:**	Shandong Xuanzhu Pharma Co., Ltd.
**Principal Investigator:**	Professor Cheng Ying<br>Institution: Jilin Cancer Hospital

Confidentiality Statement

The information contained in this document is confidential and proprietary to Shandong Xuanzhu Pharma Co., Ltd. Confidential information in the document is for guidance of this clinical study only. This protocol and the information contained herein shall not, without the Sponsor's written consent, be disclosed to any individual or entity not directly involved in the conduct of this clinical study, or used for any other purpose.

Sponsor Signature Page

Study Title: A Phase 3, Multicenter, Randomized, Open-label Clinical Study Comparing the Efficacy and Safety of XZP-3621 Tablets versus Crizotinib Capsules in Patients with ALK+ Advanced Non-Small Cell Lung Cancer

Protocol Number: XZP-3621-3001

Version Number: V2.0/December 19, 2022

Sponsor's Declaration:

We will earnestly fulfill the responsibilities of the sponsor in accordance with the provisions of China’s Good Clinical Practice (GCP), and be responsible for sponsoring, submitting applying application, organizing, funding, and monitoring this clinical study. In accordance with the provisions of relevant national laws and regulations, we will provide active treatment to subjects with serious adverse events during the clinical study process and bear the corresponding treatment costs. In addition, we will provide reasonable economic compensation for damages from serious adverse reactions clearly caused by the investigational medicinal product.

I have participated in the development and discussion of this clinical study protocol and agree with the contents of the protocol. I have clearly understood the sponsor's responsibilities regarding this project's study protocol and agree to conduct the clinical study in accordance with the provisions of this protocol and all applicable laws and regulations.

Sponsor: Shandong Xuanzhu Pharma Co., Ltd.

Signature of Sponsor Representative:

Signature Date:

Principal Investigator Signature Page

Study Title: A Phase 3, Multicenter, Randomized, Open-label Clinical Study Comparing the Efficacy and Safety of XZP-3621 Tablets versus Crizotinib Capsules in Patients with ALK+ Advanced Non-Small Cell Lung Cancer

Protocol Number: XZP-3621-3001

Version Number: V2.0/December 19, 2022

Principal Investigator's Declaration:

This protocol is a confidential document of Shandong Xuanzhu Pharma Co., Ltd. and shall only be disseminated on a confidential basis. I confirm that I have completely read this protocol, understand its contents, and will carry out my work in accordance with this protocol. In addition, I will conduct my work in accordance with the ethical principles of the Declaration of Helsinki, Good Clinical Practice guidelines, and applicable laws and regulations. By signing this document, I agree that: without the prior written permission of Shandong Xuanzhu Pharma Co., Ltd., it is prohibited to disclose or reveal the non-public information contained herein.

I have read the entire contents of this protocol and agree to follow this protocol in the study.

Research Institution: Jilin Cancer Hospital

Signature of Principal Investigator:

Signature Date:

Protocol Synopsis

**Sponsor**:	Shandong Xuanzhu Pharma Co., Ltd.
**Investigational Medicinal Product:**	XZP-3621 Tablets
**Active Ingredient:**	XZP-3621
**Study Title:**	A Phase 3, Multicenter, Randomized, Open-label Clinical Study Comparing the Efficacy and Safety of XZP-3621 Tablets versus Crizotinib Capsules in Patients with ALK+ Advanced Non-Small Cell Lung Cancer
**Protocol Number:**	XZP-3621-3001
**Principal Investigator:**	Professor Cheng Ying
**Leading Site:**	Jilin Cancer Hospital
**Number of Study Sites:**	Approximately 50-60 centers
**Study Phase:**	Phase 3
**Study Population:**	ALK+ advanced NSCLC, patients previously treated with up to one chemotherapy regimen and not treated with any ALK-TKI.

Study Objectives:

Primary Objective:

- To compare the efficacy of XZP-3621 Tablets versus Crizotinib Capsules in patients with ALK+ advanced non-small cell lung cancer (NSCLC) (investigator-assessed PFS).

Secondary Objectives:

- To compare the efficacy of XZP-3621 Tablets versus Crizotinib Capsules in patients with ALK+ advanced NSCLC (BICR-assessed PFS; ORR, DoR, DCR, OS, OSR, IC-ORR, and IC-DoR assessed by BICR and investigators, respectively);
- To compare the safety and tolerability of XZP-3621 Tablets versus Crizotinib Capsules in patients with ALK+ advanced NSCLC;
- To evaluate the population pharmacokinetic characteristics of XZP-3621 in patients with ALK+ advanced NSCLC.

Exploratory Objectives:

- To compare disease/symptom-related patient-reported outcomes (PRO) and health-related quality of life (HRQOL) scores after treatment with XZP-3621 Tablets versus Crizotinib Capsules in patients with ALK+ advanced NSCLC;
- To compare the health status of subjects with ALK+ advanced NSCLC treated with XZP-3621 Tablets versus Crizotinib Capsules.

Study Endpoints:

Primary Endpoint:

- Investigator-assessed PFS (RECIST v1.1 criteria).

Secondary Endpoints:

- PFS assessed by Blinded Independent Central Review (BICR) (RECIST v1.1 criteria);
- ORR, DoR, DCR, OS, and OSR assessed by BICR and investigators, respectively;
- Objective response rate (IC-ORR) and duration of response (IC-DoR) in brain metastasis patients with measurable lesions assessed by BICR and investigators, respectively (RECIST v1.1 criteria and RANO-BM criteria);

- Safety: AE evaluation (assessed per CTCAE v5.0 criteria); physical examination and vital signs; laboratory tests (hematology, blood chemistry, coagulation function, and urinalysis); 12-lead ECG and echocardiography;
- Population pharmacokinetic characteristics of XZP-3621 in patients with ALK+ advanced NSCLC.

Exploratory Endpoints:

- Time to Deterioration (TTD), defined as the time from randomization to first deterioration in EORTC QLQ-C30 scale score.
- Mean scores and mean changes from baseline in the European Organisation for Research and Treatment of Cancer Quality of Life Core Questionnaire (EORTC QLQ-C30) (Appendix 8).
- Mean scores and mean changes from baseline in the European Organisation for Research and Treatment of Cancer Lung Cancer: a modular supplement to the EORTC Core Quality of Life Questionnaire (EORTC QLQ-LC13) (Appendix 9).
- Health Utility Index of the European Quality of Life 5-Dimension 5-Level Scale Questionnaire (EQ-5D-5L) (Appendix 10).

Study Design:

This study is a Phase 3, multicenter, randomized, open-label, parallel-controlled clinical study. The study plans to enroll 268 patients with ALK+ advanced NSCLC who had received only one chemotherapy regimen or no prior systemic antitumor therapy. Patients will be randomized 1:1 to either the test group (134 patients) or control group (134 patients) to compare the efficacy and safety of XZP-3621 Tablets versus Crizotinib Capsules in treating patients with ALK+ advanced NSCLC.

The entire study consists of a screening period, a treatment period, a safety follow-up period, and a survival follow-up period (see study flowchart).

Test group dosing method: XZP-3621 Tablets, 500 mg once daily, administered with meals or within 30 minutes after meals, continuously administered with 4-week treatment cycles.

Control group dosing method: Crizotinib Capsules, 250 mg twice daily, administered with or without food, continuously administered with 4-week treatment cycles.

Stratification factors: 1. Brain metastasis (Yes, No); 2. Chemotherapy (Yes, No).

Study Flowchart:

Screening Period (28 Days)

- ALK+ advanced NSCLC, patients previously treated with up to one chemotherapy regimen and not treated with any ALK-TKI.;
- ECOG performance status score 0-2;
- At least one extracranial measurable target lesion meeting RECIST v1.1 criteria.

Randomized in a 1:1 ratio (**randomization**)

Stratification

- Brain metastasis (Yes, No)
- Chemotherapy (Yes, No)

Test Group
XZP-3621 Tablets 500 mg QD
Continuous administration
Every 4 weeks constitutes 1 treatment cycle
Until investigator- and BICR-assessed disease progression or intolerable toxicity occurs.

Control Group
Crizotinib Capsules 250 mg BID
Continuous administration
Every 4 weeks constitutes 1 treatment cycle
Until investigator- and BICR-assessed disease progression or intolerable toxicity occurs.

Follow-Up Period

- End of Treatment Visit: Within 14 days after the end of treatment (EOT);
- Safety Follow-up: 30 days ± 3 days after the last dose;
- Survival Follow-up: Every 12 weeks ± 7 days after the last dose.

Tumor Assessment
Every 8 weeks during the first 64 weeks of treatment; subsequently every 12 weeks. Assessed per RECIST v1.1 criteria; for brain metastasis patients, assessed per RANO-BM criteria.

Treatment continues until subjects experience investigator- and BICR-assessed disease progression, intolerable toxicity, withdrawal of consent, death, investigator determination of unsuitability for continued treatment, or subject decision to discontinue study treatment, whichever occurs first.

During the study, if isolated asymptomatic central nervous system progression (such as new central nervous system oligometastases) occurs, local treatment (such as stereotactic radiotherapy or surgery) should be administered, followed by continued XZP-3621 (test group) or Crizotinib (control group) treatment until systemic disease progression and/or symptomatic central nervous system progression. For non-isolated symptomatic central nervous system progression, post-progression continuation of study treatment is determined by the investigator based on whether patients may continue benefiting from respective therapies.

Patients who discontinue treatment before disease progression (e.g., due to intolerable toxicity) will continue tumor assessment follow-up as scheduled until disease progression or initiation of new antitumor therapy.

**Planned Number of Subjects:**	Planned total enrollment: 268 subjects<br>**Test Group**: Planned to enroll 134 subjects;<br>**Control Group:** Planned to enroll 134 subjects.
**Main Inclusion/Exclusion Criteria:**	**Inclusion Criteria:**<br>Subjects must meet all of the following inclusion criteria to be eligible

	for this study:<br>1. Male or female subjects aged 18-75 years (inclusive);<br>2. Histologically or cytologically confirmed stage IV NSCLC or stage IIIB/IIIC NSCLC that is not amenable to radical radiotherapy (according to the 8th edition of the International Association for the Study of Lung Cancer staging) with ALK+ status confirmed by nationally approved detection methods (Ventana D5F3 IHC, FISH, PCR, or NGS). Subjects without prior detection reports must provide tissue samples for confirmation of the ALK gene;<br>3. Subjects who have not previously received systemic treatment for NSCLC, including ALK-TKIs, immunotherapy, or biological therapies (cancer vaccines, cytokines, or growth factors controlling cancer), etc. (Patients who have progressed or developed intolerable toxicity after one prior chemotherapy regimen are eligible);<br>Note: A prior chemotherapy regimen refers to systemic chemotherapy administered after diagnosis of stage IV NSCLC or unresectable stage IIIB/IIIC NSCLC. Progression within 6 months of neoadjuvant/adjuvant chemotherapy is considered as one prior chemotherapy regimen.<br>4. ECOG performance status score 0-2 (Appendix 2);<br>5. Subjects must have at least one extracranial measurable target lesion meeting RECIST v1.1 criteria that has not been previously irradiated. Previously irradiated lesions may only be considered target lesions if definitive progression post-radiation is documented;<br>6. Subjects without brain metastases or with asymptomatic brain metastases are eligible, provided:<br>a) Untreated and not requiring corticosteroids currently, or<br>b) Prior to randomization, local treatment has been completed, and patients have fully recovered from the acute effects of radiotherapy or surgery, corticosteroid hormone therapy has been discontinued for at least 4 weeks in these patients with brain metastases and neurological symptoms are stable;<br>c) Subjects with leptomeningeal disorder (LMD) or carcinomatous meningitis (CM) visible on MRI, or positive CSF cytology at baseline must be excluded.<br>7. Investigator-assessed expected survival ≥12 weeks;<br>8. Adequate organ function at baseline evaluation with laboratory tests meeting:<br>a. **Hematology:** ANC ≥1.5×10^9/L; platelet count ≥90×10^9/L; haemoglobin ≥90 g/L;<br>b. **Liver Function:** Serum total bilirubin ≤1.5× the upper limit of normal (ULN); ALT/AST ≤2.5× ULN (≤ 5× ULN in subjects with metastases to liver);<br>c. **Renal Function:** Blood creatinine ≤1.5× ULN or Ccr ≥50 mL/min (≥0.835 mL/s by Cockcroft-Gault formula, Appendix 6);<br>d. Serum amylase (AMS) ≤1.0×ULN (subjects with amylase increased 1-2×ULN are eligible if no other signs or clinical evidence of pancreatic disorder are present);

	e. Glycosylated haemoglobin ≤7.0%;<br>9. All surgeries and prior radiotherapy (except palliative)/operations must be completed ≥4 weeks before randomization; Palliative radiation must be completed ≥1 week before randomization;<br>10. All non-hematologic toxicities related to other anti-tumor therapies must have recovered to ≤ Grade 2 (except alopecia);<br>11. All women of childbearing potential must have negative pregnancy test within 3 days before treatment. Male or female subjects of childbearing potential must agree to use effective contraception methods during the study and for 90 days after last dose, such as double-barrier contraception, condoms, oral or injection contraceptive drugs, intrauterine devices, etc.;<br>12. Capable and willing to provide written informed consent before undergoing any study-related procedures and to comply with the study protocol.<br>**Exclusion Criteria:**<br>Subjects with any of the following cannot be enrolled in this study:<br>1. Subjects with concurrent small cell lung cancer;<br>2. Uncontrolled malignant pleural/peritoneal effusion and/or pericardial effusion (uncontrolled refers to significant reaccumulation within one week after drainage);<br>3. Diagnosis of any other neoplasm malignant within 3 years before enrollment (except adequately treated and stable basal cell carcinoma or early gastrointestinal (GI) cancer removed endoscopically or carcinoma in situ of cervix);<br>4. History of organ transplant, hematopoietic stem cell or bone marrow transplant therapy;<br>5. Major surgery or significant trauma due to any reason within 4 weeks before randomization (major surgery is defined as procedures under general anesthesia or involving significant incisions);<br>6. Active hepatitis B (HBsAg positive and/or HBcAb positive with HBV-DNA >500 IU/mL) or hepatitis C (HCV antibody positive with HCV RNA positive);<br>7. Concurrent human immunodeficiency virus (HIV) infection;<br>8. Clinically active infection affecting subject safety, including active pulmonary tuberculosis;<br>9. Uncontrolled hyperglycemia, acute cholelithiasis, or patients predisposed to pancreatitis acute;<br>10. Investigator-assessed uncontrolled electrolyte disturbances (e.g., hypocalcemia, hypomagnesemia, hypokalaemia);<br>11. Subjects unable to swallow;<br>12. History of extensive diffuse/bilateral interstitial fibrosis, or known Grade 3/4 interstitial fibrosis, or current clinically significant active lung diseases including pulmonary infection, pneumonia allergic, interstitial pneumonia, other interstitial lung diseases, obliterative bronchiolitis, or current radiation pneumonitis requiring steroid treatment (excluding prior history of radiation pneumonitis);

	13. Clinically significant vascular (arterial/venous) and non-vascular cardiac diseases within 3 months before randomization, potentially including but not limited to:<br>a) Arterial disorders: cerebrovascular accident/stroke (including transient ischaemic attack), myocardial infarction, angina unstable;<br>b) Venous disorders: cerebral venous thrombosis, symptomatic pulmonary embolism;<br>c) Non-vascular cardiac disorders: cardiac failure congestive (New York Heart Association Classification ≥ II), II/III degree atrioventricular block (excluding those with pacemakers) or atrioventricular block with PR>220 ms; NCI CTCAE grade ≥2 persistent arrhythmia, uncontrolled atrial fibrillation of any grade, bradycardia <50 bpm (unless in athletes), QTc>470 msec on ECG, or congenital long QT syndrome;<br>d) Cardiac ultrasound indicates LVEF＜50%;<br>14. Requirement for concomitant use of QTc-prolonging drugs or medications inducing torsade de pointes ventricular tachycardia within 14 days before randomization or during treatment;<br>15. Participation in other clinical studies with treatment received within one month before randomization;<br>16. Uncontrolled hypertension (defined as resting blood pressure systolic ≥150 mmHg and/or blood pressure diastolic ≥95 mmHg);<br>17. Clinically significant gastrointestinal abnormalities including active colitis ulcerative, chronic diarrhoea due to malabsorption, Crohn's disease, and/or prior surgery affecting absorption;<br>18. Other severe and/or uncontrolled comorbidities that may affect safety or efficacy assessments as judged by the investigator;<br>19. Subjects currently receiving potent CYP3A4 inhibitors/inducers that cannot be discontinued within 1 week prior to prior to randomization (or within 5 half-lives of the drug, whichever is longer) and during the study period;<br>20. Subjects planning to undergo surgery, or whom the investigator considers may require surgery during the study period (affecting subject safety or efficacy evaluation);<br>21. Subjects with known allergies to Crizotinib Capsules or any component of XZP-3621 Tablets, or those with a history of severe allergies;<br>22. Female patients during pregnancy or lactation;<br>23. Subjects with psychiatric history, incapacitated persons, or persons with limited legal capacity;<br>24. History of alcoholism or drug abuse;<br>25. Other circumstances deemed by the investigator to be unsuitable for enrollment.
**Investigational Medicinal Product, Strengths, and Administration Method:**	Product Name: XZP-3621 Tablets<br>Strengths: 250 mg, 50 mg<br>Dosing Method: XZP-3621 Tablets, 500 mg once daily, administered with meals or within 30 minutes after meals, continuously administered with 4-week treatment cycles.

**Control Drug, Strengths, and Administration Method:**	Drug Name: Crizotinib Capsules<br>Drug Strengths: 250 mg, 200 mg<br>Dosing Method: Crizotinib Capsules, 250 mg twice daily, administered with or without food, continuously administered with 4-week treatment cycles.
**Study Period:**	1. **Screening Period:** 28 days;<br>2. **Treatment Period:** Each 4-week period constitutes one treatment cycle (bi-weekly visits during the first treatment cycle, followed by 4-week visits thereafter), continuing until disease progression as assessed by investigators and BICR, intolerable toxicity, withdrawal of consent or death, or until investigators determine the subject's condition is unsuitable for continued treatment or the subject decides to discontinue treatment (whichever occurs first).<br>3. **Safety Follow-up Period:** 30 days after the last dose;<br>4. **Survival Follow-up Period:** Survival follow-up will be conducted every 12 weeks after the last dose until death from any cause, loss to follow-up, withdrawal of consent, or study closure (whichever occurs first).

Statistical Methods:

Analysis Population:

- Full Analysis Set (FAS): Defined as all successfully screened and randomized subjects.
- Per-Protocol Set (PPS): A subset of subjects in the FAS, excluding those with major protocol deviations affecting outcomes. The list of subjects included in or excluded from PPS must be reviewed and confirmed by the sponsor and investigators before database lock.
- Safety Set (SS): Includes all randomized subjects who have received at least one dose of the investigational medicinal product. In the SS, subjects are analyzed according to the actual treatment received.

Sample Size Estimation:

The progression-free survival (PFS) is used as the primary efficacy endpoint in this study.

A parallel-controlled superiority study design is adopted, with the primary endpoint being investigator-assessed PFS. Subjects are randomized into the test group and control group in a 1:1 ratio. A median PFS of 11 months in the control group and 20 months in the test group is assumed, with a hazard ratio of 0.55. With a one-sided significance level of 0.025 ($\alpha=0.025$) and 95% power, assuming an enrollment duration of approximately 18 months and 20 months follow-up after the last subject randomization, an interim analysis will be conducted when 70% of PFS events are collected, using the Lan-DeMets alpha spending function approximating the O'Brien-Fleming boundary to control overall type I error. The total required PFS events are 147, with 201 total subjects. Considering a 15% dropout rate, this study requires enrollment of at least 238 subjects (119 in the test group and 119 in the control group). The number of PFS events required for the interim analysis is 103.

This study is conducted during the COVID-19 pandemic. Considering that the increased SARS-CoV-2 infection rate among lung cancer patients may lead to higher dropout rates, and the extended study duration (approximately 38 months) can also contribute to increased dropout rates, approximately 268 subjects (134 in the test group and 134 in the control group) with a 25% dropout rate will be enrolled to ensure the study proceeds as planned. The total number of required PFS events remains 147.

General Principles for Statistical Analysis:

Statistical analysis will be performed using SAS software version 9.4 or higher. All statistical tests will be two-sided with a 95% confidence interval (CI). Measurement data across treatment groups at each visit will be statistically described using numbers, means, standard deviations, quartiles, minimums, and maximums. Count data will be described using frequencies (percentages).

Primary Endpoint Analysis:

The primary endpoint of this study is investigator-assessed PFS. Survival rates will be estimated and survival curves will be plotted using Kaplan-Meier method, with 95% CI calculated. The survival functions of the two groups will be compared using the Log-Rank test, taking into account stratification factors. The impact of stratification factors on event endpoints will be considered, with between-group effects and their 95% CIs estimated. The hazard ratio for PFS between groups will be analyzed using COX proportional hazards model in the full analysis set, with brain metastasis and chemotherapy as influencing factors.

Primary endpoint analysis will be based on FAS and PPS, with FAS as the primary analysis set.

Secondary Endpoint Analysis:

Analysis methods for secondary endpoints (BICR-assessed PFS, investigator/BICR-assessed OS, DoR) are the same as those for investigator-assessed PFS. OSR at different timepoints will be estimated using Kaplan-Meier method, while the ORR, DCR, CBR, and their 95% CIs will be calculated using the Cochran-Mantel-Haenszel (CMH) test, considering the effects of stratification factors across the groups.

Continuous data will undergo descriptive statistical summarization including: mean, standard deviation, median, minimum, and maximum.

Exploratory Endpoint Analysis:

Time to deterioration (TTD) is defined as the time from randomization to first deterioration in EORTC QLQ-C30 scale score. The analysis method will be the same as that for investigator-assessed PFS, using the Kaplan-Meier method to estimate survival rates and plot survival curves, with 95% CIs estimated.

EORTC QLQ-C30 and QLQ-LC13 quality-of-life scores will be aggregated by visit and analyzed for changes from baseline. Domain scores will be calculated based on individual items, and the score results for each domain and the changes from baseline will be summarized and analyzed. A mixed-effects model for repeated measures will be used to compare the changes in quality of life scores from baseline between the two groups, with the model using change relative to baseline as the dependent variable, and group and baseline scores as covariates.

The EQ-5D 5L Health Index score will be computed using a weighted approach, with results summarized by visit and changes from baseline analyzed at each visit point. Domain scores will be calculated based on individual items, and the score results for different domains and the changes from baseline will be summarized and analyzed. A mixed-effects model for repeated measures will be used to compare the changes in quality of life scores from baseline between the two groups, with the model using change relative to baseline as the dependent variable, and group and baseline scores as covariates. Aggregate analysis will summarize frequency by category: 'Mobility', 'Self-care', 'Usual activities', 'Pain/discomfort', and 'Anxiety/depression'.

Safety Analysis:

Safety analysis will be based on the SS (actual treatment groups). Safety analysis will be limited to descriptive statistical summaries, including but not limited to:

- Adverse event
- Serious adverse event
- Laboratory test
- Vital signs, physical examination, electrocardiogram examination

Adverse events in this study will be evaluated according to NCI-CTCAE v5.0, with Kaplan-Meier method used for analyzing time to adverse event occurrence and event duration.

Interim Analysis:

This study is designed to include one planned interim analysis. The primary endpoint is investigator-assessed PFS. The interim analysis will be conducted when approximately 70% (about 103) of projected PFS events are collected. Interim analysis thresholds and efficacy boundaries for each stage are as follows:

Analysis Phase	Event Count	Z Boundary Value	One-Sided Nominal Significance Level
First Analysis	103	2.44	0.0074

Final Analysis	147	2.00	0.0228

Note:

1. The alpha-spending function will use Lan-DeMets (O'Brien-Fleming) method to control the overall type I error at one-sided 0.025;

2. The actual nominal significance level and boundaries for interim analysis will be determined based on the proportion of events observed at that time. The nominal significance level and boundaries for final analysis will be adjusted accordingly.

**Study Duration**	Subject enrollment is expected to be completed within approximately 18 months. Final PFS analysis will be performed when approximately 147 PFS events are observed.

Study Flowchart

Study Period	Screening Period	Treatment Period						End of Treatment Visit [x] (EOT)	Safety Visit [y]	Survival Visit [z]	
		Cycle 1		Cycle 2	Cycle 3	Cycle 4	Cycle n				
**Visit Schedule**	**D-28~D-1**	**C1D1[w]**	**C1D15**	**C2D1**	**C3D1**	**C4D1**	**CnD1**		**30 days after drug discontinuation**	**Efficacy Follow-up**	**Survival Follow-up**
**Visit Window**	**NA**	**NA**	**±3D**	**±3D**	**±3D**	**±3D**	**±3D**	**0-14D**	**±3D**	**±7D**	**±7D**
Informed Consent	X										
Inclusion/Exclusion Criteria	X	X									
Demographics [a]	X										
Non-tumor Medical History [b]	X										
Tumor Diagnosis and Treatment History [c]	X										
Randomization		X[aa]									
Vital Signs[d]	X	X	X	X	X	X	X	X			
Physical Examination [e]	X	X	X	X	X	X	X	X			
ECOG Score	X	X	X	X	X	X	X	X			
Glycosylated Haemoglobin Test (HbA1C) [f]	X					X	X	X			
Hepatitis B Five Items, HCV, HIV Testing [g]	X										
Blood Pregnancy Test [h]	X	X						X			

**Study Period**	**Screening Period**	**Treatment Period**						**End of Treatment Visit [x] (EOT)**	**Safety Visit [y]**	**Survival Visit [z]**	
		**Cycle 1**		**Cycle 2**	**Cycle 3**	**Cycle 4**	**Cycle n**				
**Visit Schedule**	**D-28~D-1**	**C1D1[w]**	**C1D15**	**C2D1**	**C3D1**	**C4D1**	**CnD1**		**30 days after drug discontinuation**	**Efficacy Follow-up**	**Survival Follow-up**
**Visit Window**	**NA**	**NA**	**±3D**	**±3D**	**±3D**	**±3D**	**±3D**	**0-14D**	**±3D**	**±7D**	**±7D**
Hematology [i,j]	**X**	**X**	**X**	**X**	**X**	**X**	**X**	**X**			
Blood Chemistry [i,k]	**X**	**X**	**X**	**X**	**X**	**X**	**X**	**X**			
Coagulation Function [i,l]	**X**	**X**		**X**	**X**	**X**	**X**	**X**			
Urinalysis [i,m]	**X**	**X**		**X**	**X**	**X**	**X**	**X**			
12-lead ECG [n]	**X**	**X**	**X**	**X**	**X**	**X**	**X**	**X**			
Echocardiography [o]	**X**							**X**			
PK Blood Collection [p]		**X**	**X**	**X**	**X**	**X**					
Tumor Imaging Examinations and Tumor Assessments (RECIST 1.1 criteria and RANO-BM criteria [q])	**X**				**X**		**X**	**X**		**X**	
Tumor Tissue Sample (ALK Mutation Testing) [r]	**X**										
Administration [s]		**X**	**X**	**X**	**X**	**X**	**X**				
Recording of AE [t]	**X**	**X**	**X**	**X**	**X**	**X**	**X**	**X**	**X**		

Study Period	Screening Period	Treatment Period						End of Treatment Visit [x] (EOT)	Safety Visit [y]	Survival Visit [z]	
		Cycle 1		Cycle 2	Cycle 3	Cycle 4	Cycle n				
Visit Schedule	D-28~D-1	C1D1[w]	C1D15	C2D1	C3D1	C4D1	CnD1		30 days after drug discontinuation	Efficacy Follow-up	Survival Follow-up
Visit Window	NA	NA	±3D	±3D	±3D	±3D	±3D	0-14D	±3D	±7D	±7D
Recording of Prior and Concomitant Therapies [u]	X	X	X	X	X	X	X	X	X		
Patient-Reported Outcomes (PRO) [v]		X		X	X	X	X	X			
Disease Progression and Survival Information											X
Subsequent Anti-tumor Therapy									X		X

Note: C = Cycle; CT = Computed Tomography; D = Day; ECOG = Eastern Cooperative Oncology Group; MRI = Magnetic Resonance Imaging; PK = Pharmacokinetics; AE = Adverse Event. Unless otherwise specified, the visit test windows should follow the windows in the flow chart.

a. Demographic Data: Gender, age, ethnicity, etc.;

b. Non-tumor medical history: past medical history, allergy history, present medical history;

c. Tumor diagnosis and treatment history:
   - Type of initial diagnosis tumor, date of initial diagnosis, initial diagnosis tumor stage (TNM staging; record pathological staging results if available), histology/cytology results, tumor stage at this visit (TNM staging);
   - All prior tumor treatments, including the type of treatment (surgery, radiotherapy, chemotherapy, immunotherapy, biotherapy, steroids, etc.), surgery date, name of the surgical procedure and extent of surgery, radiotherapy treatment method, radiation dose and start and end times, drugs or biological agents used, start and stop dates of treatment, best efficacy achieved by the treatment, and reason for stopping treatment;

d. Vital signs: It is recommended that subjects should have their respiratory rate, seated blood pressure and pulse, and body temperature measured after resting for at least 5 min. If the scheduled time for vital sign measurement coincides with the time for blood sample collection for laboratory tests, the vital signs should be measured before

the laboratory test sample collection or after resting for more than 10 min after blood collection.

e. Physical examination: The investigator will conduct physical examination according to the study procedure. The examination parameters include: body height (screening period only), weight, general condition, skin and mucous membranes, superficial lymph nodes throughout the body, head and its organs, neck, chest, abdomen, rectum, anus and external genitalia, spine and limbs, and nervous system.

f. Glycosylated haemoglobin (HbA1C): Performed at screening and EOT visit, and every 3 treatment cycles during treatment (e.g., C4D1, C7D1). At the EOT visit, there is no need to repeat the test if it has been re-tested within 1 month.

g. Hepatitis B Five Items, HCV, HIV Testing: HBsAg positive and/or HBcAb positive require additional HBV-DNA testing; HCV antibody positive requires additional HCV-RNA testing. During the study, the investigator may add HBV-DNA and HCV-RNA tests as needed.

h. Blood Pregnancy Test: All female subjects of childbearing potential will undergo serum pregnancy test during the screening period, on C1D1, and at end-of-treatment visit. If the pregnancy screening occurs within 3 days prior to the first dose, there is no need to repeat the test on C1D1.

Women with no childbearing potential: postmenopausal women (subjects should have natural amenorrhoea for at least 12 months); or have undergone oophorectomy bilateral; or have undergone hysterectomy or bilateral tubal ligation.

i. Hematology, blood chemistry, coagulation function, and urinalysis: If the screening test is completed within 7 days before administration, there is no need to repeat the test on C1D1.For subjects who have undergone the test within 7 days at the EOT visit, there is no need to repeat the test.

j. Hematology: white blood cell count (WBC), neutrophil count (NEUT), red blood cell count (RBC), neutrophil percentage (NEUTP), haemoglobin (HGB), lymphocyte count (LYMPHN), haematocrit (HCT), lymphocyte percentage (LYMPHP), platelet count (PLT), monocyte count (MONON), eosinophil count (EON), monocyte percentage (MONOP), eosinophil percentage (EOP), basophil percentage (BASOP), basophil count (BASON). If neutropenia (absolute neutrophil count $<1.0\times10^9$/L) or thrombocytopenia (platelet count $<50.0\times10^9$/L) occurs during medication, test is required at least twice a week until the toxicity is reduced to ≤ Grade 2.

k. Blood chemistry: Blood chemistry samples should be collected under fasting states. Test parameters include potassium (K), total protein (TP), sodium (Na), albumin (ALB), chlorine (Cl), total bilirubin (TBIL), magnesium (Mg), direct bilirubin (DBIL), calcium (Ca), alanine aminotransferase (ALT), creatinine (CREA), aspartate aminotransferase (AST), urea (UREA)/blood urea nitrogen (BUN), alkaline phosphatase (ALP), total cholesterol (TCHO), lactate dehydrogenase (LDH), triglycerides (TG), blood glucose (GLU), low density lipoprotein (LDL-C), serum lipase (LPS), high density lipoprotein (HDL-C), serum amylase (AMS), creatine kinase (CK), creatine kinase isoenzyme (CK-MB). If the patient has a history of diabetes mellitus or abnormal blood glucose is found during screening, it is recommended that the patient monitor blood glucose during the screening period. If ≥CTCAE Grade 3 hepatotoxicity or nephrotoxicity occurs during medication, re-test is required at least twice a week until the toxicity decreases to ≤ Grade 2.

l. Coagulation function: activated partial prothrombin time (APTT), international normalised ratio (INR), prothrombin time (PT), fibrinogen (FIB).

m. Urinalysis: white blood cells urine (WBC), red blood cells urine (RBC), protein urine (PRO), ketone bodies (KET), urine glucose (GLU), pH value.

n. 12-Lead ECG: Subjects need to undergo ECG examination in a resting state and remain supine but awake when collecting the electrocardiogram. Record HR, PR interval, RR interval, QRS duration, QT interval, QTc, QT interval, QT interval corrected for heart rate using Fridericia's formula [QTcF] (Appendix 3), and any abnormal value assessments. Each tracing should have at least 5 complete heartbeats and be recorded for at least 10 seconds. interval prolongation is defined as a single QTcF interval ≥500 msec or a single QTcF interval increase from baseline ≥60 msec. If QTcF prolongation occurs during the study, the use of the investigational product should be suspended, an electrocardiogram should be repeated, and (or if the investigator deems it necessary) a consultation with cardiologists should be requested. If the cardiologists confirm that the subject has a significant QTcF interval prolongation: the use of the IMP will be discontinued; the subject will undergo medical evaluation, appropriate treatment, and at least three ECG examinations per week until the QTcF interval is <480 msec; treatment may be resumed with the investigator’s consent. Throughout the study, the investigator may decide whether it is necessary to perform an additional 12-lead ECG examination outside the time points specified in the protocol based on the subject's clinical symptoms and signs.

o. Echocardiography: Performed at screening and EOT visits, with LVEF measurement required. If a subject's clinical symptoms/signs/ECG findings during the study indicate echocardiographic evaluation, the investigator may perform additional echocardiography as clinically indicated.

p. PK blood collection: Collected from subjects in the test group, C1D1: before drug administration on the same day, 4 h after drug administration on the same day, and C1D15, C2D1, C3D1, C4D1: before drug administration on the same day (patients are required to take the medication at the study site) for PK blood sample collection. PK blood collection is not required for subjects in the control group.

q. Tumor imaging examination and tumor assessment (RECIST1.1 criteria (Appendix 4) and RANO-BM criteria (Appendix 5)): Tumor lesion assessment during the screening period requires enhanced CT scans of the chest, abdomen, and pelvis, enhanced MRI of the head, and bone scan. Subjects with contrast media allergy may use plain CT, while maintaining consistent imaging methods throughout the study for each subject. CT and MRI examination results completed within 28 days prior to randomization are acceptable. Bone scan results within 3 months prior to randomization are acceptable. Subsequent visits should include a bone scan when clinically indicated (e.g., suspected metastases to bone or exacerbation). For diagnostic or staging needs, the investigator may decide whether to perform additional imaging examinations (e.g., PET/CT or bone scan) to detect other potential tumor lesions. The time window for imaging examinations during the treatment period is ±7 days. Imaging examinations will be performed every 8 weeks for the first 64 weeks of the treatment period, and every 12 weeks thereafter, with an imaging examination of all tumor lesions performed at the EOT visit (except for those who have imaging examinations of all tumor lesions within 4 weeks). Unscheduled imaging examinations may also be added if disease progression is suspected. After stopping investigational medicinal product therapy for any reason (other than death, disease progression, loss to follow-up, study termination, or withdrawal of informed consent), subjects will continue to undergo tumor assessments at the time points specified in the protocol during the follow-up period until disease progression or initiation of new anti-tumor therapy.

r. Tumor tissue sample (ALK mutation detection): If a previous ALK+ report cannot be provided during the screening period, a tissue specimen must be provided to confirm the ALK gene status.

s. Administration: Subjects in the test group will take the drug at the study site after completing various tests and examinations on the same day and PK blood collection before administration (-60 min).

t. Recording of AE: Any diseases, clinically significant symptoms/signs (including abnormal auxiliary examinations and laboratory test results) occurring from signed informed consent through 30 days post last dose - regardless of relationship to the IMP - should be recorded as AEs. Pre-existing medical conditions/signs/symptoms (including screening-detected laboratory/auxiliary examination abnormal results) without severity worsening or frequency increase during the study will be considered baseline medical history rather than AEs.

u. Recording of concomitant therapies: All medications (excluding IMP, including over-the-counter drugs, minerals, or vitamins) and non-drug therapies (including physical therapy and transfusion) used from 6 weeks prior to first dose of IMP through 30 days post last dose of IMP.

v. Patient-Reported Outcomes (PRO): Paper versions of the EORTC QLQ-C30, EORTC QLQ-LC13, and EQ-5D-5L questionnaires will be completed by all subjects at each visit before any examination. Investigators should ensure the completeness of all questionnaires. Assessments are required every 4 weeks until the end-of-treatment visit. If a subject has been assessed within 7 days prior to the end-of-treatment visit, PRO assessments do not need to be repeated at this visit. PRO assessments should adhere to the time window of the visit in question.

w. C1D1: Vital signs, physical examination, ECOG score, blood pregnancy, and 12-lead ECG results obtained within 3 days before the first dose are acceptable. Hematology, blood chemistry, coagulation function, and urinalysis results obtained within 7 days before the first dose are acceptable.

x. EOT visit: Conducted within 14 days after the investigator determines that a subject withdraws from study treatment. Subjects who discontinue treatment early should undergo tumor assessment at the EOT visit if they do not have tumor assessment results within 4 weeks.

y. Safety Visit: The study site will contact the subject by phone or recall subjects to the study site within 30 days after the last dose of investigational medicinal product for a safety follow-up visit to understand the resolution status of ongoing AEs or laboratory test abnormal changes, and to assess new AEs, SAEs, the use of new concomitant medications/subsequent anti-tumor therapies, or changes in previous concomitant medications that occurred from the last assessment to 30 days after the last dose. Any new events occurring during the safety follow-up period need to be recorded in the eCRF.

z. Survival Follow-up: The study site will contact subjects by phone or recall subjects to the study site every 12 weeks after the last dose for survival follow-up to understand the survival information and subsequent anti-tumor treatment. Survival follow-up will continue until subject death, loss to follow-up, or withdrawal of informed consent (whichever occurs first). After stopping investigational medicinal product therapy for any reason (other than death, disease progression, loss to follow-up, study termination, or withdrawal of informed consent), subjects will continue to undergo tumor assessments at the time points specified in the protocol during the follow-up period until disease progression or initiation of new anti-tumor therapy.

aa. Randomization: Randomization can be performed after verifying that the subjects meet all inclusion criteria and do not meet the exclusion criteria. Subjects should receive the first study treatment on the day of randomization as much as possible, but no later than 3 days after randomization.

Table of Contents

Sponsor Signature Page 2
Principal Investigator Signature Page 3
Protocol Synopsis 4
Study Flowchart 13
Table of Contents 19
List of Abbreviations 25
1. Introduction 28
1.1 Investigational Medicinal Product 28
1.1.1 Nomenclature 28
1.1.2 Physicochemical Properties 28
1.1.3 Mechanism of Action 28
1.2 Study Background 28
1.3 Preclinical Studies 29
1.3.1 Preclinical Pharmacodynamic Studies 29
1.3.2 Preclinical Pharmacokinetic Studies 30
1.3.3 Preclinical Toxicology Studies 31
1.4 Clinical Studies 33
1.4.1 Preliminary Analysis of Clinical Pharmacokinetic Data 33
1.4.2 Preliminary Safety Analysis 34
1.4.3 Preliminary Efficacy Analysis 35
1.5 Study Rationale 36
1.6 Study Risks/Benefits 36
1.6.1 Risk Analysis 36
1.6.2 Benefit Analysis 37
2. Study Objectives 38
2.1 Primary Objective 38
2.2 Secondary Objectives 38
2.3 Exploratory Objectives 38
3. Study Endpoints 39
3.1 Primary Endpoint 39
3.2 Secondary Endpoints 39
3.3 Exploratory Endpoints 39
4. Study Design 40
4.1 Study Design Overview 40
4.2 Independent Data Monitoring Committee (IDMC) 42

4.3 Interim Analysis 42
4.4 End of Study 42
4.5 Treatment after End of Study 42
5. Study Population 44
5.1 Inclusion Criteria 44
5.2 Exclusion Criteria 45
5.3 Other Eligibility Considerations 47
5.4 Study Screening and Randomization 47
5.4.1 Study Screening 47
5.4.2 Randomization 48
5.5 Handling Procedures for Inappropriately Enrolled Subjects 48
5.6 Subject Discontinuation of Study Treatment 48
5.7 Subject Withdrawal from the Study 50
6. Study Treatment 51
6.1 Investigational Product 51
6.1.1 Investigational Medicinal Product 51
6.1.2 Control Drug 51
6.2 Management of Investigational Medicinal Product 51
6.2.1 Drug Management 51
6.2.2 Supply, Packaging, and Labeling 52
6.3 Investigational Medicinal Product Dosage and Administration 52
6.3.1 Overdose or Missed Dose of Investigational Medicinal Product 52
6.4 Dose Adjustment 53
6.4.1 Dose Adjustment Principles of XZP-3621 Tablets 53
6.4.2 Dosage Adjustment Principles of Crizotinib Capsule 53
6.5 Concomitant Therapies 54
6.5.1 Prohibited Concomitant Therapies 55
6.5.2 Permitted Concomitant Therapies 57
6.5.3 Concomitant Therapies to Be Used with Caution 58
6.6 Treatment Compliance 58
7. Study Procedure 59
7.1 Visit Procedures 59
7.1.1 Screening Visit (D-28~ D-1) 59
7.1.2 Treatment Period Visits (C1-Cn) 60
7.1.3 End-of-Treatment (EOT) Visit 63
7.1.4 Safety Follow-up (30 days after the last dose) 64
7.1.5 Survival Follow-up (every 12 weeks after the last dose) 64
7.2 PK Blood Sampling Procedure 65

7.3 Sample Collection, Storage and Destruction 65
8. Study Assessment 66
8.1 Safety Assessment 66
8.1.1 Vital Signs 66
8.1.2 Physical Examination 66
8.1.3 Echocardiography 66
8.1.4 Electrocardiogram 67
8.1.5 Laboratory Safety Test 67
8.2 Efficacy Assessment 68
8.3 Pharmacokinetic Assessment 69
8.4 Patient-Reported Outcomes 69
9. Adverse Event 70
9.1 Definition of Adverse Event 70
9.2 Serious Adverse Event 71
9.2.1 Definition of Serious Adverse Event 71
9.2.2 Definition of Suspected Unexpected Serious Adverse Reaction 72
9.2.3 Disease Progression and New Primary Tumor 72
9.2.4 Abnormal Liver Function Test 72
9.3 Collection and Recording of AEs 73
9.3.1 Severity of AEs 74
9.3.2 Causality Assessment 74
9.4 Reporting of SAEs 75
9.5 Follow-up of AEs 76
9.6 Overdose 76
9.7 Pregnancy 76
9.7.1 Pregnancy Test 77
9.7.2 Time of Pregnancy Information Collection 77
9.7.3 Management of Occurring Pregnancy 77
10. Statistical Methods 78
10.1 General Principles 78
10.2 Sample Size Determination 78
10.3 Analysis Population 79
10.3.1 Full Analysis Set (FAS) 79
10.3.2 Safety Set (SS) 79
10.3.3 Per-Protocol Set (PPS) 79
10.4 Statistical Analysis 79
10.4.1 Primary Endpoint Analysis 79
10.4.2 Secondary Endpoint Analysis 79

10.4.3 Subject Population Analysis 80
10.4.4 Efficacy Analysis 80
10.5 Safety Data Analysis 81
10.6 Exploratory Analysis 82
10.7 Subgroup Analysis 82
11. Quality Assurance and Quality Control 84
11.1 Clinical Monitoring 84
11.2 Quality Assurance Audit 84
12. Data Management 85
13. Ethical Considerations 86
13.1 Ethics Committee (EC) 86
13.2 Ethical Conduct in the Study 86
13.3 Subject Information and Informed Consent 86
13.4 Subject Data Protection 87
14. Study Management 88
14.1 Management Structure 88
14.2 Overall Study Premature Termination and Study Site Closure 88
14.3 Study Document Retention 88
14.4 Access to Original Data/Documents 88
14.5 Investigator Responsibilities 89
14.6 Amendment to Study Protocol 89
14.7 Protocol Deviation 89
14.8 Publication Policy 90
14.9 Financial and Insurance Information 90
15. References 91
16. Appendices 92
16.1 Appendix 1: Laboratory Safety Test 92
16.2 Appendix 2: ECOG Performance Status 94
16.3 Appendix 3: Fridericia's Formula 95
16.4 Appendix 4: Response Evaluation Criteria in Solid Tumors 1.1 (RECIST v1.1) 96
16.4.1 Inclusion Criteria 96
16.4.2 Definitions 96
16.4.3 Measurement Methods 96
16.4.4 Tumor Response Evaluation 97
16.5 Appendix 5: RANO-BM 100
16.6 Appendix 6: Cockcroft-Gault Formula 103
16.7 Appendix 7: Best Supportive Care 104
16.8 Appendix 8: Quality of Life Questionnaire (EORTC QLQ-C30) 107

16.9 Appendix 9: Quality of Life Score (EORTC QLQ-LC13) 109
16.10 Appendix 10: Health Index Questionnaire (EQ-5D 5L) 110
16.11 Appendix 11: Investigator Signature Page 112

List of Tables

Table 6-1 Some Potent CYP3A4 Inhibitors and Inducers 55
Table 6-2 Concomitant Medications That May Cause QT Interval Prolongation 55
Table 7-1 PK Blood Sampling Time Points and Blood Volume 65
Table 16-1 Hepatitis B Five Items, HCV, HIV 92
Table 16-2 Hepatitis B Virus Deoxyribonucleic Acid 92
Table 16-3 Hematology Parameters 92
Table 16-4 Blood Chemistry Parameters 93
Table 16-5 Coagulation Parameters 93
Table 16-6 Glycosylated Haemoglobin 93
Table 16-7 Blood Human Chorionic Gonadotropin 93
Table 16-8 Urinalysis Parameters 93
Table 16-9 ECOG Performance Status Scale 94

List of Abbreviations

Abbreviations	Full Term
AE	Adverse Event
ALP	Alkaline Phosphatase
ALT	Alanine Aminotransferase, also known as Glutamic-Pyruvic Transaminase
APTT	Activated Partial Thromboplastin Time
AST	Aspartate Aminotransferase, also known as Glutamic-Oxaloacetic Transaminase
ALK	Anaplastic Lymphoma Kinase
AUC	Area Under the Concentration-Time Curve
BUN	Blood Urea Nitrogen
BICR	Blinded Independent Central Review
CDE	Center for Drug Evaluation
Cr	Serum Creatinine
CI	Confidence Interval
CL	Chloride
C_{max}	Maximum Concentration
Cr	Creatinine
CR	Complete Response
CTCAE	Common Terminology Criteria for Adverse Events
DBIL	Direct Bilirubin
DoR	Duration of Response
DCR	Disease Control Rate
EAS	Efficacy Analysis Set
EC	Ethics Committee
ECG	Electrocardiogram
eCRF	Electronic Case Report Form
EDC	Electronic Data Capture
EGFR	Epidermal Growth Factor Receptor
ECOG	Eastern Cooperative Oncology Group
EORTC	European Organisation for Research and Treatment of Cancer
FAS	Full Analysis Set
GCP	Good Clinical Practice
HB	Haemoglobin
HDL	High-Density Lipoprotein Cholesterol
HbA1c	Glycosylated haemoglobin
ICF	Informed Consent Form
INR	International Normalized Ratio
IARC	International Agency for Research on Cancer

IMP	Investigational Medicinal Product
IDMC	Independent Data Monitoring Committee
K	Potassium
LDL	Low-Density Lipoprotein Cholesterol
LY	Lymphocytes
LVEF	Left Ventricular Ejection Fraction
MedDRA	Medical Dictionary for Regulatory Activities
Na	Sodium
NSCLC	Non-Small Cell Lung Cancer
NEUT	Neutrophils
NOAEL	No Observed Adverse Reaction Dose
NMPA	National Medical Products Administration
ORR	Objective Response Rate
OS	Overall Survival
OSR	Overall Survival Rate
PK	Pharmacokinetics
PPS	Per-Protocol Set
PFS	Progression-Free Survival
PT	Prothrombin Time
PR	Partial Response
PLT	Platelet count
PRO	Patient-Reported Outcome
RBC	Red blood cell count
RECIST	Response Evaluation Criteria in Solid Tumors
RANO-BM	Response Assessment in Neuro-Oncology Brain Metastases Criteria
QTcF	QT Interval Corrected for Heart Rate Using Fridericia's Formula
QoL	Quality of Life Score
SAE	Serious Adverse Event
SAP	Statistical Analysis Plan
SOP	Standard Operating Procedures
SS	Safety Analysis Set
$t_{1/2}$	Half-Life
TC	Total Cholesterol
TBIL	Total Bilirubin
TP	Total Protein
TG	Triglycerides
TKI	Tyrosine Kinase Inhibitors
TTD	Time to Deterioration
ULN	Upper Limit of Normal

UA	Uric Acid
UREA	Urea
WBC	White blood cell count
WHO	World Health Organization

1. Introduction

1.1 Investigational Medicinal Product

1.1.1 Nomenclature

XZP-3621 Tablets (active ingredient: XZP-3621) is an ALK tyrosine kinase inhibitor developed by Shandong Xuanzhu Pharma Co., Ltd., and its proposed indication is ALK+ advanced non-small cell lung cancer.

Generic Name (Chinese): XZP-3621

Generic Name (English): /

Molecular Formula: $C_{27}H_{32}ClN_5O_4S$

Molecular Weight: 558.09

1.1.2 Physicochemical Properties

This product is an off-white to yellow powder, with no or almost no hygroscopicity; it is freely soluble in N,N-dimethylformamide, soluble in dimethyl sulfoxide, slightly soluble in anhydrous ethanol, and insoluble in aqueous solution and 0.1 mol/L sodium hydroxide solution, with a melting point of 197.5~200.0°C.

1.1.3 Mechanism of Action

XZP-3621 is an ALK tyrosine kinase inhibitor that inhibits autophosphorylation of ALK protein and downstream target protein STAT3 phosphorylation by acting on the ATP binding site of the ALK kinase region, thereby inhibiting the proliferation, growth and survival of tumor cells, and achieving the purpose of inhibiting tumors.

1.2 Study Background

Lung cancer is the most common cancer worldwide. Lung cancer is divided into small cell lung cancer and non-small cell lung cancer (NSCLC) from the perspective of pathological tissue morphology. NSCLC is the most common fatal neoplasm malignant worldwide, and patients are often in the advanced stage of the disease when diagnosed.

Basic research into the etiology of lung cancer has found that abnormal gene regulation and gene polymorphisms play an important role in the pathogenesis of lung cancer. With the development of gene analyses and molecular diagnostic technologies, breakthroughs have been made in the diagnosis and treatment of lung cancer based on genetic characteristics. An increasing number of lung cancer driver genes have been discovered by scientists and used for new drug development, especially in lung adenocarcinoma, where approximately 60% of driver

genes have been identified. Compared to chemotherapy, targeted therapy can significantly improve response rates, prolong progression-free survival and overall survival, thus becoming an important treatment method for NSCLC [1]. The American Society of Clinical Oncology (ASCO) Annual Meeting in 2013 released the mutation frequencies of 11 driver genes in NSCLC. The mutation rate of functional driver genes in lung adenocarcinoma is approximately 60%, among which KRAS (kirsten rat sarcoma viral oncogene), EGFR, and EML4-ALK (Echinoderm microtubule-associated protein-like4-anaplastic lymphoma kinase) fusion are the most common driver genes, accounting for approximately 35%-40% [2][3].

The positive rate of ALK gene rearrangement (i.e., EML4-ALK fusion gene) in lung adenocarcinoma in the Chinese population is 5.1%. Studies based on the Chinese population have found that the incidence of ALK gene rearrangement is as high as 18.5% in young patients younger than 51 years old; another study found that the incidence of ALK gene rearrangement is nearly 20% in young patients younger than 40 years old [2][5].

Crizotinib Capsules are the first approved drug for the treatment of anaplastic lymphoma kinase (ALK)+ NSCLC. Although most patients with ALK+ NSCLC benefit from Crizotinib treatment, some do not benefit due to intrinsic drug resistance, while others who initially benefit will develop acquired resistance. It has been reported that in ALK+ NSCLC cases with Crizotinib resistance, the resistance rate due to mutations in the ALK kinase domain is 35-40% [6]. ALK+ NSCLC patients usually develop drug resistance within 1-2 years of receiving Crizotinib treatment, and due to its low drug concentration through the blood-brain barrier, central nervous system disease progression often occurs. Therefore, solving the problem of secondary drug resistance mutation of targeted drugs is a research hotspot for the development of new generation of targeted drugs [7].

This investigational medicinal product, XZP-3621 Tablets, is a Class 1 innovative drug. Preclinical studies have shown that XZP-3621 Tablets has excellent *in vitro* inhibitory activity and *in vivo* pharmacodynamic activity against multiple drug resistance mutation sites generated after the use of Crizotinib Capsules, an ALK inhibitor. Preliminary results of Phase 1 clinical studies have shown that XZP-3621 Tablets is effective against ALK+ NSCLC patients. Therefore, a Phase 3, multi-center, randomized, open-label clinical study with an expanded sample size is planned to compare the efficacy and safety of XZP-3621 Tablets versus Crizotinib Capsules in patients with ALK+ advanced NSCLC.

1.3 Preclinical Studies

1.3.1 Preclinical Pharmacodynamic Studies

XZP-3621 exhibits good inhibitory activity against ALK fusion mutations, with an *in vitro* enzymatic inhibition activity IC_{50} value of 0.21 nM. The inhibitory activity against cell lines

with different ALK fusions is superior to the marketed drugs Crizotinib and Ceritinib. The IC_{50} value in the NCI-H3122 cell line is 3 nM, which is superior to Crizotinib at 334 nM and Ceritinib at 40 nM. *In vivo* pharmacodynamic studies have shown that XZP-3621 exhibited significant anti-tumor activity in the H2228 (EML4-ALK) human lung cancer xenograft model, showing a clear dose-response relationship. With increasing doses, it produced moderate to strong anti-tumor effects, and the medium and high dose groups had a significant inhibitory effect on tumor growth. In the H2228-luc lung cancer intracranial brain metastasis model, XZP-3621 exhibited significant anti-tumor activity during the monitoring of tumor bioluminescence signals. The optical signal intensity at the tumor site was comparable to that of the control drug Ceritinib. Meanwhile, it significantly prolonged the survival time of animals, with a median survival time 6 days longer than that of the solvent control group and the Crizotinib group, and comparable to the Ceritinib group. Safety pharmacology studies showed no effects on the respiratory, central nervous, and cardiovascular systems.

1.3.2 Preclinical Pharmacokinetic Studies

XZP-3621 had good pharmacokinetic parameters, with rapid absorption, long half-life, and no significant impact of gender differences on pharmacokinetic parameters. Within a certain dose range, exposure was linear with the dose, and there was no significant accumulation of exposure after multiple doses. XZP-3621 was widely distributed in tissues (mainly in the duodenum, gastric wall, kidney, spleen, and other organs) and could penetrate the blood-brain barrier to enter brain tissue. Following oral gavage administration of XZP-3621 to SD rats, it was mainly excreted via feces, with a recovery rate of 96.9% of the administered dose. In male and female BDC rats, the recovery rate in bile after a single oral gavage administration was 8.58% of the administered dose. In rat plasma, bile, urine, and feces samples, a total of 4 metabolites were identified in addition to the parent drug, which were M1 (S-desisopropyl metabolite), M2 (S-desisopropyl and oxidation metabolite), M4a (oxidation and sulfate conjugate), and M4 (oxidation metabolite). M4 was the major metabolite in rat plasma, urine, bile, and feces.

XZP-3621 exhibited low permeability in Caco-2 cells; it was highly likely to be a substrate of P-gp, and might be a substrate of BCRP; it had good stability in human liver microsomes, and the structure of metabolites was relatively simple; the plasma protein binding rate was high (≥95.0%); it had no significant induction effect on human hepatocyte CYP1A2, CYP2B6, and CYP3A4; it had almost no inhibitory effect on CYP2C19, CYP2D6, and CYP1A2, and had a moderate inhibitory effect on CYP3A4, CYP2C9, CYP2B6, and CYP2C8. The clinical significance of this inhibitory effect needs to be considered in combination with clinical plasma concentrations.

1.3.3 Preclinical Toxicology Studies

The genetic toxicity evaluation tests (Ames, micronucleus, and chromosomal aberration) of XZP-3621 were all negative.

Repeated four-week gavage administration of the test article XZP-3621 to SD rats at doses of 40, 80, 150/120 mg/kg/day resulted in a No Observed Adverse Effect Level (NOAEL) of 80 mg/kg/day. In the ≤80 mg/kg/day dose group, hypophagia, weight loss, and changes in hematological parameters, as well as histopathological changes related to the test article in the bone marrow (sternum), spleen, liver, and adrenal glands at 80 mg/kg, were all considered non-adverse reactions due to the magnitude and extent of the changes. The 150/120 mg/kg/day dose resulted in animal death, accompanied by adverse clinical reactions, weight loss, hypophagia, changes in hematology (RET, LY, NE, MONO, FIB) and blood chemistry (ALT, AST, ALP, GGT, UREA, GLU) parameters. Organs with test article-related histopathological changes included: bone marrow (sternum), spleen, thymus, submandibular and mesenteric lymph nodes, liver, pituitary gland, adrenal gland, pancreas, submandibular gland, and prostate. Only moderate to severe cell depletion in the bone marrow (sternum), mild hepatocellular necrosis in the liver, and moderate to severe lymphocyte depletion and increased lymphocyte lysis in lymphoid tissues were considered adverse reactions. At the end of the recovery period, there were no clinical signs, weight loss, decreased food consumption, hematological, hemocoagulation, and serum chemistry parameters, or pathological changes as described above. At a dose of 80 mg/kg on Day 28, the exposure ratio of the main metabolite XZP-5089 to the parent drug XZP-3621 was 29.3% in male rats and 23.4% in female rats.

Repeated four-week gavage administration of XZP-3621 to cynomolgus monkeys at doses of 6, 12, and 20 mg/kg/day resulted in reversible abnormal faeces (soft or watery stools) and vomiting only at 12 mg/kg, so the NOAEL was 12 mg/kg/day. At a dose of 20 mg/kg/day, 2 animal deaths occurred, accompanied by clinical adverse reactions (symptoms of decreased activity, cold body, and tremor), weight loss, and body temperature decreased. Clinical pathological changes included increased NEU percentage, decreased LYM percentage, increased ALT, AST, GLU, UREA, CRE and CK levels. Histopathological changes related to the test article included the thymus and spleen, but both clinical pathological changes and histopathological changes were non-adverse reactions. All test article-related changes were recovered after a 28-day recovery period.

Repeated oral gavage administration of XZP-3621 to SD rats for thirteen weeks at doses of 40, 60, and 80 mg/kg resulted in a highest non-serious toxic dose (HNSTD) of 80 mg/kg/day. No moribund or death animals were observed in this study. No test article-related changes in serum biochemistry, urine, and ophthalmological examination were observed. Clinical observations: Drooling was observed in all groups during the administration process, and dose-related

abdominal distension occurred from days 36-65, which was not observed during the recovery period. Hematology: At the end of the administration period, compared with the control group, LY% decreased in the three dose groups, and NE and NE%, MO and MO%, and FIB increased in the three dose groups; at the end of the recovery period, RDW, RET and RET% increased in male animals in the three dose groups, and RET and RET% increased in female animals ≥60 mg/kg. Organ weight: At the end of the administration period, the liver-to-body weight ratio increased and pituitary weight decreased in animals in the three dose groups, and thymus weight decreased in female animals in the three dose groups; during the recovery period, only the pituitary weight of females did not fully recover. Gross observation: At the end of the administration period, small spleens were observed in ≥60 mg/kg females and/or males, and small thymus was observed in 80 mg/kg females, which was not observed during the recovery period. The above changes in clinical observations, hematology, organ weight, and gross observation were considered to be non-adverse reaction related to the test article because the magnitude of the changes was small or within the historical range. Histopathology: At the end of the administration period, histopathological changes were observed in the pituitary gland, duodenum, liver, bone marrow, thymus, and mesenteric lymph nodes. Because the degree of change was mild or adaptive, and could be recovered during the recovery period, the above changes were considered to be non-harmful effects related to the test article. At the end of the administration period, compared with the control group, the weight gain of animals in the three dose groups was slow and dose-dependent. The weight of 80 mg/kg male and female animals was 19.59% and 33.80% lower than that of the control group, respectively. At the same time, marasmus was found in individual animals in the 80 mg/kg group (recoverable during the administration process); the food intake of the three dose groups was lower than that of the control group, and decreased in a dose-dependent manner. At the end of the recovery period, the weight and food intake showed a recovery trend, but were still lower than those of the control group. The body weight of the animals in the administration group was significantly lower than that of the control group in the same period, and did not fully recover at the end of the recovery period. Therefore, the above changes were considered to be harmful changes related to the test article. In summary, SD rats were orally administered the test article XZP-3621 at doses of 0, 40, 60, and 80 mg/kg/day, once a day, for 13 weeks, and no animal death was observed. Test article-related non-adverse reaction changes were observed in clinical symptoms, decreased food intake, hematology, blood coagulation, and pathological changes. Test article-related adverse reaction were observed in slow weight gain in all dose groups, which did not fully recover at the end of the recovery period. No drug-related harmful effects were found after a 28-day recovery period. Under the conditions of this experiment, the HNSTD (highest non-serious toxic dose) was 80 mg/kg/day. At this dose level, on Day 91, the systemic exposure of XZP-3621 (C_{max} and AUC_{0-24h}) in males was 445 ng/mL and 4120 h*ng/mL, and

the systemic exposure of XZP-3621 (C_{max} and AUC_{0-24h}) in females was 748 ng/mL and 6330 h*ng/mL.

Repeated oral gavage administration of XZP-3621 to cynomolgus monkeys for thirteen weeks at doses of 4, 8, and 12 mg/kg resulted in a No Observed Adverse Effect Level (NOAEL) of 12 mg/kg/day. No test article-related changes in mortality, weight, food consumption, body temperature, ophthalmological examination, electrocardiogram, hematology, coagulation parameters, urine analysis, macroscopic lesions, organ weight, and histopathology were observed at all dose levels in this study. Test article-related clinical signs during the dosing period were limited to abnormal faeces (soft/watery stools) and moderate/small amounts of vomiting observed in animals in all dose groups, including the vehicle control group or also seen prior to the study. Since the frequency of abnormal faeces or vomiting was higher in the dosing groups after dosing than in the control group, it was considered to be a test article-related change. All of these test article-related signs were reversible during the recovery period and were non-harmful changes. Compared with the control group, the only test article-related serum biochemical change was a significant increase in alanine aminotransferase (ALT) in male animals at the 8 and 12 mg/kg/day doses at mid-dosing, reaching 105% and 116%, respectively. This change was completely reversed during the recovery period. It was considered a reversible, non-harmful change. In summary, cynomolgus monkeys tolerated XZP-3621 well when administered once daily by oral gavage or nasogastric administration at doses of 4, 8, 12 mg/kg/day for 13 consecutive weeks. No test article administration-related harmful reactions in animal death, clinical signs, weight, food consumption, body temperature, ophthalmological examination, electrocardiogram, hematology, coagulation parameters, serum biochemistry and urine analysis, macroscopic lesions, organ weight, and histopathology were produced in any dose group. Therefore, the NOAEL under the conditions of this study was considered to be 12 mg/kg/day. At this dose level, the exposure ($AUC_{0-24\,h}$) in male and female animals after the last dose was 1070 ± 274 h*ng/mL and 1170 ± 619 h*ng/mL, respectively, and the maximum concentration (C_{max}) was 109 ± 19.8 ng/mL and 115 ± 57.3 ng/mL, respectively.

1.4 Clinical Studies

The ongoing clinical study of XZP-3621 Tablets is "A Phase 1, Multicenter, Open-Label, Dose-Escalation and Dose-Expansion Clinical Study of XZP-3621 in Chinese Subjects with Advanced Non-Small Cell Lung Cancer Harbouring ALK or ROS1 Rearrangement".

1.4.1 Preliminary Analysis of Clinical Pharmacokinetic Data

- **Pharmacokinetic Studies After Single and Multiple Doses**

As of August 11, 2021, pharmacokinetic (PK) characteristics were evaluated in 53 NSCLC subjects with ALK rearrangement or ROS1 rearrangement.

Following a single dose of XZP-3621 at 50-600 mg, absorption was rapid, with t_{max} at 3-4 h; within this dose range, exposure increased with increasing dose. After absorption, XZP-3621 was moderately cleared from the body, with $t_{1/2}$ at 20.5-25.9 h. XZP-3621 showed high inter-individual variability in exposure at each dose examined. Simultaneously, the metabolite XZP-5089 was measured, and the results showed that the exposure of this metabolite in plasma was low, accounting for less than 5% of the AUC of the parent drug.

Following continuous administration of XZP-3621 at 100-600 mg, t_{max} was 2-5 h; within this dose range, XZP-3621 exposure did not increase with increasing dose, and C_{max} and AUC showed high inter-individual variability, belonging to high variability. Similar to single-dose administration, the metabolite XZP-5089 had low exposure in plasma, accounting for less than 5% of the AUC of the parent drug. XZP-3621 showed no significant accumulation in the body within the examined dose range.

1.4.2 Preliminary Safety Analysis

As of August 30, 2021, a total of 76 subjects received the investigational medicinal product therapy, including 2 subjects at 50 mg, 4 subjects at 100 mg, 3 subjects at 200 mg, 8 subjects at 300 mg, 14 subjects at 400 mg, 37 subjects at 500 mg, and 8 subjects at 600 mg.

Preliminary safety analysis showed that the main adverse reactions of XZP-3621 included diarrhoea, vomiting, nausea, blood glucose increased, weight decreased, decreased appetite, aspartate aminotransferase increased, blood triglycerides increased, hypertriglyceridaemia, blood uric acid increased, alanine aminotransferase increased, fatigue, abdominal pain, and anaemia.

72 (94.7%) patients experienced drug-related AEs, with only 1 drug-related serious adverse event (SAE). This patient was a 60-year-old female who developed acute gastritis after medication, mainly manifested as stomach pain and nausea, CTCAE Grade 2, and the severity criteria was leading to hospitalization. The action taken for the investigational product was to continue the medication, and the outcome was recovered. Both the investigator and the sponsor assessed it as possibly related to the drug.

Among drug-related AEs, AEs with an incidence ≥10% were: diarrhoea in 62 subjects (81.6%), vomiting in 48 subjects (63.2%), nausea in 36 subjects (47.4%), weight decreased in 21 subjects (27.6%), decreased appetite in 18 subjects (23.7%), anaemia in 16 subjects (21.1%), blood glucose increased in 16 subjects (21.1%), aspartate aminotransferase increased in 15 subjects (19.7%), abdominal pain in 14 subjects (18.4%), blood uric acid increased in 14 subjects (18.4%), alanine aminotransferase increased in 13 subjects (17.1%), blood triglycerides increased in 13 subjects (17.1%), fatigue in 12 subjects (15.8%), white blood cell count decreased in 10 subjects (13.2%), blood creatinine increased in 10 subjects (13.2%), abdominal

discomfort in 9 subjects (11.8%), hypertriglyceridaemia in 9 subjects (11.8%), neutrophil count decreased in 9 subjects (11.8%), hyperuricemia in 8 subjects (10.5%), blood cholesterol increased in 8 subjects (10.5%), and bilirubin conjugated increased in 8 subjects (10.5%).

1.4.3 Preliminary Efficacy Analysis

In subjects with at least one tumor assessment after XZP-3621 treatment, among 24 treatment-naïve ALK+ NSCLC patients, 21 subjects (87.5%) were assessed as PR, and 2 subjects (8.3%) were assessed as SD. The ORR was 87.5%, the DCR was 95.83%, the median PFS was not yet reached, and the 12-month PFS rate was 83%. Among 4 patients with crizotinib treatment failure, 3 patients were PR and 1 case was PD. Among 13 second-generation ALK inhibitor-treated patients (some patients had undergone first-generation ALK inhibitor, multiple second-generation ALK inhibitor treatments, and multi-line chemotherapy), 2 patients were PR, 3 patients were SD, 4 patients were unevaluable (currently assessed as SD), and 4 patients were PD.

Based on the above safety, efficacy, and pharmacokinetic data, after preliminary ER analysis, communication and confirmation between the sponsor and the principal investigator, the RP2D was determined to be 500 mg, and a Phase 3 multicenter, randomized, open-label clinical study is planned to be conducted at this dose to compare the efficacy and safety of XZP-3621 Tablets versus Crizotinib Capsules in patients with ALK+ advanced NSCLC.

1.5 Study Rationale

Crizotinib Capsules are the first approved drug for the treatment of anaplastic lymphoma kinase (ALK)+ NSCLC and remain the first-line standard of care for patients with ALK+ NSCLC. Although most patients with ALK+ NSCLC benefit from crizotinib treatment, some do not benefit due to intrinsic drug resistance, while others who initially benefit will develop acquired resistance. It has been reported that in ALK+ NSCLC cases with crizotinib resistance, the resistance rate due to mutations in the ALK kinase domain is 35-40% [6]. Next-generation ALK/ROS1 TKIs are being developed to address the reasons for crizotinib treatment failure.

The investigational medicinal product, XZP-3621 Tablets, is a Class 1 innovative drug. Preclinical studies have shown that XZP-3621 Tablets has excellent *in vitro* inhibitory activity and *in vivo* pharmacodynamic activity against multiple drug resistance mutation sites generated after the use of other ALK inhibitors. Combined with the preliminary results of the Phase 1 clinical study, XZP-3621 Tablets is effective for treatment-naïve advanced ALK+ NSCLC patients. Therefore, a Phase 3, multicenter, open-label, randomized, parallel-controlled clinical study is planned to evaluate the efficacy and safety of XZP-3621 Tablet treatment for this population.

1.6 Study Risks/Benefits

1.6.1 Risk Analysis

- **Risk and Management of Adverse Reactions:**
  - ✧ XZP-3621 Tablets: Preliminary safety analysis of the Phase 1 clinical study showed that no dose-limiting toxicities (DLT) occurred during the dose escalation part, no maximum tolerated dose (MTD) was observed, and the maximum administered dose was 600 mg/day. As of August 30, 2021, among the 76 patients in the Phase 1 clinical study, the most common drug-related AEs (incidence ≥20%) observed were: diarrhoea, vomiting, nausea, weight decreased, decreased appetite, anaemia, and blood glucose increased. There are no confirmed expected serious adverse reactions. The vast majority of the above adverse reactions are common to drugs of the same class. The study protocol also specifies detailed dose adjustment measures to ensure the safety of subjects.
  - ✧ Crizotinib Capsules: The post-marketing package insert shows that the most common adverse reactions (≥25%) of Crizotinib Capsules are visual impairment, nausea, diarrhoea, vomiting, oedema, constipation, transaminases increased, fatigue, decreased appetite, upper respiratory tract infection, dizziness and neuropathy; very common (≥10%) adverse reactions are anaemia (15%); common (≥1% to <10%) adverse reactions are blood creatinine increased (8%), blood alkaline phosphatase

increased (7%), oesophagitis (2%), decreased blood testosterone (2%) and cardiac failure (1%); uncommon (≥0.1% to <1%) but potentially serious adverse reactions are gastrointestinal perforation (<1%), acute renal failure (<1%) and renal failure (<1%). Refer to the approved package insert of Crizotinib Capsules (Xalkori) for management strategies.

1.6.2 Benefit Analysis

- **Providing potentially effective therapeutic drugs for subjects with ALK+ advanced NSCLC.** According to the preliminary efficacy analysis of the Phase 1 clinical study, XZP-3621 Tablets showed good efficacy in patients who were treatment-naive to ALK inhibitors within the 200-500 mg dose range, and a certain proportion of objective response rate provided a basis for subsequent Phase 3 clinical studies.
- **Subjects can receive comprehensive treatment and observation.** After patients participate in this study, they will undergo regular and more detailed examinations and observations than the general clinical diagnosis and treatment process, according to the study procedure. Patients' conditions can be more closely observed and treated by the study physicians, which is more conducive to controlling adverse reactions of anti-tumor treatment, adjusting the dose of the investigational medicinal product in a timely manner according to adverse reactions, detecting disease progression early and taking corresponding measures, and is more conducive to controlling patients' diseases.

In this study, the risk to subjects will be minimized through strict adherence to inclusion/exclusion criteria and close clinical monitoring. Throughout the study, investigators will closely monitor clinically and laboratory-detected adverse events as specified in the protocol to ensure the safety of subjects. If a subject develops any condition/disease or adverse event, the investigator should immediately determine whether the subject should continue to participate in the study or withdraw from the study early, and take appropriate treatment measures for the subject as needed.

Based on a comprehensive analysis of the aforementioned risks and benefits, and with the implementation of corresponding prevention and control measures, the risks can be effectively controlled. Furthermore, subjects may receive potentially effective treatments and comprehensive observation and treatment. Therefore, the benefits to subjects participating in this study outweigh the risks.

2. Study Objectives

2.1 Primary Objective

- To compare the efficacy of XZP-3621 Tablets versus Crizotinib Capsules in patients with ALK+ advanced non-small cell lung cancer (NSCLC) (investigator-assessed PFS).

2.2 Secondary Objectives

- To compare the efficacy of XZP-3621 Tablets versus Crizotinib Capsules in patients with ALK+ advanced NSCLC (BICR-assessed PFS; ORR, DoR, DCR, OS, OSR, IC-ORR, and IC-DoR assessed by BICR and investigators, respectively);
- To compare the safety and tolerability of XZP-3621 Tablets versus Crizotinib Capsules in patients with ALK+ advanced NSCLC;
- To evaluate the population pharmacokinetic characteristics of XZP-3621 in Chinese subjects with ALK+ advanced NSCLC.

2.3 Exploratory Objectives

- To compare disease/symptom-related patient-reported outcomes (PRO) and health-related quality of life (HRQOL) scores after treatment with XZP-3621 Tablets versus Crizotinib Capsules in patients with ALK+ advanced NSCLC;
- To compare the health status of subjects with ALK+ advanced NSCLC treated with XZP-3621 Tablets versus Crizotinib Capsules.

3. Study Endpoints

3.1 Primary Endpoint

- PFS assessed by investigator (RECIST v1.1 criteria).

3.2 Secondary Endpoints

- PFS assessed by Blinded Independent Central Review (BICR) (RECIST v1.1 criteria);
- ORR, DoR, DCR, OS, and OSR assessed by BICR and investigator, respectively;
- Objective response rate (IC-ORR) and duration of response (IC-DOR) in brain metastasis patients with measurable lesions assessed by BICR and investigators (RECIST v1.1 criteria and RANO-BM criteria);
- Safety**:** AE/evaluation (assessed per CTCAE v5.0 criteria); physical examination and vital signs; laboratory tests (hematology, blood chemistry, coagulation function, and urinalysis); 12-lead ECG and echocardiography;
- Pharmacokinetic parameters: population pharmacokinetic characteristics of subjects.

3.3 Exploratory Endpoints

- Time to Deterioration (TTD), defined as the time from randomization to first deterioration in EORTC QLQ-C30 scale score.
- Mean scores and mean changes from baseline in the European Organisation for Research and Treatment of Cancer Quality of Life Core Questionnaire (EORTC QLQ-C30).
- Mean scores and mean changes from baseline in the European Organisation for Research and Treatment of Cancer Lung Cancer: a modular supplement to the EORTC Core Quality of Life Questionnaire (EORTC QLQ-LC13).
- Health Utility Index of the European Quality of Life 5-Dimension 5-Level Scale Questionnaire (EQ-5D-5L).

4. Study Design

4.1 Study Design Overview

The overall design of this study follows the current Regulations for the Implementation of the Drug Administration Law of the People's Republic of China" [8], "Good Clinical Practice" [9], CFDA "Technical Guidelines for Clinical Studies of Anti-tumor Drugs" [10], and ICH Guideline "Nonclinical Evaluation for Anticancer Pharmaceuticals" [11].

This study is a Phase 3, multicenter, randomized, open-label, parallel-controlled clinical study. The study plans to enroll 268 patients with ALK+ advanced NSCLC who had received only one chemotherapy regimen or no prior systemic antitumor therapy. Patients will be randomized 1:1 to either the test group (134 patients) or control group (134 patients) to compare the efficacy and safety of XZP-3621 Tablets versus Crizotinib Capsules in treating patients with ALK+ advanced NSCLC.

The entire study consists of a screening period, a treatment period, a safety follow-up period, and a survival follow-up period (see Figure 1).

Test group dosing method: XZP-3621 Tablets, 500 mg once daily, administered with meals or within 30 minutes after meals, continuously administered with 4-week treatment cycles.

Control group dosing method: Crizotinib Capsules, 250 mg twice daily, administered with or without food, continuously administered with 4-week treatment cycles.

Stratification factors: 1. Brain metastasis (Yes, No); 2. Chemotherapy (Yes, No).

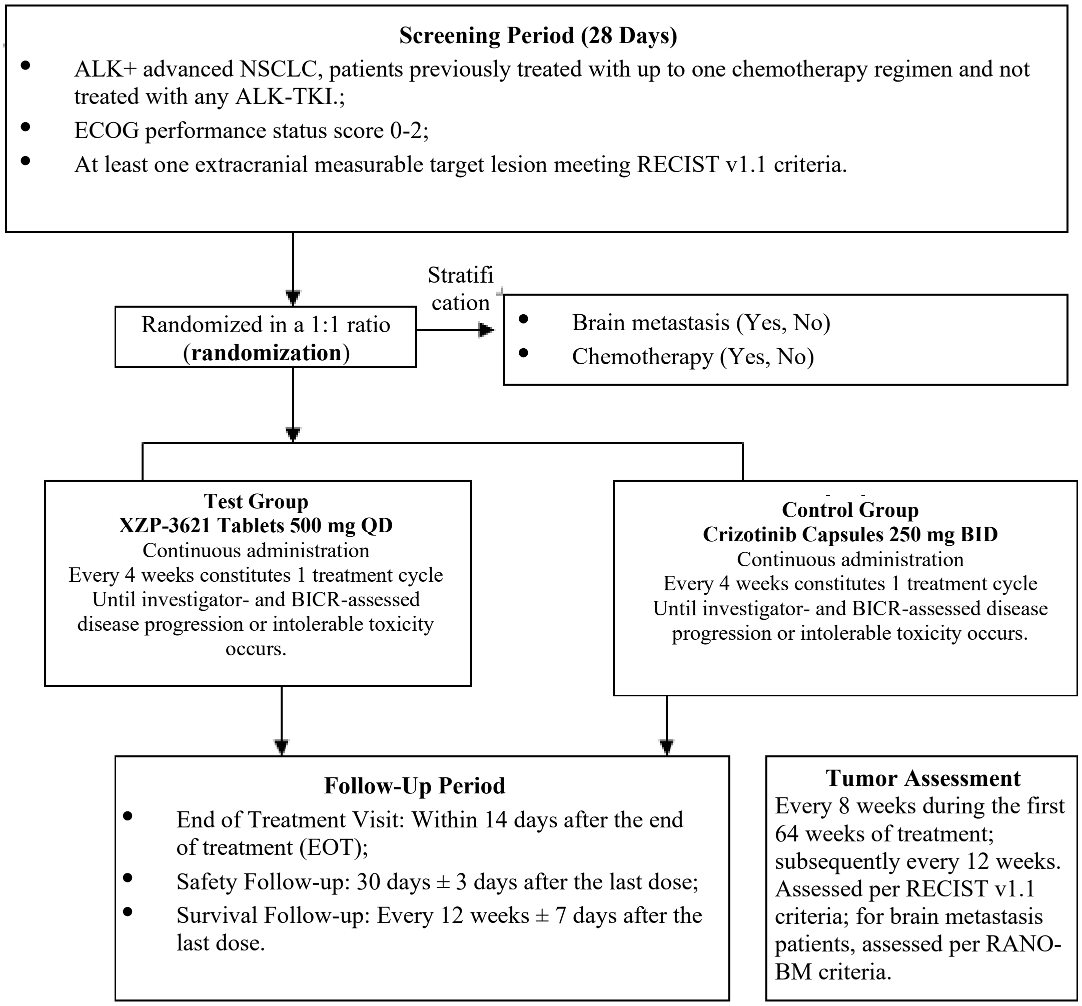

Figure 1 Study Design Diagram

Treatment continues until subjects experience investigator- and BICR-assessed disease progression, intolerable toxicity, withdrawal of consent, death, investigator determination of unsuitability for continued treatment, or subject decision to discontinue study treatment, whichever occurs first.

During the study, if isolated asymptomatic central nervous system progression (such as new central nervous system oligometastases) occurs, local treatment (such as stereotactic radiotherapy or surgery) should be administered, followed by continued XZP-3621 Tablets (test group) or Crizotinib Capsules (control group) treatment until systemic disease progression and/or symptomatic central nervous system progression. For non-isolated symptomatic central nervous system progression, post-progression continuation of study treatment is determined by the investigator based on whether patients may continue benefiting from respective therapies. If subjects discontinue study treatment after investigator- and BICR-assessed disease

progression, subsequent treatment will be at the discretion of the investigator according to local practice. Information on the nature and duration of subsequent treatment will be collected.

Patients who discontinue treatment before disease progression (e.g., due to intolerable toxicity) will continue tumor assessment follow-up as scheduled until disease progression or initiation of new antitumor therapy.

4.2 Independent Data Monitoring Committee (IDMC)

An IDMC will be established for this study to provide oversight of study safety and conduct to protect the ethical and safety rights of subjects and the scientific integrity of the study. The IDMC's meeting arrangements, composition, responsibilities, operation, and management will be described in a separate IDMC charter.

4.3 Interim Analysis

This study is designed to include one planned interim analysis. The primary endpoint is investigator-assessed PFS. The interim analysis will be conducted when approximately 70% (about 103) of projected PFS events are collected. Interim analysis thresholds and efficacy boundaries for each stage are as follows:

Analysis Phase	Event Count	Z Boundary Value	One-Sided Nominal Significance Level
First Analysis	103	2.44	0.0074
Final Analysis	147	2.00	0.0228

Note:

1. The alpha-spending function will use Lan-DeMets (O’Brien-Fleming) method to control the overall type I error at one-sided 0.025;
2. The actual nominal significance level and boundaries for interim analysis will be determined based on the proportion of events observed at that time. The nominal significance level and boundaries for final analysis will be adjusted accordingly.

4.4 End of Study

This study is considered complete overall when the required number of 147 PFS events for the primary analysis is reached, or when the sponsor confirms that sufficient OS events have been collected, or when the sponsor terminates this study (whichever occurs first).

4.5 Treatment after End of Study

For subjects who are still receiving study treatment at the end of the study, if they achieve stable disease or response in the efficacy evaluation, and the adverse reaction is tolerable, they may continue to receive treatment through another extension study or other forms agreed upon by the sponsor after obtaining approval from health regulatory agencies and the ethics committee.

The sponsor reserves the right to stop providing the investigational medicinal product in any of the following circumstances:

- Marketing application is rejected by health regulatory authorities;
- The study is terminated due to safety concerns.
- The subject is able to obtain the drug from a government or privately initiated medical program;
- An alternative treatment drug is available in the local market.

5. Study Population

Subjects who meet all inclusion criteria and do not meet any exclusion criteria may be enrolled in this study.

5.1 Inclusion Criteria

Subjects must meet all of the following inclusion criteria to be eligible for this study:

1. Male or female subjects aged 18-75 years (inclusive);
2. Histologically or cytologically confirmed stage IV NSCLC or stage IIIB/IIIC NSCLC that is not amenable to radical radiotherapy (according to the 8th edition of the International Association for the Study of Lung Cancer staging) with ALK+ status confirmed by nationally approved detection methods (Ventana D5F3 IHC, FISH, PCR, or NGS). Subjects without prior detection reports must provide tissue samples for confirmation of the ALK gene;
3. Subjects who have not previously received systemic treatment for NSCLC, including ALK-TKIs, immunotherapy, or biological therapies (cancer vaccines, cytokines, or growth factors controlling cancer), etc. (Patients who have progressed or developed intolerable toxicity after one prior chemotherapy regimen are eligible);

   Note: A prior chemotherapy regimen refers to systemic chemotherapy administered after diagnosis of stage IV NSCLC or unresectable stage IIIB/IIIC NSCLC. Progression within 6 months of neoadjuvant/adjuvant chemotherapy is considered as one prior chemotherapy regimen.
4. ECOG performance status score 0-2 (Appendix 2);
5. Subjects must have at least one extracranial measurable target lesion meeting RECIST v1.1 criteria that has not been previously irradiated. Previously irradiated lesions may only be considered target lesions if definitive progression post-radiation is documented;
6. Subjects without brain metastases or with asymptomatic brain metastases are eligible, provided:
   a) Untreated and not requiring corticosteroids currently, or
   b) Prior to randomization, local treatment has been completed, and patients have fully recovered from the acute effects of radiotherapy or surgery, corticosteroid hormone therapy has been discontinued for at least 4 weeks in these patients with brain metastases and neurological symptoms are stable;
   c) Subjects with leptomeningeal disorder (LMD) or carcinomatous meningitis (CM) visible on MRI, or positive CSF cytology at baseline must be excluded.

7. Investigator-assessed expected survival ≥12 weeks;
8. Adequate organ function at baseline evaluation with laboratory tests meeting:
   a) **Hematology**: Absolute neutrophil count $\geq 1.5\times10^9$/L, platelet count $\geq 90\times10^9$/L, haemoglobin ≥90 g/L;
   b) **Liver Function**: Serum total bilirubin ≤1.5× the upper limit of normal (ULN); ALT and AST ≤2.5× ULN (≤ 5× ULN in subjects with metastases to liver);
   c) **Renal Function**: Blood creatinine ≤1.5× ULN or Ccr ≥50 mL/min (≥0.835 mL/s by Cockcroft-Gault formula, Appendix 6);
   d) Serum amylase (AMS) ≤1.0×ULN (subjects with amylase increased 1-2×ULN are eligible if no other signs or clinical evidence of pancreatic disorder are present);
   e) Glycosylated haemoglobin ≤7.0%;
9. All surgeries and prior radiotherapy (except palliative)/operations must be completed ≥4 weeks before randomization; Palliative radiation must be completed ≥1 week before randomization;
10. All non-hematologic toxicities related to other anti-tumor therapies must have recovered to ≤ Grade 2 (except alopecia);
11. All women of childbearing potential must have negative pregnancy test within 3 days before treatment. Male or female subjects of childbearing potential must agree to use effective contraception methods during the study and for 90 days after last dose, such as double-barrier contraception, condoms, oral or injection contraceptive drugs, intrauterine devices, etc.;
12. Capable and willing to provide written informed consent before undergoing any study-related procedures and to comply with the treatment plan.

5.2 Exclusion Criteria

Subjects who meet any of the following exclusion criteria will not be allowed to participate in this study:

1. Subjects with concurrent small cell lung cancer;
2. Uncontrolled malignant pleural/peritoneal effusion and/or pericardial effusion (uncontrolled refers to significant reaccumulation within one week after drainage);
3. Diagnosis of any other neoplasm malignant within 3 years before enrollment (except adequately treated and stable basal cell carcinoma or early gastrointestinal (GI) cancer removed endoscopically or carcinoma in situ of cervix);

4. History of organ transplant, hematopoietic stem cell or bone marrow transplant therapy;
5. Major surgery or significant trauma due to any reason within 4 weeks before randomization (major surgery is defined as procedures under general anesthesia or involving significant incisions);
6. Active hepatitis B (HBsAg positive and/or HBcAb positive with HBV-DNA >500 IU/mL) or hepatitis C (HCV antibody positive with HCV RNA positive);
7. Concurrent human immunodeficiency virus (HIV) infection;
8. Clinically active infection affecting subject safety, including active pulmonary tuberculosis;
9. Uncontrolled hyperglycemia, acute cholelithiasis, or patients predisposed to pancreatitis acute;
10. Investigator-assessed uncontrolled electrolyte disturbances (e.g., hypocalcemia, hypomagnesemia, hypokalaemia);
11. Subjects unable to swallow;
12. History of extensive diffuse/bilateral interstitial fibrosis, or known Grade 3/4 interstitial fibrosis, or current clinically significant active lung diseases including pulmonary infection, pneumonia allergic, interstitial pneumonia, other interstitial lung diseases, obliterative bronchiolitis, or current radiation pneumonitis requiring steroid treatment (excluding prior history of radiation pneumonitis);
13. Clinically significant vascular (arterial/venous) and non-vascular cardiac diseases within 3 months before randomization, potentially including but not limited to:
    a. Arterial disorders: cerebrovascular accident/stroke (including transient ischaemic attack), myocardial infarction, angina unstable;
    b. Venous disorders: cerebral venous thrombosis, symptomatic pulmonary embolism;
    c. Non-vascular cardiac disorders: cardiac failure congestive (New York Heart Association Classification ≥ II), II/III degree atrioventricular block (excluding those with pacemakers) or atrioventricular block with PR>220 ms; NCI CTCAE grade ≥2 persistent arrhythmia, uncontrolled atrial fibrillation of any grade, bradycardia <50 bpm (unless in athletes), QTc>470 msec on ECG, or congenital long QT syndrome;
    d. Cardiac ultrasound indicates LVEF＜50%;

14. Requirement for concomitant use of QTc-prolonging drugs or medications inducing torsade de pointes ventricular tachycardia within 14 days before randomization or during treatment;
15. Participation in other clinical studies with treatment received within one month before randomization;
16. Uncontrolled hypertension (defined as resting blood pressure systolic ≥150 mmHg and/or blood pressure diastolic ≥95 mmHg);
17. Clinically significant gastrointestinal abnormalities including active colitis ulcerative, chronic diarrhoea due to malabsorption, Crohn's disease, and/or prior surgery affecting absorption;
18. Other severe and/or uncontrolled comorbidities that may affect safety or efficacy assessments as judged by the investigator;
19. Subjects currently receiving potent CYP3A4 inhibitors/inducers that cannot be discontinued within 1 week prior to prior to randomization (or within 5 half-lives of the drug, whichever is longer) and during the study period;
20. Subjects planning to undergo surgery, or whom the investigator considers may require surgery during the study period (affecting subject safety or efficacy evaluation);
21. Subjects with known allergies to Crizotinib Capsules or any component of XZP-3621 Tablets, or those with a history of severe allergies;
22. Female patients during pregnancy or lactation;
23. Subjects with psychiatric history, incapacitated persons, or persons with limited legal capacity;
24. History of alcoholism or drug abuse;
25. Other circumstances deemed by the investigator to be unsuitable for enrollment.

5.3 Other Eligibility Considerations

To assess the impact of subject criteria on safety, the investigator must refer to the Investigator's Manual for specific information such as warnings, precautions, contraindications, AEs, and other important data regarding study treatments in this study.

5.4 Study Screening and Randomization

5.4.1 Study Screening

For subjects entering the screening period, the investigator should keep records. During the screening period (Day -28 to Day -1), the principal investigator or trained investigator will:

1. First obtain the subject's signed informed consent form before performing any study-related operations;

2. Determine whether the subject meets the inclusion criteria and does not meet the exclusion criteria;

3. In a few cases, one re-examination or re-screening of the subject is allowed after communication with the sponsor. The re-screened subject needs to re-sign the informed consent form and re-obtain the screening number. Only one re-examination or re-screening can be performed.

4. To ensure that only subjects who meet the clinical study eligibility are randomly enrolled in the study, a qualification review process will be implemented for all subjects. The study site will complete the study-specific qualification review form and send the form to the sponsor for review and approval before randomization, provided that the process does not violate relevant regulations and ensures that relevant data protection, privacy regulations and relevant regulations are complied with.

5.4.2 Randomization

Subjects who meet the screening criteria will be randomized to the test group and the control group at a 1:1 ratio using the IWRS system with stratified block randomization. Stratification factors are: 1. Brain metastasis (Yes, No), 2. Chemotherapy (Yes, No). Subjects participating in this study will be assigned a unique identification number.

5.5 Handling Procedures for Inappropriately Enrolled Subjects

Subjects who do not meet the eligibility criteria must not be enrolled to receive study treatment under any circumstances. No exceptions to this rule are allowed. If a subject does not meet all eligibility criteria but inadvertently starts study treatment, the investigator should immediately notify the sponsor and discuss with the sponsor whether to continue or terminate the subject's study treatment. The sponsor must ensure that all decisions are accurately documented.

5.6 Subject Discontinuation of Study Treatment

If a subject discontinues treatment before the study is completed, the reason for discontinuing study treatment will be recorded in the electronic case report form (eCRF). These reasons include:

- Radiologically confirmed disease progression (investigator assessment and BICR assessment): If the BICR and the investigator's judgment are inconsistent, the subject may continue treatment with the investigational medicinal product; unless the investigator assesses from a risk-benefit perspective that the subject no longer benefits

from continuing treatment with the investigational medicinal product, treatment may also be discontinued;

- Any AE that leads to the subject being unable to continue participating in the study treatment;
- The investigator or sponsor determines that the subject should discontinue study treatment based on the subject's best interests (e.g., serious non-compliance with inclusion criteria is discovered after participating in the study, the investigator determines that the potential risks of the subject continuing to participate in the study treatment outweigh the potential benefits; clinical signs and symptoms worsen, and the investigator determines that continued medication cannot provide clinical benefit, etc.);
- The subject decides to discontinue the study treatment (subjects who decide to discontinue treatment are generally expected to continue participating in the study unless the subject explicitly withdraws informed consent and refuses to participate in any subsequent study procedures and assessments);
- The subject needs to use drugs contraindicated in this study;
- The subject cannot comply with the study and/or study protocol (such as medication instructions, study visits);
- Subject lost to follow-up;
- Subject pregnancy;
- Subject death;
- The sponsor terminates the study.

Subjects who discontinue treatment need to complete all the tests stipulated at the end-of-treatment visit and the subsequent follow-up as stipulated.

If a subject becomes pregnant while receiving the investigational medicinal product, treatment must be stopped immediately and permanently. Pregnant subjects should complete the end-of-treatment visit and safety follow-up. The study site should complete the pregnancy event report form, notify the sponsor (or its designee), and conduct follow-up observations in accordance with the pregnancy event follow-up content.

In the clinical study, if a subject discontinues treatment, the investigator should report all observations and reasons for withdrawal from the last dose of study medication to withdrawal from the study in as much detail as possible. The date of treatment discontinuation and the reason for treatment discontinuation will be recorded in the subject's original documents and eCRF.

If therapy is interrupted or terminated due to an AE or laboratory test abnormal value, subjects must be followed up and assessed until the AE resolves or stabilizes.

5.7 Subject Withdrawal from the Study

The reasons for a subject's withdrawal from the study will be recorded in the original medical records and eCRF. Possible reasons include:

- Withdrawal of informed consent (informed consent for study treatment and assessment);
- Loss to follow-up;
- Death.

Subjects may withdraw from the study at any time for any reason. Subjects who withdraw their informed consent to participate and disclose information will not receive investigational medicinal product therapy, or undergo any follow-up or collect any subsequent data. The sponsor will retain the data and information obtained prior to the withdrawal of informed consent. If a subject withdraws informed consent to participate in the investigational medicinal product therapy but does not withdraw informed consent for further disclosure of information, the subject's survival-related data will still be collected until the end of the study (if possible).

6. Study Treatment

6.1 Investigational Product

6.1.1 Investigational Medicinal Product

Name:	XZP-3621 Tablet
Active Ingredient:	XZP-3621
Strengths:	250 mg, 50 mg
Description:	The product is film-coated and appears off-white after removal of the coating
Storage:	Store protected from light and sealed at room temperature
Company Name	Simcere Pharmaceutical Co., Ltd.

6.1.2 Control Drug

Name:	Crizotinib Capsules (Xalkori)
Active Ingredient:	Crizotinib
Strengths:	250 mg, 200 mg
Description:	The product is a capsule formulation with contents consisting of white to pale yellow powder
Storage:	Store below 30°C
Company Name	Pfizer Pharmaceuticals Limited

6.2 Management of Investigational Medicinal Product

6.2.1 Drug Management

The study site will record all IMP supplies in accordance with GCP requirements, including detailed information on receipt, storage, dispensing, and return.

The investigator should administer the drug to subjects according to their group in sequence, and accurately record the actual dose taken by each subject, which should be consistent with the required dose. Medication adherence will be determined based on actual medication records.

The principal investigator will authorize a designated person to manage the investigational medicinal product (IMP), including receiving, storing, dispensing, counting, and retrieving. The relevant processes will be recorded and preserved.

All IMPs will be stored according to the storage conditions specified in the above description of the IMP. The area should be secure, temperature-controlled, and accessible only to authorized personnel. Used packaging boxes should be stored and managed in the same manner as the IMP. The storage temperature and other relevant information of the IMPs must be recorded daily. The sponsor reserves the right to request monitoring and auditing of the receiving, storage, dispensing, and retrieval processes of the IMP, as well as related records.

The dispensing and retrieval of drugs at the study site will be conducted by the principal investigator or authorized study personnel. During home treatment, subjects need to record the time and quantity of medication taken. At each follow-up visit, study personnel should accurately record the actual quantity of drugs used and returned by the subjects, and will also dispense the subjects with enough study medication for one follow-up interval.

At the end of the study, all unused IMPs and their packaging will be returned to the sponsor for centralized disposal.

6.2.2 Supply, Packaging, and Labeling

The sponsor will provide a sufficient quantity of IMPs and will store them at room temperature.

Study site staff are responsible for dispensing the IMP to subjects. All IMPs are packaged and labeled by the sponsor in accordance with Chinese laws and regulations and the Good Manufacturing Practice (GMP) guidelines. The label should include the protocol number, sponsor, batch number, storage conditions, dosage and administration, expiration date, and a statement indicating "For clinical research use only," among other information. The receipt and dispensing of drugs at the study site will be managed by personnel authorized by the principal investigator, and this document will be retained.

6.3 Investigational Medicinal Product Dosage and Administration

XZP-3621 Tablets, 500 mg once daily, administered with meals or within 30 minutes after meals, continuously administered with 4-week treatment cycles. It should be swallowed whole, and it is recommended to administer at a relatively fixed time every day.

Crizotinib Capsules, 250 mg orally, twice daily, administered with or without food, continuously administered with 4-week treatment cycles. It should be swallowed whole, and it is recommended to administer at a relatively fixed time every day.

6.3.1 Overdose or Missed Dose of Investigational Medicinal Product

Subjects suspected of investigational medicinal product overdose should have appropriate supportive treatment determined by the investigator in consultation with the sponsor. Any AEs resulting from overdose should be reported to the sponsor.

If the subject experiences vomiting after taking the medicine or misses a dose for any reason, there is no need to make up for the missed dose. Just take the medicine at the next scheduled time at the prescribed dose.

6.4 Dose Adjustment

6.4.1 Dose Adjustment Principles of XZP-3621 Tablets

If ≥ Grade 3 AEs related to XZP-3621 occur, the investigator may consider suspending XZP-3621 treatment while providing active symptomatic treatment according to the specific situation. If treatment is suspended due to toxic reaction, and the toxic reaction recovers or decreases to ≤ Grade 1 after continuous discontinuation for ≤ 7 days, XZP-3621 treatment may be resumed at the original dose level.

If continuous drug interruption >7 days and ≤21 days, and the toxicity reaction recovers to ≤ Grade 1, XZP-3621 treatment will restart from 500 mg down to 400 mg level; if it still does not recover to ≤ Grade 1 after 21 days of continuous drug interruption, the subject must terminate XZP-3621 treatment.

The investigator is allowed to adjust the dose according to the patient's condition, and a total of two dose reductions are allowed. If it is still not tolerated after being reduced to 400 mg in the above situation, continuous drug interruption >7 days and ≤21 days occurs again due to toxicity reaction, and the toxicity reaction recovers to ≤ grade 1, then XZP-3621 can be reduced again to 300 mg. In any other situation, the investigator must discuss with the sponsor's medical department before making a decision.

6.4.2 Dosage Adjustment Principles of Crizotinib Capsule

Dose adjustments for adverse reactions with Crizotinib Capsules should follow the approved package insert. The recommended dose reduction methods are as follows:

- First dose reduction: Oral, 200 mg, twice daily
- Second dose reduction: Oral, 250 mg, once daily
- If 250 mg Crizotinib Capsules orally once daily is still not tolerated, discontinue permanently.

The following is a reference guide for dose reduction of Crizotinib Capsules in the event of adverse reactions:

For Grade 3 hematologic toxicity related to Crizotinib (excluding lymphopenia unless accompanied by clinical events, e.g., opportunistic infection), interrupt dosing until recovery to ≤ Grade 2 or baseline, then resume at the same dose; for Grade 4 hematologic toxicity related to Crizotinib (excluding lymphopenia unless accompanied by clinical events, e.g., opportunistic

infection), suspend dosing until recovery to ≤ Grade 2 or baseline, then resume at the next lower dose;

For Crizotinib-related alanine aminotransferase (ALT) or aspartate aminotransferase (AST) greater than 5 times the upper limit of normal (ULN) with total bilirubin less than or equal to 1.5 times the ULN, suspend dosing until recovery to baseline or ≤ 3 times the ULN, then resume at the next lower dose;

For Crizotinib-related ALT or AST greater than 3 times the ULN, with total bilirubin greater than 1.5 times the ULN (in the absence of cholestasis or hemolysis), permanently discontinue Crizotinib Capsules;

For any grade of interstitial lung disease/noninfectious pneumonitis related to Crizotinib, permanently discontinue Crizotinib Capsules;

If at least 2 separate electrocardiogram (ECG) findings related to Crizotinib show QT corrected for heart rate (QTc) > 500 ms, suspend dosing until recovery to baseline levels or QTc < 481 ms, and continue dosing at the next lower dose. If QTc > 500 ms or change from baseline is greater than or equal to 60 ms, accompanied by symptoms/signs of *torsade de pointes*, polymorphic ventricular tachycardia, or severe arrhythmia, permanently discontinue Crizotinib Capsules treatment;

If Crizotinib-related bradycardia (symptomatic, potentially serious, medically significant, requiring medical intervention) occurs, suspend dosing until recovery to asymptomatic bradycardia or heart rate of 60 bpm or higher. If bradycardia (life-threatening, requiring emergency intervention) occurs, permanently discontinue the drug if no concomitant medications are identified as causing bradycardia;

In the event of Crizotinib-related vision loss (Grade 4 visual impairment), discontinue the drug while evaluating severe vision loss.

6.5 Concomitant Therapies

All medications used within 6 weeks prior to the first dose are considered prior medications and will be recorded in the original documents and eCRF.

Investigators should instruct subjects to report to the study site any new medications (including prescription and all over-the-counter medications) used since first receiving IMP therapy. All medications (excluding the IMP) and significant non-drug therapies (including physical therapy and transfusion) administered from the first dose of the IMP up to 30 days after the last dose of the IMP are considered concomitant therapies and will be recorded in the subject's original documents and eCRF. The minerals or vitamins taken should also be recorded. Each concomitant medication or important non-drug therapy should be recorded. If prior medications are continued during the study, they need to be recorded as concomitant medications.

6.5.1 Prohibited Concomitant Therapies

6.5.1.1 XZP-3621 Tablets

During the study treatment, no other anti-tumor treatments are allowed (including chemotherapy, radiotherapy, immunotherapy, molecularly targeted therapy, and traditional Chinese medicine, etc., except for palliative radiation for local pain caused by metastases to bone).

During the study treatment, no other investigational products may be used, and subjects are not allowed to participate in any other clinical studies in any therapeutic area (non-interventional studies may be allowed with the consent of the investigator and the sponsor).

CYP3A4 is the main metabolic enzyme for the metabolism and elimination of XZP-3621. Therefore, potent CYP3A4 inhibitors and/or inducers should be prohibited in food or drugs within 14 days before the first dose of XZP-3621 and throughout the study, as shown in Table 6-1 and Table 6-2.

Table 6-1 Some Potent CYP3A4 Inhibitors and Inducers

Potent CYP3A4 Inhibitors	Potent CYP3A4 Inducers
• Anti-HIV drugs: Amprenavir, atazanavir, fosamprenavir, indinavir, lopinavir, nelfinavir, ritonavir, saquinavir, delavirdine<br>• Anti-HCV drugs: Boceprevir, telaprevir<br>• Dual V1a and V2 receptor antagonist of arginine vasopressin (AVP): Conivaptan<br>• Circulatory system drugs: Mibefradil, verapamil, diltiazem<br>• Antidepressant: Nefazodone<br>• Antifungals: Itraconazole, voriconazole, ketoconazole, miconazole, posaconazole<br>• Antibacterials: Clarithromycin, erythromycin, telithromycin<br>• Foods and beverages: Grapefruit, grapefruit juice	• Psychotropic drugs: Phenobarbital, primidone, carbamazepine, felbamate, phenytoin<br>• Anti-HIV drug: Nevirapine<br>• Antitubercular drugs: Rifabutin, rifampicin, rifapentine<br>• Antidepressant: St. John's Wort

After a single oral administration of 20 mg/kg of XZP-3621 to cynomolgus monkeys, male animals showed a slight QTc interval prolongation (<10%, with no toxicological significance). Therefore, concomitant medications that increase or potentially increase the risk of QTc interval prolongation should be avoided during XZP-3621 treatment.

Table 6-2 Concomitant Medications That May Cause QT Interval Prolongation

Classification	Example
Antiarrhythmics	Amiodarone, procainamide, disopyramide, quinidine, dofetilide, sotalol, ibutilide, bepridil, dronedarone, flecainide

Antipsychotics	Chlorpromazine, haloperidol, thioridazine, droperidol, levomepromazine, levosulpiride, mesoridazine, pimozide, sulpiride, sultopride
Antibacterials	Azithromycin, ciprofloxacin, levofloxacin, clarithromycin, moxifloxacin, erythromycin, sparfloxacin, gatifloxacin, grepafloxacin, roxithromycin
Antifungals	Fluconazole
Antidepressants	Citalopram, escitalopram
Antiemetics	Ondansetron
Antithrombotics	Anagrelide, cilostazol
Antiallergics	Astemizole, terfenadine
Gastrointestinal Motility Agents	Cisapride, Domperidone
Anesthetics	Cocaine, propofol, sevoflurane
Drugs for Haemorrhage Varices Oesophageal	Terlipressin
Drugs for Alzheimer's disease	Donepezil
Drugs for Relaxing Vascular Smooth Muscle	Papaverine
Drugs for Hypercholesterolaemia	Probucol
Drugs for Pollakiuria, Micturition Urgency, Urinary Incontinence, etc.	Terodiline

For the most up-to-date list, please refer to:
https://www.crediblemeds.org/everyone/composite-list-all-qtdrugs/

Treatment should be based on the subject's best interests, considering its necessity (e.g., treatment required for concomitant chronic disease/condition or life-threatening illness), and should be discussed with the sponsor's medical staff when necessary.

6.5.1.2 Crizotinib Capsules

The prohibited concomitant therapies during treatment with Crizotinib Capsules should follow the approved package insert, as detailed below:

Potent or Moderate CYP3A Inhibitors

Co-administration of Crizotinib with potent CYP3A inhibitors increases Crizotinib plasma concentrations, which may increase the risk of adverse reactions to Crizotinib Capsules. Concomitant use of the following potent CYP3A inhibitors should be avoided (including but not limited to): clarithromycin, indinavir, itraconazole, ketoconazole, nefazodone, nelfinavir, ritonavir, saquinavir, troleandomycin, voriconazole, grapefruit or grapefruit juice. If concomitant use of potent CYP3A inhibitors cannot be avoided, the dose of Crizotinib Capsules should be reduced. Caution should be exercised with concomitant use of moderate CYP3A inhibitors.

Potent CYP3A Inducers

Co-administration of Crizotinib with potent CYP3A inducers decreases Crizotinib plasma concentrations, which may reduce the efficacy of Crizotinib Capsules. Concomitant use of potent CYP3A inducers including, but not limited to: carbamazepine, phenobarbital, phenytoin, rifampin, rifabutin and St. John's Wort, should be avoided.

CYP3A Substrates

Co-administration of Crizotinib with CYP3A substrates increases plasma concentrations of the CYP3A substrates, which may increase the risk of adverse reactions to these substrates. Concomitant use of Crizotinib Capsules with CYP3A substrates including, but not limited to: alfentanil, cyclosporine, dihydroergotamine, ergotamine, fentanyl, pimozide, quinidine, sirolimus and tacrolimus, should be avoided since minimal concentration changes may lead to serious adverse reactions. If concomitant use of Crizotinib Capsules cannot be avoided, doses of these CYP3A substrates should be reduced according to their approved product labeling.

Drugs that Prolong the QT Interval

Crizotinib Capsules prolong the QT/QTc interval. Concomitant use of Crizotinib Capsules with drugs known to prolong the QT interval should be avoided.

Drugs that Cause Bradycardia

Crizotinib Capsules can cause bradycardia. Concomitant use of Crizotinib Capsules with medicinal products known to cause bradycardia (e.g. beta blockers, non-dihydropyridine calcium channel blockers, clonidine and digoxin) should be avoided.

6.5.2 Permitted Concomitant Therapies

In consideration of the subjects' interests, necessary supportive treatment for adverse reactions occurring in the subjects is allowed during the study treatment. Supportive treatments include: palliative radiotherapy for local pain caused by metastases to bone, analgesics, antiemetics, medication for constipation or diarrhoea or rash, transfusion, granulocyte colony-stimulating factor (GCSF) and other bone marrow hematopoietic growth factors, bisphosphonates, etc.

For the following AEs, the following medications are recommended, and investigators may give priority to them when prescribing medications:

- Recommended drugs and usage for nausea, vomiting, and diarrhoea (see Appendix 7 for details);
- For fungal infection, echinocandin drugs (such as micafungin, caspofungin) or amphotericin B are recommended;
- For bacterial infection, β-lactams, carbapenems, and cephalosporin antibiotics are

recommended;

- Lorazepam and alprazolam are recommended for subjects with anxiety;

6.5.3 Concomitant Therapies to Be Used with Caution

Investigational medicinal product: Preclinical data show that XZP-3621 is conducive to dissolution in an acidic gastrointestinal environment. During the administration of XZP-3621, long-term (e.g., more than 1 month) use of proton pump inhibitors should be avoided as much as possible. These drugs can efficiently and rapidly inhibit gastric acid secretion, which may affect the dissolution and absorption of the drug, thereby affecting the efficacy. Clinically commonly used proton pump inhibitors (PPIs) include omeprazole, pantoprazole, lansoprazole, rabeprazole, esomeprazole, etc.

Control drug: Concomitant therapies to be used with caution for Crizotinib Capsules should follow the approved package insert.

6.6 Treatment Compliance

The investigator must instruct the subject to strictly follow the protocol to receive the study treatment and explain that compliance is necessary to ensure the safety of the subject and the validity of the study, in order to improve compliance. In addition, the subject must be instructed to contact the investigator if he/she is unable to receive the study treatment as prescribed for any reason.

If the medication is taken outside the hospital, the subject is required to bring the medication package (whether or not there is any remaining medication) dispensed at the last follow-up to the study site for each visit to the investigator. All investigational medicinal products dispensed and returned must be documented in the original documents; if the medication is taken in the hospital, the date, time, and dosage of each investigational medicinal product administration must be recorded. These detailed records will be used to demonstrate treatment compliance. A subject is considered compliant with investigational medicinal product therapy if he/she does not miss more than 20% of the expected investigational medicinal product administration within the time range from the first dose to the last dose.

7. Study Procedure

7.1 Visit Procedures

7.1.1 Screening Visit (D-28~ D-1)

The investigator should obtain the subject's written informed consent before conducting screening assessments. The following procedures need to be completed during the screening period:

- Informed consent;
- Verification of inclusion/exclusion criteria;
- Demographic data;
- Non-tumor medical history;
- Tumor diagnosis and treatment history;
- Vital signs;
- Physical examination;
- ECOG score;
- Glycosylated haemoglobin (HbA1C);
- Hepatitis B five items, HCV, HIV testing;
- Blood pregnancy test;
- Hematology;
- Blood chemistry;
- Coagulation function;
- Urinalysis;
- 12-lead ECG;
- Echocardiography;
- Tumor imaging examinations and tumor assessments (RECIST1.1 and RANO-BM criteria);
- Tumor tissue sample (ALK mutation detection);
- Recording of AEs;
- Recording of concomitant medications.

7.1.2 Treatment Period Visits (C1-Cn)

7.1.2.1 Cycle 1 Day 1 (C1D1)

Subjects need to undergo tests and examinations on Cycle 1 Day 1 (C1D1). The following procedures should be completed during this visit:

- Verification of inclusion/exclusion criteria;
- Randomization;
- Vital signs;
- Physical examination;
- ECOG score;
- Blood pregnancy test
- Hematology;
- Blood chemistry;
- Coagulation function;
- Urinalysis;
- 12-lead ECG;
- PK blood collection;
- Administration;
- Recording of AEs;
- Recording of prior and concomitant therapies;
- Patient-reported outcomes (PRO).

7.1.2.2 Cycle 1 Day 15 (C1D15)

Subjects need to have a visit on Cycle 1 Day 15 (C1D15). The following procedures should be completed during this visit:

- Vital signs;
- Physical examination;
- ECOG score;
- Hematology;
- Blood chemistry;
- 12-lead ECG;

- PK blood collection;
- Administration;
- Recording of AEs;
- Recording of prior and concomitant medications.

7.1.2.3 Cycle 2 Day 1 (C2D1)

Subjects need to undergo tests and examinations on Cycle 2 Day 1 (C2D1). The following procedures should be completed during this visit:

- Vital signs;
- Physical examination;
- ECOG score;
- Hematology;
- Blood chemistry;
- Coagulation function test;
- Urinalysis;
- 12-lead ECG;
- PK blood collection;
- Administration;
- Recording of AEs;
- Recording of prior and concomitant therapies;
- Patient-reported outcomes (PRO).

7.1.2.4 Cycle 3 Day 1 (C3D1)

Subjects need to undergo tests and examinations on Cycle 3 Day 1 (C3D1). The following procedures should be completed during this visit:

- Vital signs;
- Physical examination;
- ECOG score;
- Hematology;
- Blood chemistry;
- Coagulation function test;

- Urinalysis;
- 12-lead ECG;
- PK blood collection;
- Tumor imaging examinations and tumor assessments (RECIST1.1 and RANO-BM criteria);
- Administration;
- Recording of AEs;
- Recording of prior and concomitant therapies;
- Patient-reported outcomes (PRO).

7.1.2.5 Cycle 4 Day 1 (C4D1)

Subjects need to undergo tests and examinations on Cycle 4 Day 1 (C4D1). The following procedures should be completed during this visit:

- Vital signs;
- Physical examination;
- ECOG score;
- Glycosylated haemoglobin (HbA1C);
- Hematology;
- Blood chemistry;
- Coagulation function test;
- Urinalysis;
- 12-lead ECG;
- PK blood collection;
- Administration;
- Recording of AEs;
- Recording of prior and concomitant therapies;
- Patient-reported outcomes (PRO).

7.1.2.6 Cycle n Day 1 (CnD1, n≥5)

Subjects need to undergo tests and examinations on Cycle n Day 1 (CnD1). The following procedures should be completed during this visit:

- Vital signs;
- Physical examination;
- ECOG score;
- Glycosylated haemoglobin (HbA1C);
- Hematology;
- Blood chemistry;
- Coagulation function test;
- Urinalysis;
- 12-lead ECG;
- Tumor imaging examinations and tumor assessments (RECIST1.1 and RANO-BM criteria):
- Administration;
- Recording of AEs;
- Recording of prior and concomitant therapies;
- Patient-reported outcomes (PRO).

7.1.3 End-of-Treatment (EOT) Visit

Subjects need to have an EOT visit within 14 days after the investigator decides that the subjects withdraw from the study, and the following procedures should be completed during this visit:

- Vital signs;
- Physical examination;
- ECOG score;
- Glycosylated haemoglobin (HbA1C);
- Blood pregnancy test;
- Hematology;
- Blood chemistry;
- Coagulation function test;
- Urinalysis;
- 12-lead ECG;

- Echocardiography;
- Tumor imaging examinations and tumor assessments (RECIST1.1 and RANO-BM criteria);
- Recording of AEs;
- Recording of prior and concomitant therapies;
- Patient-reported outcomes (PRO).

7.1.4 Safety Follow-up (30 days after the last dose)

The study site will contact the subject by phone or recall subjects to the study site within 30 days after the last dose of investigational medicinal product for a safety follow-up visit to understand the resolution status of ongoing AEs or laboratory test abnormal changes, and to assess new AEs, SAEs, the use of new concomitant medications/subsequent anti-tumor therapies, or changes in previous concomitant medications that occurred from the last assessment to 30 days after the last dose. Any new events occurring during the safety follow-up period need to be recorded in the eCRF.

7.1.5 Survival Follow-up (every 12 weeks after the last dose)

The study site will contact subjects by phone or recall subjects to the study site every 12 weeks after the last dose for survival follow-up to understand the survival information and subsequent anti-tumor treatment. Survival follow-up will continue until the subject's death, loss to follow-up, or withdrawal of informed consent (whichever occurs first). After stopping investigational medicinal product therapy for any reason (other than death, disease progression, loss to follow-up, study termination, or withdrawal of informed consent), subjects will continue to undergo tumor assessments at the time points specified in the protocol during the follow-up period until disease progression or initiation of new anti-tumor therapy.

7.2 PK Blood Sampling Procedure

Refer to the laboratory manual for PK testing for details. PK blood sampling time points and blood volume are shown in Table 7-1.

Table 7-1 PK Blood Sampling Time Points and Blood Volume

Blood Sampling	Time Point (min)	Sample Size	Time Window (min)	PK Blood Volume (mL)
**Pre-dose on C1D1**	/	1	-60~0	4
**Post-dose on C1D1**	240	1	-90/+90	4
**Pre-dose on C1D15**	/	1	-60~0	4
**Pre-dose on C2D1**	/	1	-60~0	4
**Pre-dose on C3D1**	/	1	-60~0	4
**Pre-dose on C4D1**	/	1	-60~0	4
**Total**		**Approximately 6 samples**		**Approximately 24 mL**

7.3 Sample Collection, Storage and Destruction

In this study, the collection, gathering, transportation, preservation, detection, and destruction of plasma and tissue samples from patients involved in the testing will all be conducted within China, with no plans to export or send any samples abroad. Data will be analyzed within China, as detailed in the bioanalytical testing laboratory manual or operating manual.

8. Study Assessment

8.1 Safety Assessment

As shown in the flowchart, a safety assessment is required at each follow-up visit.

- AE assessment (graded according to CTC AE v5.0 criteria);
- Physical examination and vital signs;
- 12-lead ECG and echocardiography, etc.;
- Laboratory tests (hematology, blood chemistry (fasting), coagulation function, urinalysis, etc.).

8.1.1 Vital Signs

Vital signs examination will be performed according to the study flow chart.

It is recommended that subjects should have their seated blood pressure, respiratory rate, pulse, and body temperature measured after resting for at least 5 min.

If the scheduled time for vital sign measurement coincides with the time for blood sample collection for laboratory tests, the vital signs should be measured before the laboratory test sample collection or after resting for more than 10 min after blood collection.

Vital signs results should be recorded in the eCRF. If an investigator considers an examination result to be abnormal and clinically significant, it is considered an AE.

8.1.2 Physical Examination

The investigator will conduct physical examination according to the study procedure. The examination parameters include: body height (screening period only), weight, general condition, skin and mucous membranes, superficial lymph nodes throughout the body, head and its organs, neck, chest, abdomen, rectum, anus and external genitalia, spine and limbs, and nervous system.

The results of the physical examination should be recorded in the eCRF. If an investigator considers an examination result to be abnormal and clinically significant, it is considered an AE.

8.1.3 Echocardiography

Echocardiography will be performed according to the study procedure to monitor LVEF, and LVEF will be recorded in the eCRF. If LVEF decreases, the investigator will increase the frequency of echocardiography assessments as needed based on the patient's condition. If clinically significant changes from baseline occur, they should be recorded as AEs.

8.1.4 Electrocardiogram

A 12-lead ECG examination will be performed according to the study procedure. Subjects need to undergo ECG examination in a resting state and remain supine but awake when collecting the electrocardiogram.

An ECG examination is required at each visit. If there are clinical symptoms, an ECG examination can be performed at other times. The investigator shall determine whether to perform additional 12-lead ECG examinations based on the subject's condition or if clinical symptoms occur.

The following parameters should be recorded: HR, PR interval, RR interval, QRS duration, QT interval, QTc, QT interval corrected for heart rate using Fridericia's formula [QTcF], and any abnormal value assessments. The investigator should record the results of the 12-lead ECG in the eCRF (i.e., normal, abnormal but not clinically significant, or abnormal and clinically significant). Clinically significant abnormalities will be reported as AEs. Measurements may be repeated if needed.

A significant QTcF interval prolongation is defined as a single QTcF interval ≥500 msec or a single QTcF interval increase from baseline ≥60 msec. If QTcF prolongation occurs during the study, the use of the investigational product should be suspended, an electrocardiogram should be repeated, and (or if the investigator deems it necessary) a consultation with cardiologists should be requested. If the cardiologists confirm that the subject has a significant QTcF interval prolongation:

- The use of the IMP will be discontinued;
- The subject will undergo medical evaluation, appropriate treatment, and at least three ECG examinations per week until the QTcF interval is <480 msec;
- Treatment may be resumed only with the investigator's consent.

8.1.5 Laboratory Safety Test

Laboratory tests include hepatitis B five items, HCV, HIV, hematology, blood chemistry, coagulation function, glycosylated haemoglobin (HbA1c), blood human chorionic gonadotropin (HCG) and urinalysis. See Appendix 1 for details.

Blood and urine samples for clinical laboratory assessment will be collected according to the protocol. See Section 14.1 for specific items. For unexplained or unexpected laboratory test abnormal values that are deemed clinically significant by the investigator, repeat testing is recommended within 24 hours and continued follow-up until the test results return to the normal range and/or the cause of the abnormality is confirmed. Laboratory test result abnormalities that meet one or more of the following criteria should be recorded as adverse events: 1. Clinically

significant or accompanied by clinical symptoms; Leading to adjustments in study treatment (i.e., dose adjustment, temporary interruption, or permanent discontinuation); Requiring subjects to change concomitant medications (i.e., adding, interrupting, or discontinuing concomitant drugs or treatments, or making any other changes to concomitant drugs or treatments). Clinical terms should be used whenever possible instead of laboratory test terms (e.g., anaemia, rather than low haemoglobin value).

8.2 Efficacy Assessment

According to the Response Evaluation Criteria in Solid Tumors (RECIST) version 1.1 (see Appendix 4) and RANO-BM criteria (see Appendix 5), imaging examination should be performed, and the efficacy should be evaluated separately by the investigator and the independent central imaging. Each subject should undergo imaging examination and assessment of tumor lesions during the screening period, treatment period, and EOT visit. Enhanced CT scans of the chest, abdomen, pelvis, and any other sites suspected of having tumor lesions, head MRI, and bone scan should be performed during the screening period. For those with contrast media allergy, plain CT or MRI can be used. For diagnostic or staging needs, the investigator may decide whether to perform additional imaging examinations (e.g., PET/CT or bone scan) to detect other potential tumor lesions. CT and MRI examination results completed within 28 days prior to randomization are acceptable; bone scan results within 3 months before randomization are acceptable. Subsequent visits should include a bone scan when clinically indicated (e.g., suspected metastases to bone or exacerbation). It should be noted that the examination methods should remain consistent for each subject throughout the study.

For diagnostic or staging needs, the investigator may decide whether to perform additional imaging examinations (e.g., PET/CT or bone scan) to detect other potential tumor lesions. Imaging examinations will be performed every 8 weeks for the first 64 weeks of the treatment period, and every 12 weeks thereafter, with an imaging examination of all tumor lesions performed at the EOT visit (except for those who have imaging examinations of all tumor lesions within 4 weeks). Unscheduled imaging examinations may also be added if disease progression is suspected. After stopping investigational medicinal product therapy for any reason (other than death, disease progression, loss to follow-up, study termination, or withdrawal of informed consent), subjects will continue to undergo tumor assessments at the time points specified in the protocol during the follow-up period until disease progression or initiation of new anti-tumor therapy.

This study plans to use Blinded Independent Central Review (BICR). The purpose of the independent central imaging assessment is to provide a fair, objective, and less variable third-party efficacy assessment of all subjects' related medical imaging data in this study. The

assessment will be performed according to the Response Evaluation Criteria in Solid Tumors (RECIST 1.1 and RANO-BM criteria).

The BICR will perform independent medical image analysis, reading and evaluation of data generated at baseline and all subsequent time points for each subject, including CT data, MRI data, bone scans, relevant additional imaging data (if applicable), and electronic photographs of skin lesions (if applicable).

See the Independent Review Committee charter for specific operational details.

8.3 Pharmacokinetic Assessment

Population Pharmacokinetic Evaluation:

All subjects in the test group will participate in population pharmacokinetic evaluation. PK blood samples should be collected from subjects before dosing on C1D1, 4 h after dosing on the same day, and before dosing on C1D15, C2D1, C3D1, and C4D1 (patients are required to take medication at the study site) for concentration detection and population pharmacokinetic analysis of XZP-3621 Tablets (XZP-3621 and its main metabolites). If the patient withdraws/terminates study treatment during this period, PK samples should be collected as much as possible. One tube of blood sample (approximately 4 mL per tube) will be collected at any of the above time points. PK blood collection is not required for subjects in the control group.

Refer to the laboratory manual for specific procedures regarding sample collection, handling, storage, transportation, and analysis.

8.4 Patient-Reported Outcomes

Patient-Reported Outcomes (PRO): Paper versions of the EORTC QLQ-C30, EORTC QLQ-LC13, and EQ-5D-5L questionnaires will be completed by all subjects at each visit before any examination. Investigators should ensure the completeness of all questionnaires. Assessments are required every 4 weeks until the end-of-treatment visit. If a subject has been assessed within 7 days prior to the end-of-treatment visit, PRO assessments do not need to be repeated at this visit. PRO assessments should adhere to the time window of the visit in question.

9. Adverse Event

9.1 Definition of Adverse Event

An AE refers to any untoward medical occurrence in a subject after administration of the investigational product, which does not necessarily have a causal relationship with the investigational product. Therefore, an AE can be any unfavorable and unintended sign (including abnormal laboratory/ancillary examination finding), symptom, or disease that is temporally associated with the use of an investigational product, whether or not considered related to the investigational product.

The natural progression or worsening of neoplasm malignant during the study (including the appearance of new metastasis sites) will be recorded as part of the efficacy evaluation, rather than reported as an adverse event/serious adverse event.

Symptoms and signs clearly related to the study disease during the study should not be reported as AEs unless the investigator determines that the worsening of these symptoms and signs is not solely caused by the natural progression of the study disease, or the investigator believes that the worsening of these symptoms and signs is directly caused by the investigational product. If it is uncertain whether an AE in the study is entirely caused by the disease, the event should be reported as an adverse event or serious adverse event, as appropriate.

For abnormal test results, whether the abnormal objective test value should be reported as an AE should be judged according to the following criteria:

The test result is accompanied by symptoms, and/or;

The test result requires further diagnostic test or drug/surgery intervention, and/or;

The test result leads to adjustment of study medication (beyond the dose adjustment specified in the protocol), and/or;

The investigator or sponsor believes that the test result constitutes an AE.

If none of the above criteria are met, the existence of repeated abnormal tests alone does not constitute an AE. Reporting erroneous test results does not need to be reported as an AE. If a clinical diagnosis has been reported as an AE, the corresponding abnormal laboratory results will not be reported as a separate AE.

Medical conditions, clinically significant signs and symptoms (including laboratory and auxiliary examination abnormal results found during the initial screening examination) that existed prior to the screening period will be recorded as medical history or concomitant illness during the baseline period, rather than as AEs, if there is no worsening in severity or increase in frequency during the study.

Clinical medical events that occur after signing the informed consent form but before the first dose will be recorded as medical history/concomitant illness and not as AEs, unless one of the following applies:

- Any injury/damage caused by clinical laboratory test procedures;
- Adverse events caused by drug discontinuation related to the study protocol;
- Adverse events caused by drugs other than the investigational medicinal product taken as part of the treatment regimen.

9.2 Serious Adverse Event

9.2.1 Definition of Serious Adverse Event

A serious adverse event (SAE) is any untoward medical occurrence (at any dose) that:

- Results in death;
- Is life-threatening (Note: The definition of "life-threatening" in serious adverse event refers to the patient being at immediate risk of death at the time of the adverse event, not assuming that it may lead to death if it develops severely in the future.);
- Results in hospitalization or prolongation of existing hospitalization;
- Results in persistent or significant disability/incapacity;
- Is a congenital anomaly/birth defect;
- Important medical events: Medical and scientific judgment should be exercised in deciding whether expedited reporting is required in other situations, such as important medical events that may not be immediately life-threatening or result in death or hospitalization but may jeopardize the patient or require intervention to prevent one of the outcomes listed above. These should also usually be considered serious.

The following hospitalizations should not be considered SAEs:

- Observation in the emergency room or other hospital department for no more than 24 hours that does not result in hospitalization (unless considered an important medical event or life-threatening);
- Elective surgery that was planned before signing the informed consent form;
- Hospitalization required for routine health examinations (e.g., routine colonoscopy);
- Hospitalization not for the purpose of disease treatment, and planned before enrollment, requiring documented evidence;

- Admission unrelated to health status and not requiring medical/surgery intervention (e.g., family reasons).

9.2.2 Definition of Suspected Unexpected Serious Adverse Reaction

Suspected Unexpected Serious Adverse Reaction (SUSAR) refers to a suspected and unexpected serious adverse reaction where the nature and severity of the clinical manifestation exceeds the existing information in the Investigator's Brochure for the investigational medicinal product, the package insert for marketed drugs, or the summary of product characteristics. The Investigator's Brochure serves as the primary document providing safety reference information for determining whether an adverse event is expected or unexpected.

9.2.3 Disease Progression and New Primary Tumor

Significant events resulting from tumor disease progression are not recorded as SAEs. However, according to NCI CTCAE v5.0, death due to disease progression occurring during the study or during the safety follow-up period after the last dose of investigational medicinal product as specified in the study protocol must be reported as an SAE.

New primary tumors should be classified as SAEs. New primary tumors refer to tumors that are not the primary reason for using the study treatment drug and are discovered after the subject is enrolled in the study.

9.2.4 Abnormal Liver Function Test

Elevations in liver biochemistry indicators that meet Hy's Law are defined and reported as SAEs, and if no other applicable criteria exist, the important medical event is used as the criterion for determining an SAE.

In this study, the following situations should also be reported as SAEs:

Subjects with normal baseline AST or ALT and total bilirubin values: AST or ALT $\geq 3 \times$ ULN and total bilirubin $\geq 2 \times$ ULN during treatment, with no evidence of hemolysis and alkaline phosphatase $\geq 2 \times$ ULN (or alkaline phosphatase value unknown);

Subjects with abnormal baseline ALT or AST or total bilirubin values: AST or ALT $\geq 2 \times$ the baseline value and $\geq 3 \times$ ULN, or $\geq 8 \times$ ULN (whichever is lower), and total bilirubin increased by at least $1 \times$ ULN compared to the baseline value, or total bilirubin value $\geq 3 \times$ ULN (whichever is lower) during treatment.

If a subject experiences the above AST and/or ALT abnormalities concurrent with abnormal elevation of total bilirubin during treatment or during the safety follow-up period, they should return to the study site for evaluation as soon as possible after learning of the abnormal results.

The evaluation should include laboratory test, detailed medical history inquiry, and physical examination, and the possibility of liver tumor (primary or secondary) should be considered.

9.3 Collection and Recording of AEs

AEs will be monitored throughout the study, and investigators are responsible for recording all AEs observed during the study. From the time a subject signs the informed consent form until 30 days after the last dose, all AEs, regardless of severity and causality with the IMP, should be recorded by the investigator in the corresponding AE page in the eCRF (for subjects who start other anti-tumor therapies within 30 days after the last dose, newly occurring AEs after starting other anti-tumor therapies will not be collected).

All AEs must be recorded in detail with the following information:

- AE Description: Describe the AE using medical terminology, rather than the verbatim language reported by the subject. If applicable, it should include symptoms, signs, abnormal laboratory test findings, and diagnoses. If the same AE occurs more than once in the same subject, and the subject has recovered between the two events, the two AEs should be recorded separately.
- AE Start date: The date when the subject first experiences the AE or AE-related symptoms. If the AE is a clinically significant abnormal laboratory test or auxiliary examination result, the start date is the laboratory sampling date or auxiliary examination date when the clinically significant abnormality first appeared in the test value. For pre-existing abnormal conditions in the medical history, the AE start date is recorded as the starting date of clinically significant exacerbation after using the IMP (e.g., the starting time of increased CTCAE severity grade).
- AE End Date: The date when the AE or AE-related symptoms cease. If the AE is an abnormality in laboratory tests or auxiliary examination results, the end date is the earliest date on which the test value decreases to a level that the investigator determines to be of no clinical significance or returns to normal. If the AE outcome is death, the end date is the date of death. If the AE is still present, the end date may not be filled in.
- Severity of AEs.
- Causality of the AE to the IMP.
- Actions taken to the IMP for AE: including no actions taken, dose reduction, temporary drug interruption, permanent drug discontinuation, not applicable.
- Actions taken to subjects: including no actions taken, use of concomitant drug therapy, use of non-drug therapy, other.

- AE outcome: recovered/resolved (without sequelae), recovered/resolved (with sequelae), improved, not improved, death, unknown. Ongoing AEs after a subject completes the study: If follow-up is not possible for any reason, it will be explained in the original medical record. If necessary, the results of laboratory or other tests repeated due to AEs need to be recorded until the AEs return to normal or remain stable or subject death.
- Death: All death events during the study should be collected and recorded in the original record and the death record page of the eCRF, but with death as an outcome rather than an AE name. The AE name should be the medical event that leads to death. If the cause of death is unknown, the AE name should be recorded as "death of unknown cause."

9.3.1 Severity of AEs

When filling out the AE table in the case report form, the investigator will use the Common Terminology Criteria for Adverse Events (CTCAE version 5.0) to determine the severity of the AEs. The specific grading is as follows, and AEs not listed in the CTCAE should also be graded according to the table below:

- **Grade 1:** Mild; asymptomatic or mild symptoms; clinical or diagnostic observations only; intervention not indicated.
- **Grade 2:** Moderate; minimal, local, or noninvasive intervention indicated; limiting age-appropriate instrumental activities of daily living※.
- **Grade 3:** Severe or medically significant but not immediately life-threatening; hospitalization or prolongation of hospitalization indicated; disabling; limiting self-care activities of daily living※※.
- **Grade 4:** Life-threatening consequences; urgent intervention indicated.
- **Grade 5:** Death related to AE.

Note: Activities of Daily Living (ADL)

※: Instrumental ADL refers to preparing meals, shopping for groceries or clothes, using the telephone, managing finances, etc.

※※: Self-care ADL refers to bathing, dressing and undressing, feeding self, using the toilet, taking medications, and not bedridden.

9.3.2 Causality Assessment

The investigator will determine the causality of AEs to IMP according to the following criteria:

Definitely related:	AE occurrence follows a reasonable temporal relationship with IMP use; AE represents known adverse reaction of IMP; AE diminishes/resolves after discontinuation and reappears upon rechallenge; cannot be explained by subject's underlying disease.
Probably related:	AE occurrence follows a reasonable temporal relationship with IMP use; AE represents known adverse reaction of IMP; AE diminishes/resolves after discontinuation (with unknown effect upon rechallenge); cannot be explained by subject's underlying disease.
Possibly related:	AE occurrence follows a reasonable temporal relationship with IMP use; AE represents known/suspected adverse reaction of IMP, but other factors may contribute (e.g., disease, concomitant medications); post-discontinuation effects unclear or lack definitive information.
Unlikely related:	AE occurrence follows a reasonable temporal relationship with IMP use, but the event is not among known drug adverse reactions and most likely caused by subject's disease/other treatments.
Not related:	AE occurrence lacks a reasonable temporal relationship with IMP use (e.g., occurred before drug administration); not among known drug adverse reactions; or AE clearly caused by other factors (e.g., subject's disease, other treatments, or concomitant medications).

9.4 Reporting of SAEs

According to the relevant provisions of the current GCP (2020 edition) in China, the investigator should fill out the SAE report form provided by the sponsor within 24 hours after learning of the SAE or related new follow-up information, and report it in writing to the sponsor or the sponsor's representative (such as a contract research organization (CRO)).

The sponsor or its representative (such as a CRO) is responsible for safety monitoring throughout the study and will ensure that the study site completes the reporting of all relevant safety information in accordance with the requirements of regulatory agencies and local regulations. At the same time, the sponsor or its representative (such as a CRO) must report relevant safety information to the appropriate regulatory authorities in accordance with the requirements of regulatory agencies and local regulations.

To ensure patient safety, all SAEs that occur after the patient signs the informed consent form and up to 30 days after the last dose (safety follow-up period) should be reported in accordance with the above requirements. Relapses, complications, and exacerbations of previously reported SAEs need to be reported to the sponsor as follow-up reports to the original SAE report. The follow-up reporting process and time limit should be implemented in accordance with the SAE reporting process. After the safety follow-up period, if the investigator becomes aware of any new SAE in a subject, and the SAE may be related to the study treatment, the investigator should report it to the sponsor within 24 hours of becoming aware of it.

9.5 Follow-up of AEs

When an AE occurs, the investigator should actively take appropriate measures to ensure the safety of the subjects. All AEs should be followed up until the event resolves or stabilizes, or the event can be explained, the subject is lost to follow-up, or death occurs. If further follow-up information cannot be obtained for some reason before the end of the study, an explanation must be provided in the original medical record.

The investigator can choose various follow-up methods such as hospitalization, outpatient visits, home visits, and communication according to the severity of the subject's adverse event, and perform unscheduled tests for safety supervision when necessary.

9.6 Overdose

Drug overdose is defined as: a subject using (intentionally or accidentally) a dose exceeding the dose specified in the protocol. If a drug overdose occurs, the subject may be given appropriate supportive treatment after discussion by the investigator and the medical monitor. Any drug overdose event should be reported as a protocol deviation and the medical monitor should be informed. AEs caused by drug overdose should be included in the standard AE record. SAEs caused by drug overdose should be reported according to the standard SAE reporting method and time limit.

9.7 Pregnancy

Throughout the study and for 90 days after the last dose of IMP, subjects should use abstinence or any other effective method of contraception to avoid pregnancy. Women of childbearing potential should be informed of the potential risks, and women of childbearing potential will not be able to breast feeding during the study and for 1 month after the end of treatment. Male subjects of childbearing potential should inform their partners that they are participating in this study and should use effective contraception during treatment and for 90 days after the last dose. Subjects who become pregnant during the study will be withdrawn from the study.

In this study, acceptable methods of effective contraception include:

- Female sterilisation (i.e., documented bilateral tubal ligation)
- Placement of intrauterine device or intrauterine system
- Barrier contraception (condom)
- Male sterilisation (with relevant archived documents proving that the female subject's male partner (the only partner) has undergone vasectomy surgery and has aspermia in the ejaculate)

- Abstinence: Abstinence is the subject's preferred lifestyle, not limited to the contraceptive rhythm method (e.g., calendar, ovulation, symptothermal body temperature, and post-ovulation abstinence methods)

9.7.1 Pregnancy Test

Female subjects of childbearing potential will undergo serum pregnancy test during the screening period and at other time points specified in the protocol. Pregnant female subjects are not suitable for participation in the study. If a female subject has a positive pregnancy test at any time during the study, she should immediately stop taking the IMP.

9.7.2 Time of Pregnancy Information Collection

The time period for collecting pregnancy information is from the screening period to 90 days after the last dose.

9.7.3 Management of Occurring Pregnancy

The investigator or their designee is responsible for collecting pregnancy information from female subjects or female partners of male subjects who become pregnant while participating in this study. If a subject has a positive pregnancy test at any time after receiving the IMP, the subject should discontinue treatment immediately. At the time of discontinuation of study treatment, all relevant test and examination results will be collected as a baseline evaluation.

Subjects must be instructed that if a female subject or a male subject's sexual partner becomes pregnant during the screening period to 90 days after the last dose, the investigator should be notified immediately. The investigator should complete the pregnancy event form and report the pregnancy event to the sponsor within 24 hours of becoming aware of the event. The investigator should make every effort to follow up until 6 to 8 weeks after the end of the pregnancy and report to the sponsor any cases of premature termination of pregnancy or information on the condition of the mother and child. Pregnancy events are not reported as SAEs, but if the outcome of the pregnancy meets the criteria for direct classification of SAEs, such as postpartum complications, spontaneous abortion, abortion induced, stillbirth, death neonatal, congenital deformity (including congenital deformity in aborted fetuses), the investigator should follow the SAE reporting procedure.

10. Statistical Methods

10.1 General Principles

Efficacy and safety evaluation endpoints are detailed in Section 3, Evaluation Endpoints. Statistical analyses of the evaluation endpoints will be performed using SAS 9.4 or a later version for summarization and listing. Specific analysis details will be documented in the Statistical Analysis Plan.

Unless otherwise specified, study data will be statistically described in the following manner:

Continuous variables: number of subjects, mean, standard deviation, median, quartile, minimum and maximum. For pharmacokinetic parameters such as C_{max} and AUC, the geometric mean and geometric coefficient of variation need to be calculated separately;

Categorical variables: frequency and percentage;

Time-to-event variables: number of events, median, 25th and 75th percentiles. Kaplan-Meier survival analysis may be performed, if applicable.

10.2 Sample Size Determination

The progression-free survival (PFS) is used as the primary efficacy endpoint in this study.

A parallel-controlled superiority study design is adopted, with the primary endpoint being investigator-assessed PFS. Subjects are randomized into the test group and control group in a 1:1 ratio. A median PFS of 11 months in the control group and 20 months in the test group is assumed, with a hazard ratio of 0.55. With a one-sided significance level of 0.025 (α=0.025) and a power of 95%, 147 events need to be collected. Assuming an enrollment duration of approximately 18 months and a follow-up of 20 months after the last subject is randomized, an interim analysis will be performed when 70% of the events have been collected. The alpha spending for the interim analysis will be calculated using the Lan-DeMets (O'Brien-Fleming) method, requiring 201 subjects. Considering a 15% dropout rate, this study requires enrollment of at least 238 subjects (119 in the test group and 119 in the control group). The number of events required for the interim analysis is 103.

This study is conducted during the COVID-19 pandemic. Considering that the increased SARS-CoV-2 infection rate among lung cancer patients may lead to higher dropout rates, and the extended study duration (approximately 38 months) can also contribute to increased dropout rates, approximately 268 subjects (134 in the test group and 134 in the control group) with a 25% dropout rate will be enrolled to ensure the study proceeds as planned. The total number of required PFS events remains 147.

10.3 Analysis Population

10.3.1 Full Analysis Set (FAS)

Defined as all successfully screened and randomized subjects.

10.3.2 Safety Set (SS)

Includes all randomized subjects who have received at least one dose of the investigational medicinal product. In the SS, subjects are analyzed according to the actual treatment received.

10.3.3 Per-Protocol Set (PPS)

A subset of subjects in the FAS, excluding those with major protocol deviations affecting outcomes. The list of subjects included in or excluded from PPS must be reviewed and confirmed by the sponsor and investigators before database lock.

10.4 Statistical Analysis

10.4.1 Primary Endpoint Analysis

The primary endpoint of this study is investigator-assessed PFS. Survival rates will be estimated and survival curves will be plotted using Kaplan-Meier method, with 95% CI calculated. The survival functions of the two groups will be compared using the Log-Rank test, taking into account stratification factors. The impact of stratification factors on event endpoints will be considered, with between-group effects and their 95% CIs estimated. The hazard ratio for PFS between groups will be analyzed using COX proportional hazards model in the full analysis set, with brain metastasis and chemotherapy as influencing factors.

Primary endpoint analysis will be based on FAS and PPS, with FAS as the primary analysis set.

10.4.2 Secondary Endpoint Analysis

Analysis methods for secondary endpoints (BICR-assessed PFS, investigator/BICR-assessed OS, DoR) are the same as those for investigator-assessed PFS. OSR at different timepoints will be estimated using Kaplan-Meier method, while the ORR, DCR, CBR, and their 95% CIs will be calculated using the Cochran-Mantel-Haenszel (CMH) test, considering the effects of stratification factors across the groups.

Continuous data will undergo descriptive statistical summarization including: mean, standard deviation, median, minimum, and maximum.

10.4.3 Subject Population Analysis

Descriptive statistics will be performed and summarized for the number of subjects enrolled, the number of subjects in the exclusion analysis set, and the number of subjects who completed or dropped out, with specific reasons for dropout and exclusion.

Descriptive summaries of subjects' demographic information and other baseline characteristics will be provided.

10.4.4 Efficacy Analysis

The tumor assessment results (disease progression, stable disease, complete response, and partial response) will be summarized and listed, and the number and percentage of subjects in each category will be calculated.

ORR is defined as the percentage of subjects in the analysis set whose best overall response is CR or PR according to RECIST 1.1. DCR is defined as the percentage of subjects in the analysis set whose best overall response is CR, PR, or SD according to RECIST 1.1. The ORR and DCR and their exact 95% CIs will be calculated.

- Duration of Response (DoR)

  Limited to subjects with CR or PR, defined as the number of days from the date of first response to the date of PD or death (whichever occurs earlier) (according to RECIST 1.1 criteria). For subjects who have a response but do not experience disease progression and death, the DoR can be defined as the date from the day of first response to the date of the last tumor assessment or the last assessment of disease progression.

  DoR will be described using the Kaplan-Meier method, and its 95% CI will be calculated.

- Progression-Free Survival (PFS)

  PFS is defined as the time from the first study treatment to disease progression or death (whichever occurs earlier). If the subject has not experienced death or disease progression at the end of the study, or if the subject is lost to follow-up, PFS statistics will be censored at the time of the last tumor evaluation.

  The Kaplan-Meier method will be used to describe the median time of PFS and its 95% CI.

- Overall Survival (OS)

OS is defined as the time from the date a subject receives the first study treatment to the date of death due to any cause. If the subject has not experienced death at the end of the study or if the subject is lost to follow-up, the OS statistics will be censored at the date of the last survival follow-up.

The Kaplan-Meier method will be used to describe the median time of OS and its 95% CI, and the 3-month, 6-month, 9-month, and 12-month survival rates will be calculated.

- IC-ORR (Intracranial-Objective Response Rate)

  IC-ORR is defined as the percentage of subjects with intracranial measurable lesions at baseline whose best overall intracranial response is CR or PR according to RANO-BM criteria. The IC-ORR and its exact 95% CI will be calculated.

10.5 Safety Data Analysis

Statistical analysis will be performed on all AEs (NCI CTCAE v5.0), SAEs, drug-related AEs, and drug-related SAEs to evaluate the safety of the investigational medicinal product; all AEs will be classified according to the Medical Dictionary for Regulatory Activities (MedDRA) coding, and all AEs will be summarized and analyzed by organ system and preferred term; the changes from baseline in laboratory tests, vital signs, physical examinations, and 12-lead ECGs in each dose group will be analyzed, and a list of abnormal values will be provided.

10.5.1.1 Adverse Event

AEs will be coded according to ICH (MedDRA). All AEs, including SAEs, will be summarized by system organ class and preferred term and according to relationship to the IMP and severity. The AEs and SAEs leading to death and the AEs leading to dropout will be summarized, and the details of each AE for each subject will be listed, including the type of AE, severity, occurrence and end time, outcome, and relationship to the IMP.

10.5.1.2 Clinical Laboratory Test

The results of laboratory tests, including hematology, urinalysis, coagulation function, and blood chemistry, will be summarized by dose group at the scheduled evaluation time points, and the laboratory test results will be described using before-and-after cross-tabulations. All laboratory test abnormal results will be listed in detail.

10.5.1.3 Other Clinical Safety Endpoints

The results of 12-lead ECG and echocardiography for subjects will be summarized, and the changes in ECGs before and after medication (especially evaluation of the prolongation of PR interval, QRS duration, and QTcF interval) and echocardiogram LVEF will be listed. All ECG abnormal results will be listed in detail.

Vital signs of subjects at planned evaluation time points will be summarized, changes in vital signs before and after medication will be described, and all abnormal results will be listed in detail.

Physical examination data at planned evaluation time points will be summarized, and the examination results will be described using before-and-after cross-tabulation. Abnormal results will be summarized by subject listing.

10.6 Exploratory Analysis

Time to deterioration (TTD) is defined as the time from randomization to first deterioration in EORTC QLQ-C30 scale score. The analysis method will be the same as that for investigator-assessed PFS, using the Kaplan-Meier method to estimate survival rates and plot survival curves, with 95% CIs estimated.

EORTC QLQ-C30 and QLQ-LC13 quality-of-life scores will be aggregated by visit and analyzed for changes from baseline. Domain scores will be calculated based on individual items, and the score results for each domain and the changes from baseline will be summarized and analyzed. A mixed-effects model for repeated measures will be used to compare the changes in quality of life scores from baseline between the two groups, with the model using change relative to baseline as the dependent variable, and group and baseline scores as covariates.

The EQ-5D 5L Health Index score will be computed using a weighted approach, with results summarized by visit and changes from baseline analyzed at each visit point. Domain scores will be calculated based on individual items, and the score results for different domains and the changes from baseline will be summarized and analyzed. A mixed-effects model for repeated measures will be used to compare the changes in quality of life scores from baseline between the two groups, with the model using change relative to baseline as the dependent variable, and group and baseline scores as covariates. Aggregate analysis will summarize frequency by category: 'Mobility', 'Self-care', 'Usual activities', 'Pain/discomfort', and 'Anxiety/depression'.

10.7 Subgroup Analysis

In order to determine the consistency of efficacy across subgroups in this study, the primary endpoint PFS assessed by the investigator based on RECIST v1.1 criteria will be analyzed by subgroup according to the following factors (at least but not limited to).

Stratification factors:

1. Brain metastasis (Yes, No);

2. Chemotherapy (Yes, No).

Subgroup analysis of other potential clinically significant factors:

- Gender

- Age
- ECOG performance status
- Smoking history
- Disease staging
- Prior anti-tumor therapy

Subgroups of other potential clinically significant factors, including but not limited to the above, will be defined in detail and analyzed in the Statistical Analysis Plan (SAP).

11. Quality Assurance and Quality Control

According to GCP guidelines, the sponsor is responsible for using and maintaining quality assurance and quality control systems in accordance with the relevant Standard Operating Procedures (SOPs).

The sponsor or the sponsor's representative should conduct quality control at each stage of data processing to ensure the accuracy, consistency, completeness, and reliability of the data.

In addition, the sponsor or its representative, and the relevant regulatory authorities may conduct audits and/or inspections of the study process. During audits and/or regulatory agency inspections, authorized representatives of the sponsor and relevant regulatory authorities have the right to access all study-related documents.

11.1 Clinical Monitoring

The sponsor or its representative will conduct clinical monitoring of this study. The clinical research associate (CRA) should conduct monitoring according to the corresponding SOP. The CRA should maintain regular communication with the investigator and the sponsor. The CRA will assess the capabilities of each study site and report any issues with facilities, technical equipment, or investigators to the sponsor.

During the study, the CRA will be responsible for monitoring whether written, signed, and dated ICFs have been obtained from all subjects, and whether data recording is accurate and complete. At the same time, the CRA will also compare the data entered in the eCRF with the original data and inform the investigator of any errors or omissions. The CRA will also ensure the study site's compliance with the study protocol, arrange for the supply of IMPs, and ensure that the investigational medicinal products are stored under appropriate conditions as required by the instructions.

The CRA will conduct monitoring visits in accordance with the requirements of all relevant laws and regulations. Each study site will receive regular monitoring visits, starting from the time a subject signs informed consent. The CRA should submit a written report to the sponsor after each visit to the investigator.

11.2 Quality Assurance Audit

During the study, the sponsor or the sponsor's representative will conduct quality assurance audits of the study site, research database, and related research documents. At the same time, the relevant regulatory authorities may also decide to inspect the study site, research database, and related research documents.

12. Data Management

The data management department will process the data generated in this clinical study according to the relevant SOPs.

This study will utilize an Electronic Data Capture (EDC) system, and study data will be entered into the eCRF by the investigator or authorized study site staff. Before the study site is initiated or data is entered, appropriate training will be provided to the investigators and authorized study site staff, and appropriate safety measures will be taken.

All data will be entered in simplified Chinese. The eCRF should be completed as soon as possible during or after the visit and updated at any time to ensure that it reflects the latest status of the subjects participating in the study. To avoid discrepancies in results assessment among different assessors, the baseline and all subsequent efficacy and safety evaluations for the same subject should be performed by the same person whenever possible. The investigator must review the data to ensure the accuracy of all data entered into the eCRF. If certain assessments are not performed during the study, or if certain information is unavailable, not applicable, or unknown, the investigator should record it in the eCRF. The investigator should electronically sign the verified data.

The CRA will review the eCRF and assess its completeness and consistency. The CRA will compare the eCRF with source documents to ensure the consistency of key data. All data entry, corrections, and modifications will be the responsibility of the investigator or their designee, and the CRA will not have this authority. Data in the eCRF will be submitted to the data server, and any changes to the data will be recorded in the audit trail, i.e., the reason for the change, the operator's name, and the time and date of the modification will be recorded. The roles and permissions of the study site staff responsible for data entry will be predetermined. If there are data queries, the CRA or data manager will issue a query in the EDC, and the study site staff will be responsible for answering the query. The EDC system will record the audit trail of queries, including the operator's account name, time, and date.

When study data is entered into the eCRF, the system will automatically add the identity of the data entry person through the ID of the logged-in user. The investigator documents verification of the record and ensures the accuracy of the data in the record via electronic signature. Electronic signatures will be completed using the investigator's user ID and password, and the system will automatically attach the date and time of the electronic signature. If data in the eCRF needs to be changed, it should be done according to the workflow defined by the EDC system. All changes and the reasons for the changes will be recorded in the audit trail.

13. Ethical Considerations

13.1 Ethics Committee (EC)

Prior to the start of the study, the study protocol and other relevant documents (informed consent form, investigator's brochure, case report form, and other documents that may need to be submitted) should be submitted to the appropriate EC for approval. The signed/approved EC opinion will be filed in the investigator's file.

The study must not begin in any form until the sponsor has obtained the corresponding written EC approval/authorization. The EC will be required to provide a document including the meeting date, attendees, and voting members for the signed approval/authorization comment. The approval document should clearly state the study title, protocol version, and informed consent form version. Meeting minutes should also be included where available.

Substantial amendments to the protocol must also be submitted to the relevant EC for approval before implementation. Relevant safety information should also be submitted to the EC during the study in accordance with national regulations and requirements.

13.2 Ethical Conduct in the Study

The conduct of the study and the informed consent process should adhere to the ethical principles outlined in the Declaration of Helsinki, relevant GCP requirements, and the drug and data protection laws and regulations of the country where the study is being conducted.

This study will be conducted in accordance with GCP and relevant national regulations, and will adhere to the relevant ethical principles in the Declaration of Helsinki and international ethical guidelines to protect the rights, safety, and health of subjects.

13.3 Subject Information and Informed Consent

The Informed Consent Form (ICF) is used to explain the nature, significance, benefits and potential risks, inclusion and exclusion criteria, and other information of this study to potential participants before the study begins. The language of the informed consent should be simple and easy to understand. The ICF statement should clearly state that informed consent is signed voluntarily, clarify the potential risks and benefits of participating in this study, and state that subjects can withdraw from the study at any time. The subject and/or their legal representative can only sign the written consent after having a thorough understanding of the study-related matters.

The investigator is responsible for ensuring that an ICF, signed and dated by the subject or their legal representative, has been obtained prior to the start of the study and before the start of drug therapy. This indicates that the subject is fully informed and voluntarily agrees to participate in

this study. The authorized investigator who performs the informed consent process also needs to sign and date the informed consent form in the corresponding position.

After signing, the investigator shall provide each subject with a copy of the ICF with the names and dates of both parties.

13.4 Subject Data Protection

The ICF will include (or, in some cases, be accompanied by a separate document) information regarding data protection and privacy.

The sponsor will assign a unique identification number to the subject. Any subject records or datasets transferred to the sponsor may only contain this identification number; the subject's name or any information that could identify the subject must not be transferred.

14. Study Management

14.1 Management Structure

If contact information changes during the study, the sponsor will notify the investigator in writing, without making supplements to the protocol.

14.2 Overall Study Premature Termination and Study Site Closure

The sponsor has the right to discontinue this study at any time. The sponsor has the right to close a study site at any time.

14.3 Study Document Retention

Clinical study documents (protocol and protocol amendments, completed eCRFs, signed ICFs, laboratory test results, and drug inventory records, etc.) should be retained and managed in accordance with GCP requirements or local regulations.

For clinical studies intended for drug registration application, the essential documents should be retained at least 5 years after the investigational medicinal product is approved for marketing; for clinical studies not intended for drug registration application, the essential documents should be retained at least 5 years after the clinical study is terminated. If a longer retention period is required by relevant regulations or agreements with the sponsor, the longer period should be followed. If local regulations require a shorter retention period, it may be implemented according to the actual situation, provided that it does not affect the clinical trial application and the verification/inspection by the drug regulatory authorities.

No study documents may be destroyed without the written permission of the sponsor and the investigator. Study documents may only be transferred to another party that complies with document retention requirements or moved to another location that meets the requirements after notifying the sponsor and receiving its written consent.

14.4 Access to Original Data/Documents

Original documents are defined as the original observations and clinical study records. Original documents may include, but are not limited to, medical history, study progress notes, email communications, computer data output, laboratory data, and data recorded in automated instruments. All original documents obtained in this study will be kept by the investigator for monitoring and auditing by the sponsor's representatives and/or for on-site inspection by regulatory authorities.

Depending on the specifics of the study, the investigator may need to obtain previous medical records or referral records. In addition, a record of the present medical history must also be provided.

The investigator should prepare original documents for each subject who is randomly enrolled in the study. The content should be comprehensive and accurate to record all test results and other relevant data, and these documents should be properly preserved.

By participating in this study, the investigator agrees that the results of this study may be used for submissions to national and/or international registration and regulatory authorities. If these competent authorities make a request, the investigator's name, address, qualifications, and extent of participation in the study will need to be provided to these authorities. The investigator needs to provide all study data and complete reports to the sponsor so that these authorities can review all study records.

The data from this study must be available for on-site inspection by any regulatory agency, the sponsor, and the IRB/IEC, as appropriate. Subject medical information obtained during this study is confidential and may not be disclosed to third parties other than those listed above.

14.5 Investigator Responsibilities

This study should be conducted in compliance with the ethical principles of the Declaration of Helsinki, relevant GCP requirements, and the drug and data protection laws and regulations of China.

The investigator should follow the study protocol and record the data in the eCRF according to the protocol requirements. The data records should be true, accurate, complete, timely, legal, and clear.

Detailed responsibilities of relevant investigators are listed in the current GCP of China.

14.6 Amendment to Study Protocol

Any potentially appropriate amendments to the protocol during the course of the study will be communicated and agreed upon by the sponsor and the Investigator. The sponsor should ensure timely submission of protocol amendments to the regulatory authorities.

All protocol amendments (except for changes to the protocol to eliminate immediate hazards to subjects participating in the study) must be implemented only after approval by the EC and regulatory authorities (if required). Changes to the protocol to eliminate an immediate hazard to subjects may be implemented prior to EC approval.

14.7 Protocol Deviation

The investigator should conduct the study in accordance with the protocol agreed upon by the sponsor, and approved by the Regulatory Authority (if required) and the Ethics Committee.

Deviations from the study protocol are not permitted unless they are necessary to eliminate immediate hazard to a subject participating in the study. Each investigator will be responsible for enrolling only subjects who meet the inclusion criteria and do not meet the exclusion criteria

of the protocol. If the study site fails to conduct the study according to the protocol, the sponsor or the sponsor's representative reserves the right to close the study site.

If a study protocol deviation occurs, the investigator must notify the sponsor or the sponsor's designee, and any protocol deviation must be recorded in the protocol deviation report form and classified according to the type of deviation. Protocol deviations include, but are not limited to, the following:

- Enrolling subjects who do not meet the study's inclusion criteria

- Failure to withdraw subjects from the study who meet the withdrawal criteria during the study

- Use of incorrect dosage

- Receiving protocol-prohibited concomitant therapies

14.8 Publication Policy

All data generated in this study are confidential information of the sponsor. The sponsor reserves the right to publish the study results. Information regarding sponsor and investigator publication policies will be described in the Clinical Study Agreement.

14.9 Financial and Insurance Information

The sponsor will manage the financial expenditures for this study in accordance with local regulations and requirements, and the sponsor will purchase insurance. Insurance-related clauses will be kept in the study folder.

Financial and insurance information for this study is listed in the Clinical Study Agreement, and the contents must comply with all local and national regulations.

If it is proven that a subject suffers study-related injury or death, the sponsor will agree to comply with the current NMPA requirements and provide appropriate compensation to the subject.

15. References

[1] Dela Cruz CS, Tanoue LT, Matthay RA. Lung cancer: epidemiology, etiology and prevention [J].Clin Chest Med, 2011, 32 (4):605-644.

[2] Pikor LA, Ramnarine VR, Lam S, et al. Genetic alterations defining NSCLC subtypes and their therapeutic implications[J]. Lung Cancer, 2013, 82 (2): 179-189.

[3] Zhang D. Research progress on driver gene mutations and targeted therapy in non-small cell lung cancer. Chinese Journal of Lung Cancer [J],2014,17(10):750-754.

[4] Zhang XC, Zhang S, Yang XN, et al. Fusion of EML4 and ALK is associated with development of lung adenocarcinomas lacking EGFR and KRAS mutations and is correlated with ALK expression[J]. Mol Cancer, 2010, 9: 188.

[5] Hong S, Fang W, Hu Z, et al. A large-scale cross-sectional study of ALK rearrangements and EGFR mutations in non-small-cell lung cancer in Chinese Han population[J]. Sci Rep, 2014, 4: 726-728.

[6] D. Ross Camidge, William Pao, Lecia V. Sequist. Acquired resistance to TKIs in solid tumours: learning from lung cancer[J]. Nat. Rev. Clin. Oncol. 11, 473-481 (2014).

[7] Jiang T, Zhou CC. Mechanisms and treatment strategies for crizotinib resistance in patients with ALK-positive non-small cell lung cancer [J]. Chinese Journal of Lung Cancer, 2015,18(2): 69-74.

[8] State Council of the People’s Republic of China Decree No. 360. Regulations for the Implementation of the Drug Administration Law of the People’s Republic of China. 2019.03.

[9] National Medical Products Administration. Good Clinical Practice. 2020.04.

[10] Technical Guidelines for Clinical Studies of Anti-tumor Drugs, CFDA, 2012.

[11] Nonclinical Evaluation for Anticancer Pharmaceuticals, ICH, 2010.

[12] Guidelines for the Prevention and Treatment of Vomiting Related to Cancer Therapy (2014 Edition).

[13] Lin TY, Yu SY, and Jiao SC. Targeted Therapy for Neoplasm Malignant, People's Medical Publishing House, 2016.11.

16. Appendices

16.1 Appendix 1: Laboratory Safety Test

Clinical laboratory tests include hepatitis B five items, HCV, HIV, hematology, blood chemistry, coagulation function, glycosylated haemoglobin (HbA1c), blood human chorionic gonadotropin (HCG) and urinalysis, vital signs. See Table 16-1, Table 16-2, Table 16-3, Table 16-4, Table 16-5, Table 16-6, Table 16-7, Table 16-8 for details. If abnormal laboratory test results occur in the study, they should only be recorded as AEs if they are determined to be clinically significant by the investigator. Repeated laboratory tests may be performed, if needed, to confirm the accuracy of laboratory test results.

Table 16-1 Hepatitis B Five Items, HCV, HIV

**Hepatitis B Five Items, HCV, HIV (*Vacuum tube*)**	
Hepatitis B virus surface antigen (HBsAg)	Hepatitis B virus core antibody (Anti-HBc)
Hepatitis B virus surface antibody (Anti-HBs)	Hepatitis C virus specific antibody (Anti-HCV)
Hepatitis B virus e antigen (HBeAg)	Human immunodeficiency virus antibody (anti-HIV)
Hepatitis B virus e antibody (anti-HBe)	

Table 16-2 Hepatitis B Virus Deoxyribonucleic Acid

**Hepatitis B Virus Deoxyribonucleic Acid *(Serum separation tube)***
Hepatitis B Virus Deoxyribonucleic Acid (HBV-DNA)

Note: If HBsAg is positive and/or HBcAb is positive, further HBV-DNA testing is required to confirm presence of chronic active hepatitis B.

Table 16-3 Hematology Parameters

**Hematology *(EDTA-K2 tube)***	
White blood cell count (WBC)	Neutrophil count (NEUT)
Red blood cell count (RBC)	Neutrophil percentage (NEUTP)
Haemoglobin (HGB)	Lymphocyte count (LYMPHN)
Haematocrit (HCT)	Lymphocyte percentage (LYMPHP)
Platelet count (PLT)	Monocyte count (MONON)
Eosinophil count (EON)	Monocyte percentage (MONOP)
Eosinophil percentage (EOP)	Basophil percentage (BASOP)
Basophil count (BASON)	

Table 16-4 Blood Chemistry Parameters

**Blood Chemistry** ***(Serum separation tube)***	
Potassium (K)	Total protein (TP)
Sodium (Na)	Albumin (ALB)
Chloride (Cl)	Total bilirubin (TBIL)
Magnesium (Mg)	Direct bilirubin (DBIL)
Calcium (Ca)	Alanine aminotransferase (ALT)
Creatinine (CREA)	Aspartate aminotransferase (AST)
Urea (UREA)/Blood urea nitrogen (BUN)	Alkaline phosphatase (ALP)
Total cholesterol (TCHO)	Lactate dehydrogenase (LDH)
Triglycerides (TG)	Blood glucose (GLU)
Low density lipoprotein (LDL-C)	Serum lipase (LPS)
High density lipoprotein (HDL-C)	Serum amylase (AMS)
Creatine kinase (CK)	Creatine kinase isoenzyme (CK-MB)

Table 16-5 Coagulation Parameters

**Coagulation** ***(Sodium citrate tube)***	
Activated partial thromboplastin time (APTT)	International normalized ratio (INR)
Prothrombin time (PT)	Fibrinogen (FIB)

Table 16-6 Glycosylated Haemoglobin

**Glycosylated Haemoglobin (HbA1c)** ***(Serum separation tube)***
Glycosylated haemoglobin (HbA1c)

Table 16-7 Blood Human Chorionic Gonadotropin

**Human Chorionic Gonadotropin (HCG)** ***(Serum separation tube)***
Human chorionic gonadotropin (HCG)

Table 16-8 Urinalysis Parameters

**Urinalysis (Fresh Urine Sample)**	
White blood cells urine (WBC)	Red blood cells urine (RBC)
Protein urine (PRO)	Ketone bodies (KET)
Urine glucose (GLU)	pH value

16.2 Appendix 2: ECOG Performance Status

Table 16-9 ECOG Performance Status Scale

Activity Score	Description
0	Full active, able to carry on all pre-disease performance without restriction.
1	Restricted in physically strenuous activity but ambulatory and able to carry out work of a light or sedentary nature, e.g., light house work, office work.
2	Ambulatory and capable of all self-care but unable to carry out any work activities; up and about more than 50% of waking hours.
3	Capable of only limited self-care; confined to bed or chair more than 50% of waking hours.
4	Completely disabled; cannot carry on any self-care.
5	Death.

16.3 Appendix 3: Fridericia's Formula

QTcF is calculated using the Fridericia formula: QTcF = QT/(RR^0.33).

16.4 Appendix 4: Response Evaluation Criteria in Solid Tumors 1.1 (RECIST v1.1)

16.4.1 Inclusion Criteria

Only subjects with measurable tumor at baseline may be enrolled in study protocols using tumor objective response as the primary endpoint.

16.4.2 Definitions

Measurable lesions: At least one measurable lesion must be present. Lesions that can be accurately measured using at least one dimension, with a longest diameter ≥10 mm on 5 mm thin-slice CT, short axis of lymph nodes ≥15 mm; longest diameter ≥20 mm on well-contrasted chest X-ray; skin nodules, etc. on the body surface that can be measured with calipers, if ≥10 mm, can also be used as measurable lesions, and their size should be clearly marked with scaled color photos. If imaging technology can evaluate, imaging technology evaluation should be preferred.

Non-measurable lesions: All other lesions, including small lesions (longest diameter <10 mm or pathological lymph nodes with 10 to <15 mm short-axis) as well as truly non-measurable lesions. Lesions considered truly non-measurable include: leptomeningeal disease, ascites, pleural or pericardial effusion, inflammatory breast cancer, lymphangitic involvement of skin or lung, and abdominal masses or abdominal organomegaly that is not measurable by reproducible by imaging techniques.

Special considerations regarding lesion measurability: Include bone lesions, cystic lesions, and lesions with prior local treatment. Bone scan, PET scan, and X-rays cannot be used to measure bone lesions. However, they can be used to confirm their presence and resolution. Lytic bone lesions or mixed lytic-blastic lesions, with identifiable soft tissue lesions that can be evaluated by tomographic techniques (e.g., CT or MRI) can be considered as measurable target lesions if the soft tissue lesions meet the criteria for measurability. When both non-cystic and cystic lesions are present, non-cystic lesions should be prioritized as target lesions. Lesions situated in a previously irradiated area, or in an area subjected to loco-regional therapy, are usually not considered measurable unless progression has occurred.

All measurements should be recorded in metric notation, using rulers or calipers. All baseline evaluations should be performed as close as possible to the treatment start and never more than 4 weeks before the beginning of the treatment.

16.4.3 Measurement Methods

The same method of assessment and the same technique should be used to characterise each identified and reported lesion at baseline and during follow-up.

- When clinically detected lesions are superficial (e.g., skin nodules, palpable lymph nodes), they are considered measurable lesions. For the case of skin lesions, documentation by colour photography including a ruler to estimate the size of the lesion is suggested.
- Using CT and MRI to measure target lesions is currently the best, most accessible, and reproducible method. Routine CT and MRI scans are performed using a consecutive 10 mm slice thickness. Spiral CT should be performed using a 5 mm contiguous reconstruction

algorithm. This standard is suitable for thoracic, abdominal, and pelvic tumors, while head and neck tumors and limb tumors usually require special regimes.

- Lesions assessed by X-ray examination can be considered measurable when they are clearly defined and surrounded by aerated lung. However, CT scans are preferred.
- When the primary endpoint of the study is objective response assessment, ultrasound (US) should not be used to measure tumor lesions, as this method is not easily accepted clinically. This method can be used as an alternative to clinical measurement of palpable superficial lymph nodes, subcutaneous lesions, and thyroid nodules. US methods can also be used to confirm the complete disappearance of superficial lesions usually assessed by clinical test.
- Tumor markers alone cannot be used to assess tumor response. If the markers are initially above the upper normal limit, however, they must normalise for a patient to be considered in clinical complete response when all tumor lesions have disappeared. Elevation of tumor markers must be accompanied by visible disease progression to meet the requirements for disease progression.
- In rare situations, cytology and histology may be used to differentiate between PR and CR (e.g., to distinguish residual benign lesions, such as germinoma, from residual malignant lesions after treatment).
- The cytological confirmation of the neoplastic origin of any effusion that appears or worsens during treatment must be performed if the measurable tumor has met the criteria for response or stable disease in order to differentiate between response, stable disease, or progressive disease.

16.4.4 Tumor Response Evaluation

16.4.4.1 Baseline Record of "Target" and "Non-Target" Lesions

- All measurable lesions, up to 2 lesions per organ, with a maximum total of 5 lesions, and representative of all involved organs, should be identified as target lesions and recorded and measured at baseline.
- Target lesions should be selected based on their size (lesions with the largest diameter) and suitability for accurate and repeatable measurement (either by imaging techniques or clinically).
- The sum of the longest diameters (LD) for all target lesions will be calculated and reported as the baseline sum of LDs. The sum of baseline LD will be used as the reference for describing objective tumors.
- All other lesions (or sites of disease) should be identified as non-target lesions and should also be reported at baseline. Measurement is not required for these lesions, but the presence

or absence of each lesion should be recorded throughout the follow-up period.

16.4.4.2 Response Criteria

Target Lesion Evaluation Criteria	
Complete Response (CR)	Disappearance of all target lesion. Any pathological lymph nodes (whether target or non-target) must have reduction in short diameter to <10 mm.
Partial Response (PR)	At least a 30% decrease in the sum of the LDs of target lesions, taking as reference the baseline sum LDs.
Stable Disease (SD)	Neither sufficient shrinkage to qualify for PR nor sufficient increase to qualify for PD, taking as reference the smallest recorded LD sum since treatment initiation.
Progressive Disease (PD)	At least a 20% increase in the sum of LD of target lesions, taking as reference the smallest sum since treatment initiation (this includes the baseline sum if that is the smallest). In addition to the relative increase of 20%, the sum must also demonstrate an absolute increase of at least 5 mm. (Note: the appearance of one or more new lesions is also considered progression)
**Non-target Lesion Evaluation Criteria**	
Complete Response (CR)	Disappearance of all non-target lesions and normalisation of tumor marker level. All lymph nodes must be non-pathological (i.e., short diameter <10 mm).
Non-CR/Non-PD	Persistence of one or more non-target lesion and/or maintenance tumor marker level above normal limit.
Progressive Disease (PD)	Appearance of ≥1 new lesion and/or unequivocal progression of existing non-target lesions.

*Although unequivocal progression in "non-target" lesions is an exception, this should be based on the treating physician's opinion, with the progression confirmed subsequently.

16.4.4.3 Evaluation Criteria for Best Overall Response

Best overall response is the best response recorded from the start of treatment until disease progression/recurrence (using the smallest measurement recorded since the start of treatment as the reference value for PD). The best overall response of a subject generally depends on the completion of two measurements and confirmation criteria.

Subjects who experience an overall deterioration in health requiring treatment discontinuation, but without objective evidence of disease progression at that time, should be reported as "symptomatic deterioration". Objective progression should be recorded whenever possible, even after treatment discontinuation.

In some cases, it is difficult to distinguish residual lesions from normal tissue. When the evaluation of complete response depends on such a decision, it is recommended to study (fine needle aspiration/biopsy) the residual lesions to confirm the complete response status.

Target Lesions	Non-Target Lesions	New Lesions	Overall Response
CR	CR	No	CR
CR	Non-CR/Non-PD	No	PR
CR	NE	No	PR
PR	Non-PD or NE	No	PR

SD	Non-PD or NE	No	SD
Not all evaluated	Non-PD	No	NE
PD	Any	Yes or No	PD
Any	PD	Yes or No	PD
Any	Any	Yes	PD

Note: CR=Complete Response; PR=Partial Response; SD=Stable Disease; PD=Progressive Disease; NE=Not Evaluable

For subjects with non-measurable lesions (non-target lesions), the overall response status will also be assessed at the tumor measurement time points, as detailed in the table below.

Overall Response Assessment in Subjects with No Measurable Disease at Baseline

**Non-Target Lesions**	**New Lesions**	**Overall Response**
CR	No	CR
Non-CR/Non-PD	No	Non-CR/Non-PD
Not all evaluated	No	NE
Unequivocal PD	Yes or No	PD
Any	Yes	PD

Abbreviations: CR=Complete Response; PD=Progressive Disease; NE=Not Evaluable

16.5 Appendix 5: RANO-BM

Measurable lesions are defined as contrast-enhanced lesions that can be accurately measured in at least one dimension, with a minimum size of 10 mm, and visible in two or more axial layers with a slice thickness of 3 mm or less and a spacing of 0 mm. In addition, the diameter perpendicular to the long diameter in the plane of measurement should be at least 5 mm.

For previously treated lesions, full information on how each lesion was treated must be documented.

- RANO-BM criteria:

Criteria	CR[b]	PR[b]	SD	PD
Target Lesions - Up to 5 measurable lesions	No	Compared with BL, sum of LD ≥30%↓	Compared to the lowest point, sum of LD <30%↓ but <20%↑	Compared with the lowest point[a], sum of LD ≥20%↑
Non-Target Lesions	No	Stable or improved	Stable or improved	Unequivocal disease progression[a]
New Lesions[c]	No	No	No	Yes[a]
Corticosteroid Hormones	No	Stable or alleviated	Stable or alleviated	Not applicable[d]
Clinical Status	Stable or improved	Stable or improved	Stable or improved	Worsening[a]
Efficacy Requirements	All	All	All	Any[d]

Note: BL, Baseline; CR, Complete Response; PD, Progressive Disease; PR, Partial Response; LD, Longest Diameter; SD, Stable Disease.

a) Disease Progression is indicated when this criterion is met.

b) For CR and PR, response must be maintained for at least 4 weeks.

c) New lesions are lesions that were not present on previous scans and are visible in the smallest two projections. If a new lesion is suspected, such as due to its small size, continued treatment may be considered, with follow-up assessments to determine whether the new lesion is a new disease. If repeat scans confirm the presence of a new lesion, progression should be declared using the original scan date showing the new lesion.

d) Single increases in corticosteroids are not considered when determining progression in the absence of sustained clinical deterioration.

Response summaries for target and non-target lesions are as follows:

Target Lesions

Complete Response

- All CNS target lesions disappear for at least 4 weeks;
- No new lesions;
- No corticosteroids used; and
- Patient clinically stable or improved.

Partial Response

- At least a 30% decrease in the sum of the longest diameter of CNS target lesions, compared to baseline longest diameter, sustained for at least 4 weeks;
- No new lesions;
- Corticosteroid dose stable to decreased; and
- Clinically stable or improved.

Disease Progression

- The sum of the longest diameter of CNS target lesions increases by at least 20% compared to the smallest sum in the study (including the baseline sum if it is the smallest in the study).
- In addition to a relative increase of 20%, the absolute value of at least one lesion must increase by 5 mm or more to be considered progression.

Stable Disease

- Neither sufficient shrinkage to qualify for PR nor sufficient increase to qualify for PD, taking as reference the smallest sum diameters while on study.

Non-Target Lesions

Non-target lesions should be qualitatively assessed at each time point specified in the protocol.

Complete Response

All of the following:

- Disappearance of all enhanced CNS non-target lesions,
- No new CNS lesions.

Non-CR or Non-PD

- Persistence of one or more non-target CNS lesions.

Disease Progression

Any of the following:

- Unequivocal progression of existing enhanced non-target CNS lesions, new tumors (except while on immune-based therapy)
- Unequivocal progression of existing tumor-associated non-enhanced (T2/FLAIR) CNS lesions. In immune-based therapy, new lesions alone may not constitute disease progression.

16.6 Appendix 6: Cockcroft-Gault Formula

Ccr (mL/min) = (Male) (140 - Age) × Weight (kg) / (72 × Blood creatinine (mg/dL))

(Female) (140 - Age) × Weight (kg) / (85 × Blood creatinine (mg/dL))

Ccr (mL/min) = (Male) (140 - Age) × Weight (kg) × 1.23 / Serum creatinine (μmol/L)

(Female) (140 - Age) × Weight (kg) ×1.04 / Serum creatinine (μmol/L)

Note: Ccr is endogenous creatinine clearance rate; age is in years, and weight is in kg.

16.7 Appendix 7: Best Supportive Care

1 Nausea, Vomiting [12,13]

1.1 NCI-CTCAE v5.0 Grading for Nausea and Vomiting

Gastrointestinal Disorders					
	Grading				
Adverse Event	1	2	3	4	5
Nausea	Loss of appetite without alteration in eating habits	Oral intake decreased without significant weight loss, dehydration or malnutrition	Inadequate oral caloric or fluid intake; tube feeding, TPN, or hospitalization indicated	-	-
Definition: A disorder characterized by a queasy sensation and/or the urge to vomit.					
Vomiting	No intervention required	Outpatient IV hydration; medical intervention indicated	Nosogastric gavage, total parenteral nutrition, or hospitalization indicated	Life-threatening	Death
Definition: A disorder characterized by the reflexive act of ejecting the contents of the stomach through the mouth.					

1.2 Definition of Best Supportive Care for Nausea and Vomiting

Recommended regimen:

- 5-HT3 receptor antagonist ± psychotropic drugs (lorazepam preferred) ± H2 receptor antagonist or proton pump inhibitor
- Severe vomiting lasting for several days may lead to electrolyte imbalances, including hypokalemia, hypochloremia, and metastasis hyponatraemia. Fluid replacement and electrolyte correction should be actively given.

Specific Drugs and Administration:

5-HT$_3$ Receptor Antagonists

- Tropisetron: a daily dose of 5 mg, administered intravenously on the first day, followed by oral capsules of 5 mg daily for a maximum of 5 days. It should be noted that doses above 10 mg should be avoided in subjects with uncontrolled hypertension of whom the blood pressure changes should be closely monitored, and the dose of the drug should be adjusted in a timely manner based on changes in blood pressure.
- Ramosetron: 0.1 mg orally disintegrating tablets for adults, 0.3 mg by intravenous injection, once daily. The dosage can be adjusted appropriately depending on age

and symptoms. If the effect is not obvious, the same dose can be added, but the daily dose should not exceed 0.6 mg.

- Azasetron: usually 10 mg for intravenous injection in adults, once daily.

Psychotropic Drugs

- Lorazepam: 0.5~2 mg orally or intravenously or sublingually every 4～6 hours.
- Olanzapine: 2.5~5 mg orally, twice daily.
- Alprazolam: 0.5~2 mg orally, tid.

For patients with gastric disorders, H_2 receptor antagonists or proton pump inhibitors can be used according to clinical routine practice,

with fluid replacement and correction of electrolyte balance.

- If serum potassium is <3.5 mmol/L and symptoms occur, it is recommended to administer 10-20 mL of 10% potassium chloride in 1.0 L of 5% glucose solution. Each gram of potassium chloride must be slowly and evenly infused over 30-40 min, and intravenous bolus injection is strictly prohibited. At the same time, serum potassium and electrocardiogram should be monitored to avoid hyperkalemia. Also, potassium supplementation should only be considered when the subject's urine output is above 30 mL/h.
- Hyponatraemia is often caused by hypokalaemia, which leads to the transfer of extracellular sodium into cells. The total sodium is normal, but serum sodium is decreased. Therefore, treatment is mainly aimed at correcting hypokalaemia.

2 Diarrhoea

2.1 CTC AE Grading for Diarrhoea

	**Grading**				
**Adverse Event**	**1**	**2**	**3**	**4**	**5**
Diarrhoea	Increase of <4 stools per day over baseline; mild increase in ostomy output compared to baseline	Increase of 4 - 6 stools per day over baseline; moderate increase in ostomy output compared to baseline	Increase of ≥7 stools per day over baseline; hospitalization indicated; severe increase in ostomy output compared to baseline; limiting self care ADL	Life-threatening consequences; urgent intervention indicated	Death
Definition: A disorder characterized by frequent and watery bowel movements.					

2.2 Definition of Best Supportive Care for Diarrhoea

Recommended regimen and usage:

- Loperamide: Administer 30 min before meals, the initial dose for persistent diarrhoea is 4 mg, followed by 2-4 h once, or 2 mg after each loose stool, with a maximum daily dose of 16 mg. It should be noted that aggressive dosing may cause ileus paralytic, so when the drug is used for more than 1 day, attention should be paid to adjusting the dosage in a timely manner according to the number of daily bowel movements (dose titrated to 1 to 2 bowel movements per day) (Source: FDA Neratinib lable);
- Mucosal protectants such as montmorillonite powder can also be used, with a recommended dose of 1 bag tid for adults, and the initial dose should be doubled for acute diarrhoea;
- Subjects with frequent diarrhoea should be closely monitored for fluid intake and output balance, and subjects with severe dehydration should be promptly supplemented with water, electrolytes, and nutrients.

16.8 Appendix 8: Quality of Life Questionnaire (EORTC QLQ-C30)

We are interested in some things about you and your health. Please answer all of the questions yourself by circling the number that best applies to you. There are no "right" or "wrong" answers. The information that you provide will remain strictly confidential.

	Not at All	A Little	Quite a Bit	Very Much
1. Do you have any trouble doing strenuous activities, like carrying a heavy shopping bag or a suitcase?	1	2	3	4
2. Do you have any trouble taking a long walk?	1	2	3	4
3. Do you have any trouble taking a short walk outside of the house?	1	2	3	4
4. Do you need to stay in bed or a chair during the day?	1	2	3	4
5. Do you need help with eating, dressing, washing yourself or using the toilet?	1	2	3	4

**During the past week:**	Not at All	A Little	Quite a Bit	Very Much
6. Were you limited in doing either your work or other daily activities?	1	2	3	4
7. Were you limited in pursuing your hobbies or other leisure time activities?	1	2	3	4
8. Were you short of breath?	1	2	3	4
9. Have you had pain?	1	2	3	4
10. Did you need to rest?	1	2	3	4
11. Have you had trouble sleeping?	1	2	3	4
12. Have you felt weak?	1	2	3	4
13. Have you lacked appetite?	1	2	3	4
14. Have you felt nauseated?	1	2	3	4
15. Have you vomited?	1	2	3	4
16. Have you been constipated?	1	2	3	4

**During the past week:**	Not at All	A Little	Quite a Bit	Very Much
17. Have you had diarrhea?	1	2	3	4
18. Were you tired?	1	2	3	4
19. Did pain interfere with your daily activities?	1	2	3	4
20. Have you had difficulty in concentrating on things, like reading a newspaper or watching television?	1	2	3	4
21. Did you feel tense?	1	2	3	4
22. Did you worry?	1	2	3	4

23. Did you feel irritable?	1	2	3	4
24. Did you feel depressed?	1	2	3	4
25. Have you had difficulty remembering things?	1	2	3	4
26. Has your physical condition or medical treatment interfered with your family life?	1	2	3	4
27. Has your physical condition or medical treatment interfered with your social activities?	1	2	3	4
28. Has your physical condition or medical treatment caused your financial difficulties?	1	2	3	4

For the following questions, please circle the number between 1 and 7 that best applies to you.

29. How would you rate your overall health during the past week?

1 2 3 4 5 6 7

Very poor Excellent

30. How would you rate your overall quality of life during the past week?

1 2 3 4 5 6 7

Very poor Excellent

16.9 Appendix 9: Quality of Life Score (EORTC QLQ-LC13)

We are interested in some things about you and your health. Please answer all of the questions yourself by circling the number that best applies to you. There are no "right" or "wrong" answers. The information that you provide will remain strictly confidential.

**During the past week:**	**Not at All**	**A Little**	**Frequent**	**Very Frequent**
1. Did you often cough?	1	2	3	4
2. Did you cough up blood (with blood in sputum)?	1	2	3	4
3. Did you feel short of breath at rest?	1	2	3	4
4. Did you feel short of breath when you walked?	1	2	3	4
5. Did you feel short of breath as you climbed stairs?	1	2	3	4
6. Did you have pain in your mouth or tongue?	1	2	3	4
7. Did you have difficulty in swallowing?	1	2	3	4
8. Did you have any tingling/stinging in your hands and feet?	1	2	3	4
9. Did you ever have hair loss?	1	2	3	4
10. Did you have chest pain?	1	2	3	4
11. Did you have pain in your arm or shoulder?	1	2	3	4
12. Did you have pain in other parts of your body? If yes, where: ___	1	2	3	4
13. Did you take painkillers?<br>(1) No; (2) Yes. If yes, how much did it help?	1	2	3	4

16.10 Appendix 10: Health Index Questionnaire (EQ-5D 5L)

Under each heading, please tick the ONE box that best describes your health TODAY.

Mobility

☐ I have no problems in walking about

☐ I have slight problems in walking about

☐ I have moderate problems in walking about

☐ I have severe problems in walking about

☐ I am unable to walk about

Self-care

☐ I have no problems washing or dressing myself

☐ I have slight problems washing or dressing myself

☐ I have moderate problems washing or dressing myself

☐ I have severe problems washing or dressing myself

☐ I am unable to wash or dress myself

Usual Activities

☐ I have no problems doing my usual activities

☐ I have slight problems doing my usual activities

☐ I have moderate problems doing my usual activities

☐ I have severe problems doing my usual activities

☐ I am unable to do my usual activities

Pain/Discomfort

☐ I have no pain or discomfort

☐ I have slight pain or discomfort

☐ I have moderate pain or discomfort

☐ I have severe pain or discomfort

☐ I have extreme pain or discomfort

Anxiety/Depression

☐ I am not anxious or depressed

☐ I am slightly anxious or depressed

☐ I am moderately anxious or depressed

☐ I am severely anxious or depressed

☐ I am extremely anxious or depressed

16.11 Appendix 11: Investigator Signature Page

Study Title: A Phase 3, Multicenter, Randomized, Open-label Clinical Study Comparing the Efficacy and Safety of XZP-3621 Tablets versus Crizotinib Capsules in Patients with ALK+ Advanced Non-Small Cell Lung Cancer

Protocol Number: XZP-3621-3001

Version Number: V2.0/December 19, 2022

This protocol is a confidential document of Shandong Xuanzhu Pharma Co., Ltd. and shall only be disseminated on a confidential basis. I confirm that I have completely read this protocol, understand its contents, and will carry out my work in accordance with this protocol. In addition, I will conduct my work in accordance with the ethical principles of the Declaration of Helsinki, Good Clinical Practice guidelines, and applicable laws and regulations. By signing this document, I agree that: without the prior written permission of Shandong Xuanzhu Pharma Co., Ltd., it is prohibited to disclose or reveal the non-public information contained herein.

I have read the entire contents of this protocol and agree to follow this protocol in the study.

Investigator's Signature: ____________________________

Date: ____________________________

Printed Name: ____________________________

Investigator Title: ____________________________

Study Site Name/Address: ____________________________
