# Supplementary material for: Dirozalkib versus Crizotinib in Advanced Anaplastic Lymphoma Kinase Inhibitor-Naïve ALK-Positive Non-Small-Cell Lung Cancer: A Multicenter, Randomized Phase III Trial
Source: Cancer Commun (Lond). 2026 Nov 10;46:0068. doi: 10.34133/cancomm.0068 (PMC13748627; doi:10.34133/cancomm.0068)
Supplement: Supplementary file 1 [file cancomm.0068.f1.zip › CANCOMM-D-26-00211-Supplementary Materials.docx]

**Supplementary materials for**

**Dirozalkib versus Crizotinib in Advanced ALK Inhibitor-Naïve *ALK*-Positive Non-Small Cell Lung Cancer: A Multicenter, Randomized Phase III Trial**

Ying Liu^1†^, Xiangjiao Meng^2†^, Yan Yu^3†^, Jianhua Chen^4†^, Runxiang Yang^5^, Laiyu Liu^6^, Yanqiu Zhao^7^, Ruifen Tian^8^, Dianming Li^9^, Ruiling Ning^10^, Qingshan Li^11^, Guowu Wu^12^, Li Wang^13^, Xianghui Duan^13^, Xuejie Guo^13^, Yan Hu^13^, Weili Liu^13^, Haifeng Liu^1*^

^1^Department of Oncology, Jilin Provincial Cancer Hospital, Changchun, Jilin, P. R. China.

^2^Department of Radiation Oncology, Shandong Cancer Hospital and Institute, Shandong First Medical University and Shandong Academy of Medical Sciences, Jinan, Shandong, P. R. China.

^3^Department of Thoracic Medical Oncology, The Affiliated Cancer Hospital of Harbin Medical University, Harbin, Heilongjiang, P. R. China.

^4^Department of Thoracic Medical Oncology, Hunan Provincial Cancer Hospital, Changsha, Hunan, P. R. China.

^5^Department of Medical Oncology, Yunnan Provincial Cancer Hospital, Kunming, Yunnan, P. R. China.

^6^Department of Respiratory and Critical Care Medicine, Nanfang Hospital Affiliated to Southern Medical University, Guangzhou, Guangdong, P. R. China.

^7^Department of Thoracic Medical Oncology, The Affiliated Cancer Hospital of Zhengzhou University & Henan Cancer Hospital, Zhengzhou, Henan, P. R. China.

^8^Department of Respiratory Oncology, Shanxi Provincial Cancer Hospital, Taiyuan, Shanxi, P. R. China.

^9^Department of Respiratory and Critical Care Medicine, The First Affiliated Hospital of Bengbu Medical College, Bengbu, Anhui, P. R. China.

^10^Department of Respiratory Medical Oncology, The Affiliated Cancer Hospital of Guangxi Medical University, Nanning, Guangxi Zhuang Autonomous Region, P. R. China.

^11^Department of Oncology, The Affiliated Hospital of Chengde Medical University, Chengde, Hebei, P. R. China.

^12^Department of Medical Oncology, Meizhou People’s Hospital, Meizhou, Guangdong, P. R. China.

^13^Department of Clinical Science, Xuanzhu Biopharmaceutical Co., Ltd., Beijing, P. R. China.

^*^Address correspondence to: H.L. (Haifeng Liu), [haifengliu2025@126.com](mailto:haifengliu2025@126.com)

^†^These authors contributed equally to this work.

**Supplementary Table S1. Tumor response per investigator for the patients with measurable brain metastases at baseline**

| **Response** | **Dirozalkib**  **(*n* = 13)** | **Crizotinib**  **(*n* = 9)** |
| --- | --- | --- |
| Best overall response, *n* (%) | | |
| CR | 1 (7.7) | 0 (0.0) |
| PR | 11 (84.6) | 1 (11.1) |
| SD | 0 (0.0) | 4 (44.4) |
| PD | 1 (7.7) | 2 (22.2) |
| Not evaluable | 0 (0.0) | 2 (22.2) |
| ORR (95% CI), % | 92.3 (64.0-99.8) | 11.1 (0.3-48.3) |
| DoR (95% CI), months | 18.4 (7.0-NR) | 3.6 (NR-NR) |

**Abbreviations:** CR, complete response; PR, partial response; SD, stable disease; PD, disease progression; CI, confidence interval; ORR, objective response rate; DoR, duration of response; NR, not reached.

**Supplementary Table S2. Subsequent anticancer therapies after disease progression**

| **Subsequent anticancer therapy, *n* (%)** | **Dirozalkib**  **(*n* = 63)** | **Crizotinib**  **(*n* = 92)^a^** |
| --- | --- | --- |
| At least one subsequent anticancer therapy | 54 (85.7) | 83 (90.2) |
| **Type of therapy** | | |
| ALK inhibitor | 49 (77.8) | 76 (82.6) |
| Chemotherapy | 10 (15.9) | 7 (7.6) |
| Anti-angiogenic agent | 7 (11.1) | 8 (8.7) |
| Immune checkpoint inhibitor | 3 (4.8) | 3 (3.3) |
| EGFR inhibitor | 1 (1.6) | 2 (2.2) |
| Anti-angiogenic agent plus chemotherapy | 1 (1.6) | 0 (0.0) |
| Antibody-drug conjugate | 0 (0.0) | 1 (1.1) |
| Other | 4 (6.3) | 8 (8.7) |

^a^Three patients in the crizotinib group who experienced death before disease progression were not included in the analysis of subsequent anticancer therapies after disease progression. **Abbreviations:** ALK, anaplastic lymphoma kinase; EGFR, epidermal growth factor receptor.

**Supplementary Table S3. TESAEs (≥1%) and TRSAEs in patients who received dirozalkib and crizotinib**

| **Adverse events, *n* (%)** | **Dirozalkib**  **(*n* = 137)** | **Crizotinib**  **(*n* = 138)** |
| --- | --- | --- |
| **TESAEs** | 29 (21.2) | 37 (26.8) |
| Hyperglycemia | 8 (5.8) | 0 (0.0) |
| ALT increased | 4 (2.9) | 5 (3.6) |
| AST increased | 3 (2.2) | 4 (2.9) |
| Vomiting | 3 (2.2) | 0 (0.0) |
| Nausea | 2 (1.5) | 0 (0.0) |
| Abdominal pain | 2 (1.5) | 0 (0.0) |
| Diarrhea | 2 (1.5) | 0 (0.0) |
| Fatigue | 2 (1.5) | 0 (0.0) |
| Muscle weakness | 2 (1.5) | 0 (0.0) |
| Pleural effusion | 1 (0.7) | 2 (1.4) |
| White blood cells decreased | 0 (0.0) | 2 (1.4) |
| Neutrophil count decreased | 0 (0.0) | 2 (1.4) |
| Sinus node dysfunction | 0 (0.0) | 2 (1.4) |
| Sinus bradycardia | 0 (0.0) | 2 (1.4) |
| Infectious pneumonia | 0 (0.0) | 3 (2.2) |
| Dyspnea | 0 (0.0) | 2 (1.4) |
| Drug-induced liver injury | 0 (0.0) | 2 (1.4) |
| Rash | 0 (0.0) | 2 (1.4) |
| Deep vein thrombosis | 0 (0.0) | 2 (1.4) |
| **TRSAEs** | 19 (13.9) | 17 (12.3) |
| Hyperglycemia | 7 (5.1) | 0 (0.0) |
| ALT increased | 4 (2.9) | 4 (2.9) |
| AST increased | 3 (2.2) | 3 (2.2) |
| Vomiting | 3 (2.2) | 0 (0.0) |
| Nausea | 2 (1.5) | 0 (0.0) |
| Abdominal pain | 2 (1.5) | 0 (0.0) |
| Diarrhea | 2 (1.5) | 0 (0.0) |
| Fatigue | 2 (1.5) | 0 (0.0) |
| White blood cells decreased | 0 (0.0) | 2 (1.4) |
| Neutrophil count decreased | 0 (0.0) | 2 (1.4) |
| Sinus bradycardia | 0 (0.0) | 2 (1.4) |
| Drug-induced liver injury | 0 (0.0) | 2 (1.4) |

**Abbreviations:** TESAE, treatment-emergent serious adverse event; TRSAE, treatment-related serious adverse event; ALT, alanine aminotransferase; AST, aspartate aminotransferase.

**Supplementary Table S4.** **TEAEs (≥1%) leading to dose interruption, reduction, and discontinuation in patients who received dirozalkib and crizotinib**

| **Adverse events, *n* (%)** | **Dirozalkib**  **(*n* = 137)** | **Crizotinib**  **(*n* = 138)** |
| --- | --- | --- |
| **TEAE leading to dose interruption** | 61 (44.5) | 78 (56.5) |
| COVID-19 infection | 16 (11.7) | 17 (12.3) |
| Diarrhea | 14 (10.2) | 1 (0.7) |
| Vomiting | 8 (5.8) | 2 (1.4) |
| Abdominal pain | 7 (5.1) | 0 (0.0) |
| Nausea | 6 (4.4) | 1 (0.7) |
| Fatigue | 4 (2.9) | 1 (0.7) |
| QT interval prolongation | 4 (2.9) | 9 (6.5) |
| Hyperglycemia | 4 (2.9) | 0 (0.0) |
| Sinus bradycardia | 2 (1.5) | 25 (18.1) |
| Fever | 2 (1.5) | 2 (1.4) |
| $\gamma$-glutamyltransferase increased | 2 (1.5) | 1 (0.7) |
| Palpitations | 2 (1.5) | 1 (0.7) |
| Anemia | 2 (1.5) | 1 (0.7) |
| Hypertriglyceridemia | 2 (1.5) | 0 (0.0) |
| Neutrophil count decreased | 1 (0.7) | 33 (23.9) |
| Blood creatine kinase increased | 1 (0.7) | 10 (7.2) |
| White blood cells decreased | 1 (0.7) | 7 (5.1) |
| AST increased | 1 (0.7) | 4 (2.9) |
| ALT increased | 1 (0.7) | 3 (2.2) |
| Infectious pneumonia | 1 (0.7) | 2 (1.4) |
| Chest pain | 1 (0.7) | 2 (1.4) |
| Blood CK-MB increased | 0 (0.0) | 5 (3.6) |
| Edema | 0 (0.0) | 2 (1.4) |
| Dyspnea | 0 (0.0) | 2 (1.4) |
| Deep vein thrombosis | 0 (0.0) | 2 (1.4) |
| Upper respiratory tract infection | 0 (0.0) | 2 (1.4) |
| **TEAE leading to dose reduction** | 27 (19.7) | 42 (30.4) |
| ALT increased | 6 (4.4) | 12 (8.7) |
| Diarrhea | 5 (3.6) | 0 (0.0) |
| Vomiting | 5 (3.6) | 0 (0.0) |
| AST increased | 4 (2.9) | 9 (6.5) |
| Hyperglycemia | 4 (2.9) | 0 (0.0) |
| $\gamma$-glutamyltransferase increased | 3 (2.2) | 0 (0.0) |
| Nausea | 3 (2.2) | 0 (0.0) |
| Decreased appetite | 2 (1.5) | 0 (0.0) |
| Fatigue | 2 (1.5) | 0 (0.0) |
| Anemia | 2 (1.5) | 0 (0.0) |
| QT interval prolongation | 1 (0.7) | 6 (4.3) |
| Sinus bradycardia | 0 (0.0) | 15 (10.9) |
| Blood CK-MB increased | 0 (0.0) | 2 (1.4) |
| Neutrophil count decreased | 0 (0.0) | 2 (1.4) |
| Drug-induced liver injury | 0 (0.0) | 2 (1.4) |
| **TEAE leading to study drug discontinuation** | 3 (2.2) | 10 (7.2) |
| Infectious pneumonia | 0 (0.0) | 2 (1.4) |

**Abbreviations:** TEAE, treatment-emergent adverse event; ALT, alanine aminotransferase; AST, aspartate aminotransferase; CK-MB, creatine kinase-MB; COVID-19, coronavirus disease 2019.
